# Base‐Assisted Imidization: A Synthetic Method for the Introduction of Bulky Imide Substituents to Control Packing and Optical Properties of Naphthalene and Perylene Imides

**DOI:** 10.1002/anie.202004965

**Published:** 2020-05-27

**Authors:** Magnus Mahl, Kazutaka Shoyama, Ana‐Maria Krause, David Schmidt, Frank Würthner

**Affiliations:** ^1^ Institut für Organische Chemie Universität Würzburg Am Hubland 97074 Würzburg Germany; ^2^ Center for Nanosystems Chemistry (CNC) Universität Würzburg Theodor-Boveri-Weg 97074 Würzburg Germany

**Keywords:** dyes, fluorescence, imidization, perylene imide, solid-state emitters

## Abstract

We report the direct imidization of naphthalene and perylene dicarboxylic anhydrides/esters with bulky *ortho*,*ortho*‐diaryl‐ and *ortho*,*ortho*‐dialkynylaniline derivatives. This imidization method uses *n*‐butyllithium as a strong base to increase the reactivity of bulky amine derivatives, proceeds under mild reaction conditions, requires only stoichiometric amounts of reactants and gives straightforward access to new sterically crowded rylene dicarboximides. Mechanistic investigations suggest an isoimide as intermediary product, which was converted to the corresponding imide upon addition of an aqueous base. Single‐crystal X‐ray diffraction analyses reveal dimeric packing motifs for monoimides, while two‐side shielded bisimides crystallize in isolated molecules without close π–π‐interactions. Spectroscopic investigations disclose the influence of the bulky substituents on the optical properties in the solid state.

## Graphical abstract

**It's all about the sterics**: A base‐mediated imidization procedure under mild reaction conditions and stoichiometric amounts of reactants gives direct and easy access to sterically crowded naphthalene/perylene imides. The effects of the steric demand of the different substituents on the packing motifs and optical properties in the solid state are explored.

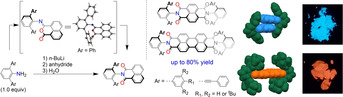

## Introduction

Rylene dicarboximides are a subject of intense research for materials scientists owing to their high absorptivities and extraordinary stability.^1^, ^2^, ^3^, ^4^, ^5^, ^6^, ^7^, ^8^ Originating from this research, rylene dicarboximides are applied as color pigments^1^ and fluorescence dyes^4^ in market products and investigated heavily for applications in organic electronics and photovoltaics.^6^, ^7^ Due to their strong tendency to aggregate through the π‐surface, introduction of bulky substituents to inhibit intermolecular interactions is indispensable for the investigation of their intrinsic molecular properties. In early studies, this was achieved by the introduction of long and branched alkyl chains^9^ or aryl substituents such as 2,6‐diisopropylphenyl or 2,5‐di‐*tert*‐butylphenyl groups^10^, ^11^ at the imide positions of perylene bis(dicarboximides) (PBIs). Furthermore, by introducing much bulkier aryl groups, such chromophores can crystallize in an isolated packing arrangement, allowing their use as solid‐state emitters.^12^, ^13^ For PBIs bulky substituents can be introduced either at the bay‐positions,^14^, ^15^ *ortho*‐positions^16^, ^17^ or the imide‐positions.^12^, ^13^, ^18^, ^19^ The last is ideal for preserving the original properties of perylene dyes^13^ and allows the introduction of additional substituents at the rylene scaffold to tune the optical and the electronic characteristics.^20^ The *N*‐substituents of rylene dicarboximides are often introduced by imidization of corresponding rylene dicarboxylic acid anhydride and an excess of amine, typically in the presence of zinc(II) salts, in solvents with high‐boiling points at elevated temperatures up to 180 °C.^10^ However, such direct introduction of large substituents is hampered by the low reactivity of the amine substrate as already seen in the imidization using 2,4,6‐triphenylaniline and perylene bisanhydride, in which even under extremely harsh reaction conditions did not yield the desired product but only decomposition of the anhydride.^18^

Herein, we report the direct synthesis of naphthalene and perylene (dicarboximides) with sterically demanding *ortho*,*ortho*‐diarylphenyl or *ortho*,*ortho*‐dialkynylphenyl imide groups. Direct imidization was achieved by using stoichiometric amounts of amine and *n*‐butyllithium (*n*‐BuLi) as a strong base to deprotonate the bulky amine substrate. Crystallographic analyses disclosed the impact of sterically demanding imide‐substituents on the packing motifs and elucidated solid‐state structure–property relationships.

## Results and Discussion

The reaction of 2,6‐bis(3,5‐di‐*tert*‐butylphenyl)aniline (**1 d**) as sterically demanding amine and naphthalene‐1,8‐dicarboxylic anhydride (**2**) was used as a model reaction (Table 1 and Table S1, Supporting Information). In the initial investigation, we used lithium diisopropyl amide (LDA) as a base to deprotonate the amine. Thus, as the first step **1 d** (1.0 equiv) and LDA (2.0 equiv) were reacted in THF at −78 °C. Subsequently, the mixture was allowed to warm up to room temperature, followed by the addition of **2** (1.0 equiv) and refluxed for 18 h. This one‐pot reaction yielded the desired product **6 d** only in 2 % yield (entry 1). We found that isoimide was formed under comparable conditions and addition of aqueous base was required to convert the isoimide into the corresponding imide (see below). Therefore, with addition of water (5.0 equiv) after 6 h in the second step **6 d** was obtained in 42 % yield (entry 2).

**Table 1:** Optimization of the reaction conditions for **6 d**.^[a]^ 
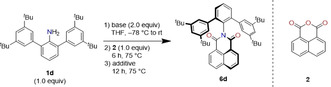

entry	base	additive (equiv)	yield [%]^[b]^
1	LDA	–	2
2	LDA	H_2_O (5.0)	42
3	LDA	H_2_O (1.0)	18
4	LDA	H_2_O (100)	40
5^[c]^	LDA	H_2_O (5.0)	39
6^[d]^	LDA	H_2_O (5.0)	18
7^[e]^	LDA	H_2_O (5.0)	11
8^[e]^	LDA	–	13
9	P4‐*t*‐Bu	H_2_O (5.0)	63
10	*n*‐BuLi	H_2_O (5.0)	80
11	*sec*‐BuLi	H_2_O (5.0)	44
12	*tert*‐BuLi	H_2_O (5.0)	51

[a] Reaction conditions: **1 d** (0.1 mmol), base (2.0 equiv), **2** (1.0 equiv), *c*(**1 d**)=0.07 M. [b] Isolated yields. [c] Addition of water after 12 h of heating. Reaction time 36 h for the third step. [d] Addition of water after 1 h. [e] Use of naphthalene‐1,8‐dicarboxylic acid dimethyl ester instead of **2**. LDA=lithium diisopropylamide, BuLi=butyllithium, THF=tetrahydrofuran.

In the pursuit of higher product yields, we investigated the amount of water (entries 2–4) as well as the point of addition (entries 5 and 6). Neither a higher loading of water nor longer reaction time before the addition of water lead to significant improvement. The use of the more soluble naphthalene‐1,8‐dicarboxylic acid dimethyl ester instead of **2** lead with and without the addition of water to decreased but similar yields of 11 % and 13 %, respectively (entries 7 and 8). These results hint at the formation of isoimide when using the dicarboxylic anhydride **2**, which is improbable when applying the corresponding methyl ester. Next, we tested different strong bases including superbases^21^ and combinations of two bases (entries 9–12, and Table S1). The bulky Schwesinger‐base^22^ P4‐*t*‐Bu improved the yield to 63 % (entry 9). Moreover, the use of various isomeric butyllithium bases also improved the yield compared to LDA (entries 10–12). The best yield of 80 % was obtained when *n*‐BuLi was used as the base (entry 10).

**Scheme 1:**
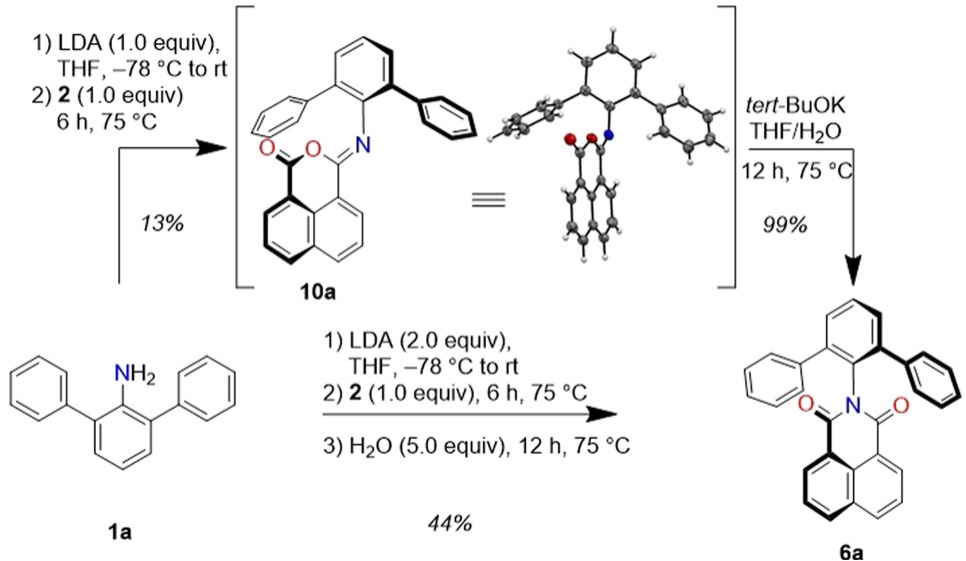
Reaction pathways towards **6 a**.

As known from literature, steric restrictions or electronic effects can lead to formation of isoimides instead of imides. These isoimides suffer from low stability and their isolation was scarce.^23^, ^24^ Such isoimides can be transformed easily through a rearrangement into the corresponding imides with aqueous acid or base.^23^, ^25^ Therefore, we hypothesized that during the applied reaction conditions, unstable isoimide was formed due to the high steric demand of the 2,6‐substituents, which rearranged through the addition of water into the corresponding imide. While in the conducted reactions during the optimization as well as in the substrate scope, no isoimide intermediates were observed, we were able to isolate isoimide **10 a** under slightly modified reaction conditions with LDA (1.0 equiv) and no addition of water (Scheme 1). The isoimide structure could be unambiguously confirmed by crystallographic analysis. Isoimide **10 a** could be quantitatively transformed under basic conditions in THF (*tert*‐BuOK_aq_) into the corresponding imide **6 a**. These results support our reaction pathway rationale for the imide formation via an isoimide intermediate when the respective anhydride is used as starting material.

**Scheme 2:**
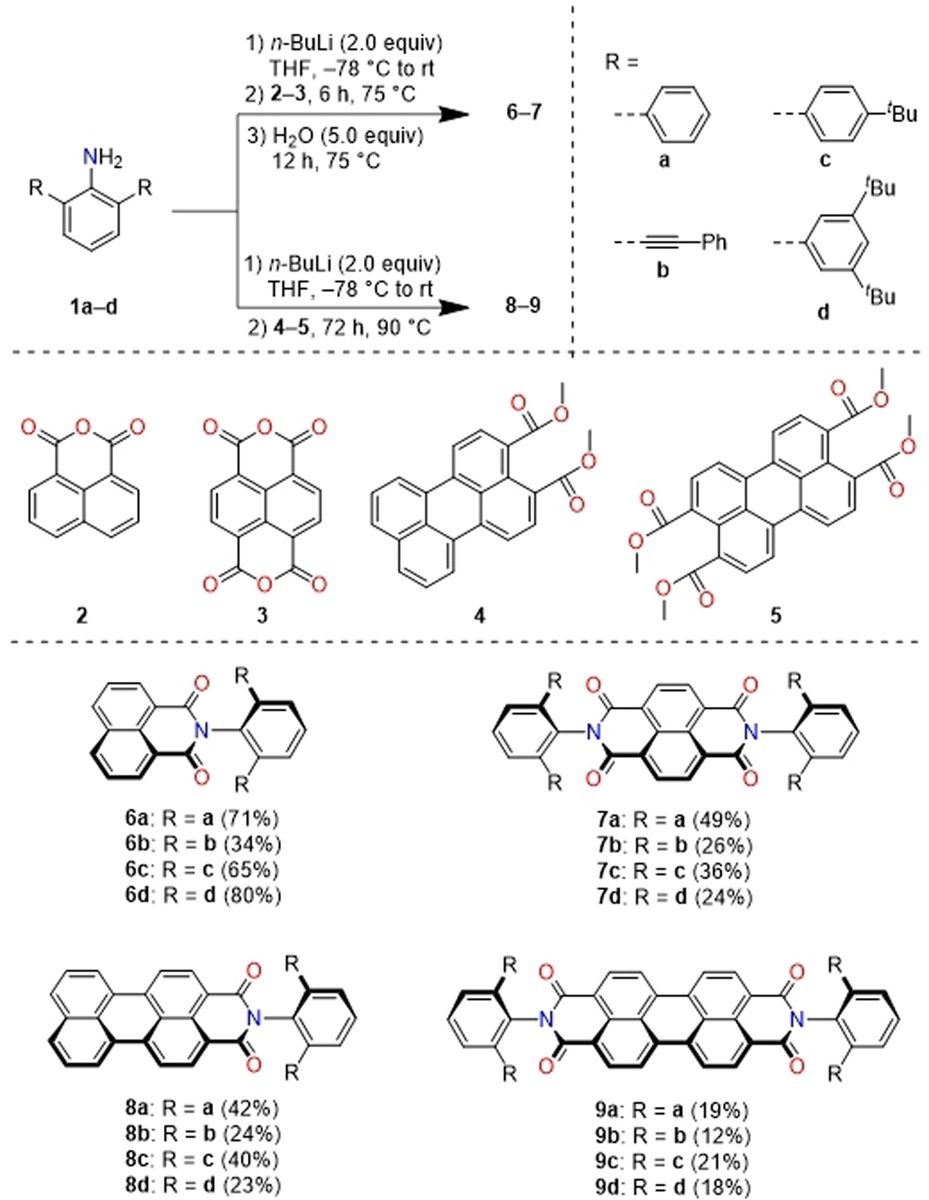
Substrate scope of the *n*‐butyllithium‐mediated imidization reaction (details in Supporting Information).

Using the optimized reaction conditions, we examined the substrate scope of this imidization (Scheme 2). Sterically demanding aryl amines **1 a**–**c** were used for the imidization reaction with naphthalene dicarboxylic anhydride **2**. The respective products **6 a**–**c** could be isolated in comparable yields to **6 d** for **6 a** (71 %) and **6 c** (65 %), but a reduced yield for **6 b** (34 %). In all reactions most of the remaining amines could be recovered. Furthermore, we tested the more challenging substrate naphthalene bisanhydride **3**. The expected low reactivity due to the close proximity of the bulky substituents in the desired products was reflected in the reaction yields. While **7 a** could be isolated in a good yield of 49 %, the yields for bulkier substituents in **7 b**–**d** decreased to 24–36 %. To our disappointment, the imidization with perylene mono and bisanhydrides afforded only starting material or traces of products, which may be due to their lower solubility. This could be overcome by the use of corresponding perylene methyl esters^26^ **4** and **5** (Table S2) with an increased reaction temperature of 90 °C (pressure‐stable Schlenk‐tube) and a longer reaction time of 72 h without the need of water addition. With these modifications new perylene mono(dicarboximides) **8 a**–**d** were obtained in moderate yields of 23–42 %. Two‐fold imidization of perylene tetramethyl ester with **1 a**–**d** yielded the respective products in 12–21 %. All dicarboximides were fully characterized by ^1^H and ^13^C NMR spectroscopy as well as high‐resolution mass spectrometry (Supporting Information).

**Figure 1:**
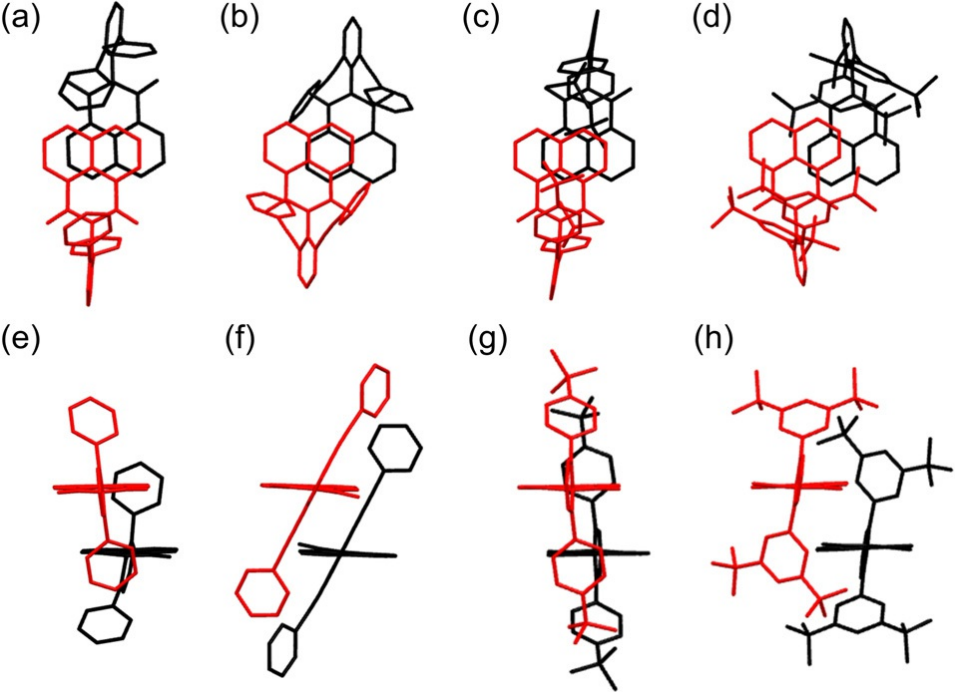
Crystal structures of a,e) **6 a**, b,f) **6 b**, c,g) **6 c**, d,h) **6 d** as obtained by single‐crystal X‐ray diffraction analysis.^40^

**Figure 2:**
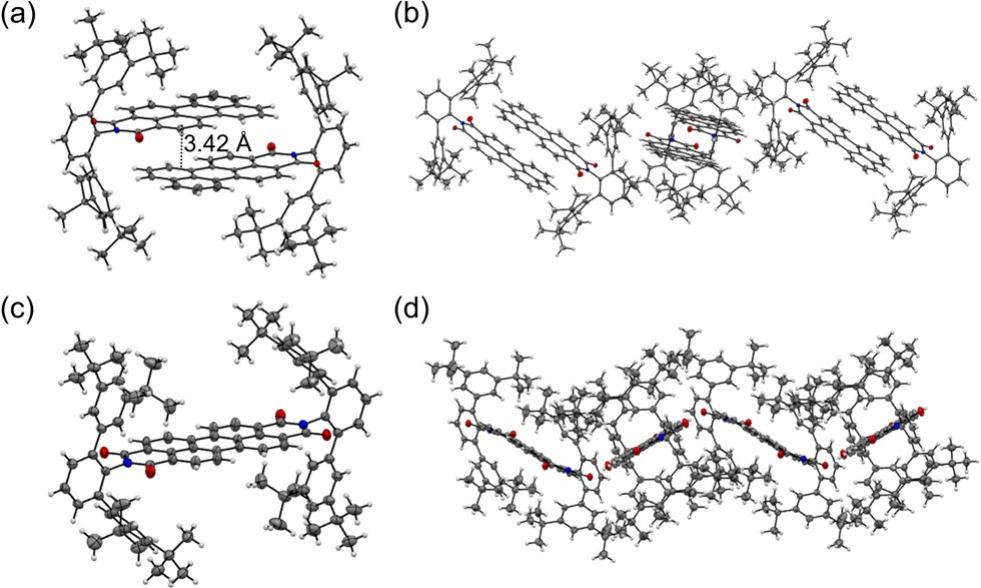
Crystal structures of a,b) **8 d** and c,d) **9 d**. ORTEP drawing at 50 % probability for thermal ellipsoids.^40^

X‐ray crystallography was used to elucidate the influence of bulky imide substituents on the solid‐state packing. Single crystals suitable for X‐ray diffraction were grown by slow diffusion of methanol or *n*‐hexane into dichloromethane or chloroform solutions and were obtained for **6 a**–**d**, **8 a**, **8 d**, **9 b** and **9 d** (Figures 1 and 2, and the Supporting Information). For the mono(dicarboximides) dimer packing motifs are observed. Within the series of naphthalene mono(dicarboximides) **6 a**–**d** the naphthalene units are more displaced and hence had decreased π–π‐contact with increasing bulkiness of the imide substituent (Figure 1). As we will show later, this variation in packing arrangement has a pronounced impact on the optical properties in the solid state.^27^

As shown in Figure 2 a,b similar packing motifs are observed for perylene monoimide **8 d**, however, with a much larger π–π‐contact surface due to the enlarged perylene π‐scaffold. In contrast, the crystal structures of perylene bis(dicarboximides) **9 b** and **9 d** show no direct π–π‐contact for the perylene cores (Figure 2c,d and Figure S7), which can be attributed to bilateral shielding with large substituents and is in accordance with the results of Wong and co‐workers.^13^ Therefore, bulky imide‐substituents are a suitable tool to effectively control and prevent close π–π‐interactions.

The UV/Vis absorption and fluorescence emission spectra of the imides **6**–**9** in chloroform solution show the characteristic vibronic patterns of the monomeric dyes (Figures S10–13, Tables S11–14).^4^, ^6^ While perylene imides **8** and **9** are highly fluorescent with quantum yields close to unity, naphthalene imides **6** and **7** show negligible fluorescence in solution (*Φ*_fl_ <0.5 %), which is due to deactivation through close triplet states.^28^, ^29^

**Figure 3:**
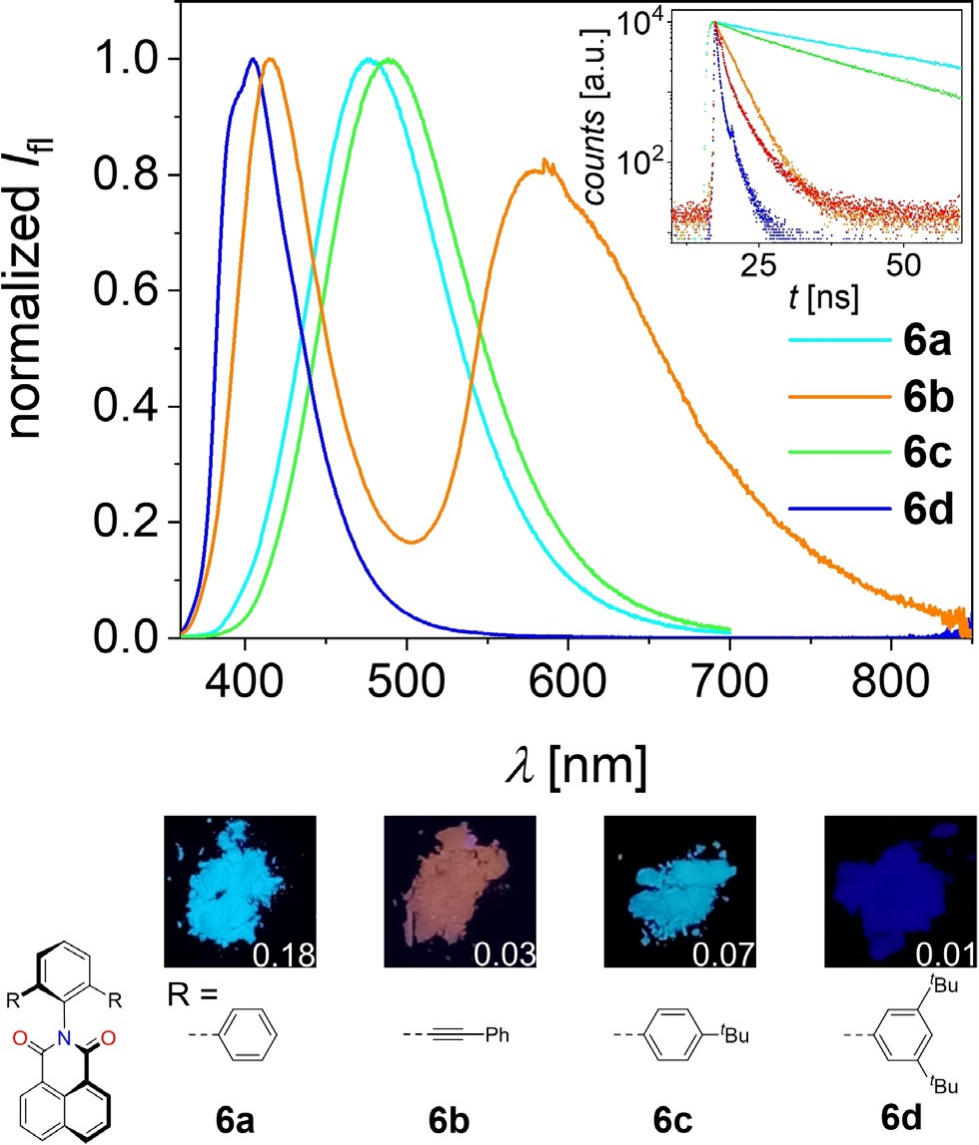
Solid‐state (powder) fluorescence spectra of **6 a**–**d** (*λ*_ex_=300 nm). Inset: Excerpts of lifetime‐decays of **6 a** (cyano), **6 b** (orange: *λ*_det_=416 nm; red: *λ*_det_=578 nm), **6 c** (green) and **6 d** (blue). Values in photographs are respective effective *Φ*_fl_.

Pronounced effects of the bulky *N*‐substituents were observed in the solid‐state optical properties. The absorption spectra of spin‐coated thin‐films of the imides largely resemble their respective solution spectra (Figures S14 and S15, Table S15). The vibronic structure becomes sharper with increased bulkiness of the substituents. This suggests a more effective shielding of the rylene cores and less intermolecular couplings.^30^ While naphthalene bis(dicarboximides) **7** are non‐emissive in the solid‐state, the respective mono(dicarboximides) **6** show solid‐state luminescence (Figure 3) with remarkably high quantum yields up to 18 % (**6 a**) (Table 2). Interestingly, the observed emission spectra are highly dependent on the nature of the imide substituent. While **6 a** and **6 c** show a rather broad and structureless emission with a pronounced Stokes shift and a cyan hue, **6 d** with the most steric demanding imide substituent shows a sharp emission profile centered at 405 nm, resulting in a dark‐blue fluorescence. The luminescence color of **6 b** is orange due to two emission bands at 415 and 582 nm. We attribute the pronounced Stokes shift observed for **6 a** and **6 c** to a relaxation into excimers which originate from the “preformed”^31^ dimers in the solid state (Figure 1). It is known that such “excimerization” is supported by nearly parallel alignments^32^, ^33^ and a significant π–π‐contact surface as found in the crystals of these dyes (Figure 1 a,c).^34^ It is interesting to note that this relaxation process of photoexcited **6 a**,**c** into excimers affords an increase of the fluorescence quantum yield by a factor greater than 30 for **6 a** compared to the monomeric state in solution. Similar observations were made for naphthalene dicarboximide‐based systems.^35^, ^36^ This formation of excimers is further supported by strongly increased fluorescence lifetimes up to 30.5 ns (**6 a**) in the solid state (Table S17) compared to monomeric naphthalene monodicarboximides in solution reported in the literature (typically <0.5 ns).^29^, ^33^ In contrast, the structured luminescence of **6 d** at shorter wavelengths and with short lifetimes can be attributed to an emission from a monomeric species, which is attributed to the rather small overlap of the naphthalimide moieties in the solid state (Figure 1 d). To elucidate the origin of the more complicated dual emission of **6 b**, we performed additional excitation‐dependent fluorescence and excitation spectroscopy (Figure S17). These studies revealed two independent radiative processes (Table S17). We assume that the fluorescence located at longer wavelengths (around 582 nm) originates from an intramolecular charge‐transfer state (CT). Indeed, a weak CT band is already observed in the UV/Vis spectra of **6 b** for dissolved dye monomers in solution as well as in the solid state (Figures S10 and S14 b). Accordingly, these absorption and fluorescence bands are attributed to the interaction between electron‐rich phenyleneethynylene imide substituents and electron‐poor naphthalene dicarboximide moiety. An excitation in the CT‐region substantially deactivates the first emission maxima attributable to the locally excited state of the naphthalene imide, supporting this assumption (Figure S17 b).

**Table 2:** Comparison of fluorescence maxima and fluorescence quantum yields in chloroform solution and bulk powder samples of **6** and **9**.

	*λ*_em, max_ [nm] (CHCl_3_)	*λ*_em, max_ [nm] (powder)	*Φ*_fl_ [%]^[a]^ (CHCl_3_)	*Φ*_fl_ [%]^[b]^ (powder)
**6 a**	–	478	<0.5	18
**6 b**	–	415, 582	<0.5	3
**6 c**	–	490	<0.5	7
**6 d**	–	405	<0.5	1
**9 a**	535, 577, 627	670	95±2	5
**9 b**	534, 576, 624	653	96±1	3
**9 c**	534, 577, 626	670	97±1	5
**9 d**	533, 577, 625	585, 640	93±2	17

[a] Fluorescence quantum yields were determined using the relative method (*A*<0.05) and *N*,*N*′‐bis(2,6‐diisopropylphenyl)perylene‐3,4:9,10‐bis(dicarboximide) (*Φ*_fl_ (CHCl_3_)=1.00) as reference. [b] Effective fluorescence quantum yields of bulk powder samples were determined using an integration sphere and represent the lower limit of the intrinsic fluorescence quantum yields due to reabsorption effects.

Intense solid‐state fluorescence was also observed for perylene bis(dicarboximides) **9** (Table 2) with quantum yields ranging between 3 % (**9 b**) and 17 % (**9 d**). We note that these solid‐state fluorescence spectra are likely affected by reabsorption losses, in particular for the (0,0) fluorescence band, which is known for dyes with small Stokes shifts and strong absorbance. Therefore, the obtained effective fluorescence quantum yields represent the lower limit of the intrinsic quantum yields.^15^ The strongest fluorescent PBI **9 d** exhibits also the most pronounced vibronic progression in its solid‐state fluorescence spectrum (Figure S16). This can be ascribed to emission from isolated monomer‐like species due to the largest steric demand of the imide substituent, thereby impeding any π–π‐contact between the perylene bisimide π‐scaffolds of neighbouring dyes (Figure 2 c,d). In contrast, perylene mono(dicarboximides) **8** are either non‐emissive (**8 a** and **8 b**) or only weakly fluorescent (quantum yields of 1 % and 2 % for **8 c** and **8 d**, respectively) in the solid state (Table S15). This can be attributed to an insufficient shielding of the extended π‐surfaces (Figure 2 and Figures S5 b and S6 b) whose contact area is quite extended and enables relaxation pathways into excimers^37^ or other non‐emissive excited states.^38^

## Conclusion

In summary, we described for the first time a direct one‐pot synthetic method for naphthalene and perylene dicarboximides bearing bulky imide substituents. With the use of *n*‐BuLi as a strong base the low reactivity of the bulky amine substrates could be overcome. When dicarboxylic anhydrides are applied as the starting material, we propose a reaction pathway via an isoimide–imide rearrangement induced by the addition of water. This hypothesis is supported by the isolation of an isoimide and its subsequent transformation to the respective imide. In comparison to the typically quite harsh conditions for imidizations, the reaction proceeds under mild conditions and only stoichiometric amounts of starting materials are needed. A broad substrate scope showed the versatility of the new method. The new imide molecules reveal highly *N*‐substituent‐dependent emission properties in the solid‐state, which originates from subtle differences in the packing arrangement as revealed by crystallographic analyses. This demonstrates the utility of sterically demanding imide substituents to control the solid‐state properties of this class of rylene imide dyes. The application of these and other sterically demanding imide substituents for solid‐state emitters,^13^, ^15^ in solar light concentrators^11^, ^12^ and in photonic devices^14^, ^39^ is expected. We further expect that our new method might be applicable for the functionalization with larger dendrons for solubilization of theses dyes in water,^5^ for example, through click‐chemistry of alkyne‐containing moieties.

## Conflict of interest

The authors declare no conflict of interest.

## Supporting information

As a service to our authors and readers, this journal provides supporting information supplied by the authors. Such materials are peer reviewed and may be re‐organized for online delivery, but are not copy‐edited or typeset. Technical support issues arising from supporting information (other than missing files) should be addressed to the authors.

Supplementary
