# Supplementary material for: Base‐Assisted Imidization: A Synthetic Method for the Introduction of Bulky Imide Substituents to Control Packing and Optical Properties of Naphthalene and Perylene Imides
Source: Angew Chem Int Ed Engl. 2020 May 27;59(32):13401–5. doi: 10.1002/anie.202004965 (PMC7496529; doi:10.1002/anie.202004965)
Supplement: Supplementary file 1 — Supplementary [file ANIE-59-13401-s001.pdf]

Angewandte Chemie
Eine Zeitschrift der Gesellschaft Deutscher Chemiker

Supporting Information

Base-Assisted Imidization: A Synthetic Method for the Introduction of Bulky Imide Substituents to Control Packing and Optical Properties of Naphthalene and Perylene Imides

*Magnus Mahl, Kazutaka Shoyama, Ana-Maria Krause, David Schmidt, and Frank Würthner**

anie_202004965_sm_miscellaneous_information.pdf

Table of Contents

General Methods 3

Optimization 4

Synthesis 6

Single crystal X-ray analysis 11

Optical properties in solution 23

Optical properties in the solid-state 27

NMR spectra 33

Mass spectra 52

References 58

General Methods

Unless otherwise stated, all chemicals, reagents and solvents were purchased from commercial suppliers and used without further purification. Column chromatography was performed on silica-gel (particle size 0.040-0.063 mm) and freshly distilled solvents. ^{1}H and ^{13}C NMR spectra were recorded on a Bruker Avance HD III 400 spectrometer and ^{13}C NMR spectra are broad band proton decoupled. Chemical shift (δ) data are listed in parts per million (ppm), are reported relative to tetramethylsilane and referenced internally to residual proton solvent resonances or natural abundance carbon resonances. Coupling constants (J) are reported in Hertz (Hz). MALDI TOF measurements were carried out on a Bruker Daltonics autoflex II LRF or Bruker Daltonics ultrafleXtreme mass spectrometer. ESI TOF spectra were acquired on a Bruker Daltonics micOTOF focus mass spectrometer. EI mass spectra were measured on a Finnigan MAT90. Melting points were measured on an Olympus BX41 polarization microscope and are uncorrected. All spectroscopic measurements were carried out using spectroscopic grade solvents. Thin-films samples were prepared by spin-coating 10^{-2} – 10^{-3} M chloroform solutions (due to low solubility: 1.2×10^{-4} M for **8a**) onto quartz substrates (50 µL, 3000 rpm, 30 s). Subsequently, the quartz substrates were annealed for 5 min at 130 °C on a precision hot plate from Harry Gestigkeit PZ28-2. UV/Vis absorption spectra in solution were recorded on a Jasco V670 or Jasco V-770-ST spectrometer. For solid-state UV/Vis measurements a Perkin-Elmar 950 with an including integration sphere was used. Fluorescence measurements were conducted on an Edinburgh Instruments FLS980 fluorescence spectrometer. Solid-state fluorescence spectra were recorded with a front face sample holder. The fluorescence quantum yields in solution were determined by using the dilution method ($A < 0.05$) and *N,N*`-Bis[2,6-diisopropylphenyl]perylene-3,4:9,10-bis(dicarboximide) (Φ_f = 100% in chloroform) as a reference. Solid-state fluorescence quanatum yields were measured by using a Hamamatsu Photonics C9920-02 integration sphere. Single crystals suitable for X-ray diffraction were measured on a Bruker D8 Quest Diffractometer with a PhotonII detector using Cu K_{α} radiation. The structures were solved using direct methods, expanded with Fourier techniques and refined using the SHELX software package.[S1] All non-hydrogen atoms were refined anisotropically and hydrogen atoms were assigned idealized positions and were included in calculation of structures factors.
Compounds **1a**[S2], **1c**[S3], **1d**[S4], and **12**[S5] were synthesized according to literature.

Optimization

Table S1. Additional optimization for the reaction conditions for **6d**.[a]

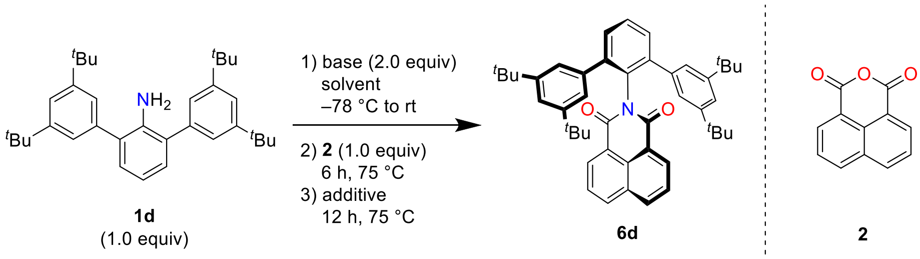

entry	base	solvent	additive (equiv)	yield [%][b]
1	LDA	THF	EtOH (5.0)	-
2	LDA	THF	10% HCl_{aq} (5.0)	42
3[c]	LDA	toluene	H_2O (5.0)	12
4[d]	LDA	DME	H_2O (5.0)	9
5[e]	LDA	diglyme	H_2O (5.0)	14
6[e]	LDA	DGDEE	H_2O (5.0)	7
7	LiTMP	THF	H_2O (5.0)	12
8	KHMDS	THF	H_2O (5.0)	-
9[f]	NaH	THF	H_2O (5.0)	-
10	LDA/KO^{t}Bu	THF	H_2O (5.0)	24
11	LDA/P4-*t*-Bu	THF	H_2O (5.0)	38
12	*n*-BuLi/KO^{t}Bu	THF	H_2O (5.0)	-
13	*n*-BuLi/P4-*t*-Bu	THF	H_2O (5.0)	52
14	*n*-BuLi/TMEDA	THF	H_2O (5.0)	54

[a] Reaction conditions: **1d** (0.1 mmol), base/base-solution (2.0 equiv), **2** (1.0 equiv), *c*(**1d**) = 0.07 M. [b] Isolated yields. [c] Reaction temperature 120 °C. [d] Reaction temperature 100 °C. [e] Reaction temperature 160 °C. [f] Deprotonation step at 75 °C instead of –78 °C. LDA: lithium diisopropylamide; LiTMP: lithium tetramethylpiperidide; KHMDS: potassium bis(trimethylsilyl)amide; BuLi: butyllithium; TMEDA: tetramethylethylenediamine; THF: tetrahydrofuran; DME: dimethoxyethane; diglyme: bis(2-methoxyethyl) ether; DGDEE: bis(2-ethoxyethyl) ether.

Table S2. Optimization for the reaction conditions for **9d**.[a]

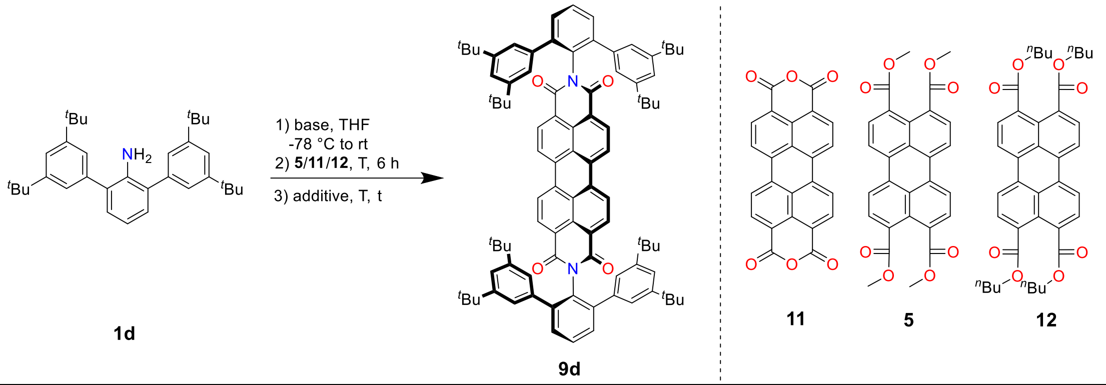

entry	starting material	base	equiv base	additive (equiv)	T [°C]	t [h]	yield [%][b]
1	**11**	LDA	4.0	H_2O (5.0)	75	24	-
2	**11**	*n*-BuLi	4.0	H_2O (5.0)	75	24	traces
3[c]	**11**	*n*-BuLi	4.0	H_2O (5.0)	75	72	-
4[d]	**11**	*n*-BuLi	4.0	H_2O (5.0)	75	24	-
5[e]	**11**	*n*-BuLi	4.0	H_2O (5.0) / $Zn(OAc)_2$ (0.3)	75	24	-
6[f]	**11**	*n*-BuLi	4.0	H_2O (5.0)	110	6	4
7[g]	**11**	*n*-BuLi	4.0	H_2O (5.0)	75	24	7
8[h]	**11**	*n*-BuLi	4.0	H_2O (5.0)	90	24	-
9[i]	**11**	*n*-BuLi	8.0	H_2O (10.0)	75	24	-
10	**5**	*n*-BuLi	4.0	-	75	24	14
11	**12**	*n*-BuLi	4.0	-	75	24	13
12[h]	**5**	*n*-BuLi	4.0	-	90	72	18
13[h]	**12**	*n*-BuLi	4.0	-	90	72	14
14[f]	**5**	*n*-BuLi	4.0	-	120	6	10
15[e], [h]	**5**	*n*-BuLi	4.0	DCC (4.0)	90	72	traces
16[h], [j]	**5**	*n*-BuLi	8.0	-	90	72	15

[a] Reaction conditions: **1d** (0.1 mmol), base-solution (2.0 equiv), **5**/**11**/**12** (0.5 equiv), 1.5 mL THF. [b] Isolated yields. [c] Addition of water after 24 h. [d] 3.0 mL THF. [e] Addition of additive with **5**/**12**. [f] Use of microwave. Additive after 2 h. [g] Use of sonication bath in the first 6 hours. [h] Use of pressure resistant Schlenk-tube. [i] Two-fold equivalents of base and additive. [j] Two-fold equivalents of amine and base. LDA: lithium diisopropylamide; BuLi: butyllithium; THF: tetrahydrofuran; DCC: *N*,*N*´-dicyclohexylcarbodiimide.

Synthesis

Perylene-3,4-dicarboxylic acid dimethyl ester (4)

Synthesis of this compound is already reported in literature.[S6] We used an alternative route for the synthesis of **4**.

Under nitrogen atmosphere perylene-3,4-dicarboxylic anhydride[S3] (948 mg, 2.94 mmol, 1.0 equiv) was suspended in methanol (50 mL) and acetonitrile (100 mL). Subsequently, methyl iodide (2.09 g, 14.7 mmol, 5.0 equiv) was added and the reaction mixture was cooled to 0 °C and 1,8-diazabicyclo[5.4.0]undec-7-ene (DBU) (1.79 g, 11.8 mmol, 4.0 equiv) was added dropwise. The reaction mixture was allowed to warm-up to room temperature and then heated to 65 °C for 6 d. The mixture was cooled to room temperature and carefully added 10% NH_3(aq)-solution (25 mL) to destroy excess of methyl iodide. The resulting mixture was extracted with dichloromethane. The organic phases were washed with water, dried over $MgSO_4$ and concentrated under reduced pressure. The crude product was purified by silica-gel column chromatography (chloroform) to yield **4** (831 mg, 2.26 mmol, 77%) as an orange solid. ^{1}H NMR (400 MHz, $CDCl_3$, 298 K): δ = 8.32 (dd, *J* =0.8 Hz, *J* = 7.7 Hz, 2H; Ar-*H*), 8.27 (d, *J* = 8.0 Hz, 2H; Ar-*H*), 8.04 (d, *J* = 8.0 Hz, 2H; Ar-*H*), 7.81 (dd, *J* =0.8 Hz, *J* = 8.3 Hz, 2H; Ar-*H*), 7.57 (t, *J* = 7.7 Hz, 2H; Ar-*H*), 3.93 ppm (s, 6H; *Ar*-H).

Perylene-3,4,9,10-tetracarboxylic acid tetramethyl ester (5)

Synthesis of this compound is already reported in literature.[S7] We used an alternative route for the synthesis of **5**.

Under nitrogen atmosphere perylene-3,4:9,10-tetracarboxylic dianhydride (4.00 g, 10.2 mmol, 1.0 equiv) was suspended in methanol (50 mL) and acetonitrile (100 mL). Subsequently, methyl iodide (14.5 g, 102 mmol, 10 equiv) was added and the reaction mixture was cooled to 0 °C and 1,8-diazabicyclo[5.4.0]undec-7-ene (DBU) (12.4 g, 81.6 mmol, 8.0 equiv) was added dropwise. The reaction mixture was allowed to warm-up to room temperature and then heated to 65 °C for 6 d. The mixture was cooled down to room temperature and carefully added 10% NH_3(aq)-solution (50 mL) to destroy excess of methyl iodide. The resulting mixture was extracted with dichloromethane. The organic phases were washed with water, dried over $MgSO_4$ and concentrated under reduced pressure. The crude product was purified by silica-gel column chromatography (chloroform) to yield **5** (4.21 g, 8.69 mmol, 85%) as an orange solid. ^{1}H NMR (400 MHz, $CDCl_3$, 298 K): δ = 8.37 (d, *J* = 8.0 Hz, 4H; Ar-*H*), 8.09 (d, *J* = 8.0 Hz, 4H; Ar-*H*), 3.95 ppm (s, 12H; *Ar*-H).

2,6-Bis(phenylethynyl)aniline (1b)

A Schlenk-tube was charged with $PdCl_2$ (136 mg, 767 µmol, 5.5 mol %), CuI (146 mg, 767 µmol, 5.5 mol %) and PPh_3 (146 mg, 1.53 mmol, 11 mol %) under an inert atmosphere and dry triethylamine (190 mL) was added. Subsequently, 2,6-dibromo aniline (3.50 g, 14.0 mmol, 1.0 equiv) and phenylacetylene (4.27 g, 41.8 mmol, 3.0 equiv) were added and the reaction mixture was stirred at 80 °C for 4 d. After the reaction mixture was cooled to room temperature, the solvent was removed under reduced pressure and the residue was dissolved in dichloromethane (75 mL). The solution was washed with water, dried over $MgSO_4$ and concentrated under reduced pressure. The crude product was purified by silica-gel column chromatography (*n*-hexane/dichlormethane = 10 /1) to give **1b** (2.24 g, 7.62 mmol, 55%) as a light-brown solid. m.p. 106–107 °C. ^{1}H NMR (400 MHz, $CDCl_3$, 298 K): δ = 7.51–7.57 (m, 4H; Ar-*H*), 7.34–7.40 (m, 8H; Ar-*H*), 6.69 (t, *J* = 7.7 Hz, 1H; Ar-*H*), 4.92 ppm (s, 2H; N*H*). ^{13}C NMR (101 MHz, $CDCl_3$, 298 K): δ = 149.0, 132.5, 131.6, 128.5, 128.4, 123.2, 117.4, 107.6, 95.1, 85.5 ppm. MS (EI, pos. mode): *m*/*z*: 293.1 [*M*]$^+$. HRMS (ESI-TOF, pos. mode, acetonitrile/chloroform 1/1): *m*/*z* calcd for $C_{22}H_{15}N+Na^+$: 316.1102 [*M*+Na]$^+$; found: 316.1093.

General method A

In a Schlenk-tube the respective amine **1a**–**d** (1.0 equiv) was dissolved in dry THF under a nitrogen atmosphere and cooled to –78 °C. Subsequently, 1.6 M *n*-BuLi solution in *n*-hexane (2.0 equiv) was added and the reaction mixture was stirred for 1 h at –78 °C. The solution was allowed to warm-up to room temperature and the respective anhydride **2**–**3** (1.0 equiv) was added. The mixture was heated to 75 °C for 6 h and water (5.0 equiv) was added. The reaction mixture was stirred for another 12 h at 75 °C and then cooled to room temperature. The resulting mixture was concentrated under reduced pressure. The crude product was purified by silica-gel column chromatography to obtain the respective product. For two-fold imidization equivalents of amine **1a**–**d**, *n*-BuLi and water were doubled.

General method B

In a pressure-stable Schlenk-tube the respective amine **1a**–**d** (1.0 equiv) was dissolved in dry THF under a nitrogen atmosphere and cooled to –78 °C. Subsequently, 1.6 M *n*-BuLi solution (2.0 equiv) was added and the reaction mixture was stirred for 1 h at –78 °C. The solution was allowed to warm-up to room temperature and the respective ester **4**–**5** (1.0 equiv) was added. The mixture was heated to 90 °C for 72 hours (caution: Schlenk-tube under pressure) and then cooled to room temperature. The resulting mixture was concentrated under reduced pressure. The crude product was purified by silica-gel column chromatography to obtain the respective product. For two-fold imidization equivalents of amine **1a**–**d** and *n*-BuLi were doubled.

***N*-[2,6-(Diphenyl)phenyl]naphthalene-1,8-dicarboximide (6a)**

General method A: **1a** (50.0 mg, 204 µmol, 1.0 equiv), *n*-BuLi (255 µL, 1.6 M in *n*-hexane, 408 µmol, 2.0 equiv), **2** (40.4 mg, 204 µmol, 1.0 equiv), THF (2.9 mL) and water (18.3 µL, 1.02 mmol, 5.0 equiv) were used. The crude product was purified by silica-gel column chromatography (gradient of cyclo-hexane/dichloromethane from 3/1 to 1/2) and washed with *n*-hexane and methanol. The product was dried under high vacuum to give **6a** (61.3 mg, 144 µmol, 71%) as a white solid. m.p. 243–245 °C. ^{1}H NMR (400 MHz, $CDCl_3$, 298 K): δ = 8.39 (dd, *J* = 1.1 Hz, *J* = 7.4 Hz, 2H; Ar-*H*), 8.10 (dd, *J* = 1.1 Hz, *J* = 7.4 Hz, 2H; Ar-*H*), 7.57–7.65 (m, 3H; Ar-*H*), 7.47–7.49 (m, 2H; Ar-*H*), 7.32–7.36 (m, 4H; Ar-*H*), 7.10–7.16 (m, 4H; Ar-*H*), 7.05–7.10 ppm (m, 2H; Ar-*H*). ^{13}C NMR (101 MHz, $CDCl_3$, 298 K): δ = 164.3, 141.8, 139.4, 134.1, 131.59, 131.55, 131.4, 130.3, 129.0, 128.5, 128.4, 128.0, 127.3, 126.8, 122.3 ppm. UV/Vis ($CHCl_3$): λ_{max} (ε) = 351 nm (12300 M^{-1} cm^{-1}). MS (MALDI-TOF, pos. mode, DCTB in chloroform): *m*/*z*: 425.1 [*M*]$^+$. HRMS (ESI-TOF, pos. mode, acetonitrile/chloroform 1/1): *m*/*z* calcd for $C_{30}H_{19}NO_2$+Na^+: 448.1313 [*M*+Na]$^+$; found: 448.1302.

***N*-[2,6-Bis(phenylethynyl)phenyl]naphthalene-1,8-dicarboximide (6b)**

General method A: **1b** (50.0 mg, 170 µmol, 1.0 equiv), *n*-BuLi (213 µL, 1.6 M in *n*-hexane, 341 µmol, 2.0 equiv), **2** (33.8 mg, 170 µmol, 1.0 equiv), THF (2.4 mL) and water (15.3 µL, 852 µmol, 5.0 equiv) were used. The crude product was purified by silica-gel column chromatography (gradient of cyclohexane/dichloromethane from 1/1 to 1/2) and washed with *n*-hexane and methanol. The product was dried under high vacuum to give **6b** (27.8 mg, 58.7 µmol, 34%) as a light-yellow solid. m.p. 221–222 °C. ^{1}H NMR (400 MHz, $CDCl_3$, 298 K): δ = 8.74 (dd, *J* = 1.1 Hz, *J* = 7.3 Hz, 2H; Ar-*H*), 8.33 (dd, *J* = 1.1 Hz, *J* = 8.3 Hz, 2H; Ar-*H*), 7.82–7.86 (m, 2H; Ar-*H*), 7.74 (d, *J* = 7.7 Hz, 2H; Ar-*H*), 7.48–7.55 (m, 1H; Ar-*H*), 7.17 (tt, *J* = 2.3 Hz, *J* = 7.4 Hz, 2H; Ar-*H*), 7.06–7.11 (m, 4H; Ar-*H*), 7.00-7.04 ppm (m, 4H; Ar-*H*). ^{13}C NMR (101 MHz, $CDCl_3$, 298 K): δ = 163.6, 139.4, 134.5, 132.6, 132.0, 131.8, 1316, 128.9, 128.8, 128.5, 128.2, 127.2, 124.1, 122.9, 122.7, 94.1, 85.2 ppm. UV/Vis ($CHCl_3$): λ_{max} (ε) = 350 nm (10800 M^{-1} cm^{-1}). MS (MALDI-TOF, pos. mode, DCTB in chloroform): *m*/*z*: 473.1 [*M*]$^+$. HRMS (ESI-TOF, pos. mode, acetonitrile/chloroform 1/1): *m*/*z* calcd for $C_{34}H_{19}NO_2$+H^+: 474.1416 [*M*+H]$^+$; found: 474.1477.

***N*-[2,6-Bis(4-*tert*-butylphenyl)phenyl]naphthalene-1,8-dicarboximide (6c)**

General method A: **1c** (50.0 mg, 140 µmol, 1.0 equiv), *n*-BuLi (175 µL, 1.6 M in *n*-hexane, 280 µmol, 2.0 equiv), **2** (27.7 mg, 140 µmol, 1.0 equiv), THF (2.0 mL) and water (12.6 µL, 852 µmol, 5.0 equiv) were used. The crude product was purified by silica-gel column chromatography (gradient of cyclohexane/dichloromethane from 1/1 to 1/2) and washed with methanol. The product was dried under high vacuum to give **6c** (48.8 mg, 90.8 µmol, 65%) as a white solid. m.p. 251–252 °C. ^{1}H NMR (400 MHz, CD_2Cl_2, 298 K): δ = 8.36 (dd, *J* = 1.2 Hz, *J* = 7.3 Hz, 2H; Ar-*H*), 8.15 (dd, *J* = 1.1 Hz, *J* = 8.2 Hz, 2H; Ar-*H*), 7.59–7.68 (m, 3H; Ar-*H*), 7.47–7.49 (m, 2H; Ar-*H*), 7.19–7.23 (m, 4H; Ar-*H*), 7.11–7.15 (m, 4H; Ar-*H*), 1.08 ppm (s, 18H; Alkyl-*H*). ^{13}C NMR (101 MHz, $CDCl_3$, 298 K): δ = 164.5, 150.0, 141.7, 136.5, 133.8, 132.0, 131.5, 131.3, 130.1, 129.0, 128.4, 128.1, 126.7, 124.9, 122.5, 34.4, 31.2 ppm. UV/Vis ($CHCl_3$): λ_{max} (ε) = 351 nm (11300 M^{-1} cm^{-1}). MS (MALDI-TOF, pos. mode, DCTB in chloroform): *m*/*z*: 537.2 [*M*]$^+$. HRMS (ESI-TOF, pos. mode, acetonitrile/chloroform 1/1): *m*/*z* calcd for $C_{38}H_{35}NO_2$+H^+: 538.2746 [*M*+H]$^+$; found: 538.2741.

***N*-[2,6-Bis(3,5-di-*tert*-butylphenyl)phenyl]naphthalene-1,8-dicarboximide (6d)**

General method A: **1d** (50.0 mg, 106 µmol, 1.0 equiv), *n*-BuLi (133 µL, 1.6 M in *n*-hexane, 212 µmol, 2.0 equiv), **2** (21.1 mg, 106 µmol, 1.0 equiv), THF (1.5 mL) and water (9.6 µL, 532 µmol, 5.0 equiv) were used. The crude product was purified by silica-gel column chromatography (gradient of cyclohexane/dichloromethane from 3/1 to 1/1) and washed with methanol. The product was dried under high vacuum to give **6d** (55.3 mg, 85.1 µmol, 80%) as a white solid. m.p. 257–259 °C. ^{1}H NMR (400 MHz, $CDCl_3$, 298 K): δ = 8.33 (dd, *J* = 1.2 Hz, *J* = 7.3 Hz, 2H; Ar-*H*), 8.05 (dd, *J* = 1.1 Hz, *J* = 8.3 Hz, 2H; Ar-*H*), 7.56–7.62 (m, 3H; Ar-*H*), 7.51–7.54 (m, 2H; Ar-*H*), 7.21 (d, *J* = 1.9 Hz, 4H; Ar-*H*), 7.06 (t, *J* = 1.9 Hz, 2H; Ar-*H*), 1.04 ppm (s, 36H; Alkyl-*H*). ^{13}C NMR (101 MHz, $CDCl_3$, 298 K): δ = 164.0, 149.8, 142.5, 138.4, 133.5, 132.2, 131.2, 130.8, 129.5, 128.8, 128.1, 126.5, 123.0, 122.6, 120.6, 34.6, 31.1 ppm. UV/Vis ($CHCl_3$): λ_{max} (ε) = 351 nm (11400 M^{-1} cm^{-1}). MS (MALDI-

TOF, pos. mode, DCTB in chloroform): *m/z*: 649.3 [*M*]$^+$. HRMS (ESI-TOF, pos. mode, acetonitrile/chloroform 1/1): *m/z* calcd for $C_{46}H_{51}NO_2$+H$^+$: 650.3998 [*M*+H]$^+$; found: 650.3994.

_N,N`_-Bis[2,6-(diphenyl)phenyl]naphthalene-1,4:5,8-bis(dicarboximide) (7a)

General method A: **1a** (50.0 mg, 204 µmol, 2.0 equiv), *n*-BuLi (255 µL, 1.6 M in *n*-hexane, 408 µmol, 4.0 equiv), **3** (27.3 mg, 102 µmol, 1.0 eq equiv), THF (2.9 mL) and water (18.3 µL, 1.02 mmol, 10.0 equiv) were used. The crude product was purified by silica-gel column chromatography (gradient of cyclohexane/dichloromethane from 1/1 to 1/8) and washed with *n*-hexane and methanol. The product was dried under high vacuum to give **7a** (35.9 mg, 49.7 µmol, 49%) as a white solid. m.p. > 350 °C. ^{1}H NMR (400 MHz, CD_2Cl_2, 298 K): *δ* = 8.41 (s, 4H; Ar-*H*), 7.62–7.67 (m, 2H; Ar-*H*), 7.47–7.50 (m, 4H; Ar-*H*), 7.21–7.25 (m, 8H; Ar-*H*), 7.09–7.16 ppm (m, 12H; Ar-*H*). ^{13}C NMR (101 MHz, CD_2Cl_2, 298 K): *δ* = 163.2, 142.1, 139.2, 131.2, 131.1, 130.7, 129.6, 128.5, 128.4, 127.9, 127.1, 126.4 ppm. UV/Vis ($CHCl_3$): λ_{max} (ε) = 381 nm (24000 M^{-1} cm^{-1}). MS (MALDI-TOF, pos. mode, DCTB in chloroform): *m/z*: 722.1 [*M*]$^+$. HRMS (ESI-TOF, pos. mode, acetonitrile/chloroform 1/1): *m/z* calcd for $C_{50}H_{30}N_2O_4$+Na$^+$: 745.2103 [*M*+Na]$^+$; found: 745.2101.

_N,N`_-Bis[2,6-bis(phenylethynyl)pheny] naphthalene-1,4:5,8-bis(dicarboximide) (7b)

General method A: **1b** (50.0 mg, 170 µmol, 2.0 equiv), *n*-BuLi (213 µL, 1.6 M in *n*-hexane, 341 µmol, 4.0 equiv), **3** (22.8 mg, 85.2 µmol, 1.0 equiv), THF (2.4 mL) and water (15.3 µL, 852 µmol, 10.0 equiv) were used. The crude product was purified by silica-gel column chromatography (gradient of cyclohexane/dichloromethane from 1/1 to 1/10) and washed with *n*-hexane and methanol. The product was dried under high vacuum to give **7b** (18.3 mg, 22.3 µmol, 26%) as a light yellow solid. m.p. > 350 °C. ^{1}H NMR (400 MHz, $CDCl_3$, 298 K): *δ* = 8.94 (s, 4H; Ar-*H*), 7.75 (d, *J* = 7.8 Hz, 4H; Ar-*H*), 7.51–7.56 (m, 2H; Ar-*H*), 7.05–7.10 (m, 4H; Ar-*H*), 6.93–7.02 ppm (m, 16H; Ar-*H*). ^{13}C NMR (101 MHz, $CDCl_3$, 298 K): *δ* = 162.1, 138.0, 132.9, 131.7, 131.4, 129.5, 128.8, 128.3, 127.4, 127.1, 124.0, 122.3, 94.6, 84.8 ppm. UV/Vis ($CHCl_3$): λ_{max} (ε) = 378 nm (23100 M^{-1} cm^{-1}). MS (MALDI-TOF, pos. mode, DCTB in chloroform): *m/z*: 818.1 [*M*]$^+$. HRMS (ESI-TOF, pos. mode, acetonitrile/chloroform 1/1): *m/z* calcd for $C_{58}H_{30}N_2O_4$+Na$^+$: 842.2103 [*M*+Na]$^+$; found: 841.2094.

_N,N`_-Bis[2,6-bis(4-_tert_-butylphenyl)phenyl] naphthalene-1,4:5,8-bis(dicarboximide) (7c)

General method A: **1c** (50.0 mg, 140 µmol, 2.0 equiv), *n*-BuLi (175 µL, 1.6 M in *n*-hexane, 280 µmol, 4.0 equiv), **3** (27.7 mg, 69.9 µmol, 1.0 equiv), THF (2.0 mL) and water (12.6 µL, 852 µmol, 10.0 equiv) were used. The crude product was purified by silica-gel column chromatography (gradient of cyclohexane/dichloromethane from 1/1 to 1/5) and washed with methanol. The product was dried under high vacuum to give **7c** (23.8 mg, 25.1 µmol, 36%) as a white solid. m.p. > 350 °C. ^{1}H NMR (400 MHz, CD_2Cl_2, 298 K): *δ* = 8.42 (s, 4H; Ar-*H*), 7.60–7.65 (m, 2H; Ar-*H*), 7.46-7.49 (m, 4H; Ar-*H*), 7.09–7.17 (m, 16H; Ar-*H*), 1.08 ppm (s, 36H; Alkyl-*H*). ^{13}C NMR (101 MHz, $CDCl_3$, 298 K): *δ* = 163.0, 150.3, 141.9, 141.6, 136.2, 131.1, 130.8, 130.3, 129.5, 128.1, 126.2, 125.0, 34.5, 31.3 ppm. UV/Vis ($CHCl_3$): λ_{max} (ε) = 381 nm (24700 M^{-1} cm^{-1}). MS (MALDI-TOF, pos. mode, DCTB in chloroform): *m/z*: 946.4 [*M*]$^+$. HRMS (ESI-TOF, pos. mode, acetonitrile/chloroform 1/1): *m/z* calcd for $C_{66}H_{62}N_2O_4$+Na$^+$: 969.4607 [*M*+Na]$^+$; found: 969.4604.

_N,N`_-Bis[2,6-bis(3,5-di-_tert_-butylphenyl)phenyl]naphthalene-1,4:5,8-bis(dicarboximide) (7d)

General method A: **1d** (50.0 mg, 106 µmol, 2.0 equiv), *n*-BuLi (133 µL, 1.6 M in *n*-hexane, 212 µmol, 4.0 equiv), **3** (14.1 mg, 53.2 µmol, 1.0 equiv), THF (1.5 mL) and water (9.6 µL, 532 µmol, 10.0 equiv) were used. The crude product was purified by silica-gel column chromatography (gradient of cyclohexane/dichloromethane from 2/1 to 1/1) and washed with methanol. The product was dried under high vacuum to give **7d** (15.2 mg, 12.9 µmol, 24%) as a white solid. m.p. > 350 °C. ^{1}H NMR (400 MHz, $CDCl_3$, 298 K): *δ* = 8.33 (s, 4H; Ar-*H*), 7.59–7.63 (m, 2H; Ar-*H*), 7.50–7.53 (m, 4H; Ar-*H*), 7.12 (d, *J* = 1.8 Hz, 8H; Ar-*H*), 7.04 (d, *J* = 1.8 Hz, 4H; Ar-*H*), 1.02 ppm (s, 72H; Alkyl-*H*). ^{13}C NMR (101 MHz, $CDCl_3$, 298 K): *δ* = 162.7, 151.9, 150.1, 142.5, 138.1, 131.5, 130.4, 129.8, 126.2, 123.0, 120.7, 120.4, 34.8, 31.3 ppm. UV/Vis ($CHCl_3$): λ_{max} (ε) = 381 nm (23200 M^{-1} cm^{-1}). MS (MALDI-TOF, pos. mode, DCTB in chloroform): *m/z*: 1170.7 [*M*]$^+$. HRMS (ESI-TOF, pos. mode, acetonitrile/chloroform 1/1): *m/z* calcd for $C_{82}H_{94}N_2O_4$+Na$^+$: 1193.7111 [*M*+Na]$^+$; found: 1193.7065.

_N_-[2,6-(Diphenyl)phenyl]perylene-3,4-dicarboximide (8a)

General method B: **1a** (50.0 mg, 204 µmol, 1.0 equiv), *n*-BuLi (255 µL, 1.6 M in *n*-hexane, 408 µmol, 2.0 equiv), **4** (75.1 mg, 204 µmol, 1.0 equiv) and THF (2.9 mL) were used. The crude product was purified by silica-gel column chromatography (gradient of cyclohexane/dichloromethane from 1/1 to 1/5) and washed with *n*-hexane and methanol. The product was dried under high vacuum to give **8a** (47.6 mg, 86.6 µmol, 42%) as a red solid. m.p. > 350 °C. ^{1}H NMR (400 MHz, $CDCl_3$, 298 K): *δ* = 8.38 (d, *J* = 8.1 Hz, 2H; Ar-*H*), 8.32 (d, *J* = 7.7 Hz, 2H; Ar-*H*), 8.25 (d, *J* = 8.1 Hz, 2H; Ar-*H*), 7.83 (d, *J* = 8.1 Hz, 2H; Ar-*H*), 7.53–7.63 (m, 3H; Ar-*H*), 7.48–7.52 (m, 2H; Ar-*H*), 7.36–7.40 (m, 4H; Ar-*H*), 7.13–7.19 (m, 4H; Ar-*H*), 7.06–7.11 ppm (m, 2H; Ar-*H*). ^{13}C NMR (101 MHz, $CDCl_3$, 298 K): *δ* = 164.1, 141.9, 139.5, 137.4, 134.3, 131.73, 131.71, 130.1, 130.3, 130.1, 129.2, 129.0, 128.5, 128.0, 127.9, 127.4, 127.1, 126.8, 123.8, 120.5, 120.1 ppm. UV/Vis ($CHCl_3$): λ_{max} (ε) / = 510 (29600 M^{-1} cm^{-1}). fluorescence ($CHCl_3$): $\lambda_{em,max}$ (ϕ_{fl}) = 538 nm (87±4%). MS (MALDI-TOF, pos. mode, DCTB in chloroform): *m/z*: 549.1 [*M*]$^+$. HRMS (ESI-TOF, pos. mode, acetonitrile/chloroform 1/1): *m/z* calcd for $C_{40}H_{23}NO_2$+Na$^+$: 572.1626 [*M*+Na]$^+$; found: 572.1604.

***N*-[2,6-bis(phenylethynyl)phenyl]perylene-3,4-dicarboximide (8b)**

General method B: **1b** (50.0 mg, 170 µmol, 1.0 equiv), *n*-BuLi (213 µL, 1.6 M in *n*-hexane, 341 µmol, 2.0 equiv), **4** (62.8 mg, 170 µmol, 1.0 equiv) and THF (2.4 mL) were used. The crude product was purified by silica-gel column chromatography (gradient of cyclohexane/dichloromethane from 1/1 to 1/2) and washed with *n*-hexane and methanol. The product was dried under high vacuum to give **8b** (24.0 mg, 40.2 µmol, 24%) as a red solid. m.p. 295–297 °C. ^{1}H NMR (400 MHz, $CDCl_3$, 298 K): δ = 8.71 (d, *J* = 8.1 Hz, 2H; Ar-*H*), 8.45–8.49 (m, 4H; *Ar*-H), 7.91–7.94 (m, 2H; *Ar*-H), 7.73 (d, *J* = 7.7 Hz, 2H; Ar-*H*), 7.65 (t, *J* = 7.7 Hz, 2H; Ar-*H*), 7.47–7.51 (m, 1H; *Ar*-H), 7.02–7.13 ppm (m, 10H; *Ar*-H). ^{13}C NMR (101 MHz, $CDCl_3$, 298 K): δ = 163.4, 139.5, 137.8, 134.5, 132.6, 132.2, 131.7, 131.2, 130.6, 129.4, 128.9, 128.5, 128.2, 128.2, 127.3, 127.2, 124.12, 124.06, 122.8, 121.1, 120.5, 94.1, 85.3 ppm. UV/Vis ($CHCl_3$): λ_{max} (ε) / = 510 nm (30900 M^{-1} cm^{-1}). fluorescence ($CHCl_3$): $\lambda_{em,max}$ (ϕ_{fl}) = 540 nm (86±4%). MS (MALDI-TOF, pos. mode, DCTB in chloroform): *m/z*: 597.2 [*M*]$^+$. HRMS (ESI-TOF, pos. mode, acetonitrile/chloroform 1/1): *m/z* calcd for $C_{44}H_{23}NO_2$+Na^+: 620.1626 [*M*+Na]$^+$; found: 620.1623.

***N*-[2,6-bis(4-*tert*-butylphenyl)phenyl]perylene-3,4-dicarboximide (8c)**

General method B: **1c** (50.0 mg, 140 µmol, 1.0 equiv), *n*-BuLi (175 µL, 1.6 M in *n*-hexane, 280 µmol, 2.0 equiv), **4** (51.5 mg, 140 µmol, 1.0 equiv) and THF (2.0 mL) were used. The crude product was purified by silica-gel column chromatography (gradient of cyclohexane/dichloromethane from 2/1 to 1/1) and washed with methanol. The product was dried under high vacuum to give **8c** (37.3 mg, 56.3 µmol, 40%) as a red solid. m.p. >350 °C. ^{1}H NMR (400 MHz, $CDCl_3$, 298 K): δ = 8.38 (d, *J* = 8.1 Hz, 2H; Ar-*H*), 8.34 (d, *J* = 7.6 Hz, 2H; Ar-*H*), 8.26 (d, *J* = 8.1 Hz, 2H; Ar-*H*), 7.82–7.85 (m, 2H; *Ar*-H), 7.54–7.60 (m, 3H; *Ar*-H), 7.47–7.50 (m, 2H; *Ar*-H), 7.28–7.32 (m, 4H; *Ar*-H), 7.13–7.17 (m, 4H; *Ar*-H), 1.08 ppm (s, 18H; *Ar*-H). ^{13}C NMR (101 MHz, $CDCl_3$, 298 K): δ = 164.2, 150.0, 141.8, 137.0, 136.7, 134.1, 131.9, 131.5, 130.8, 130.3, 130.0, 129.0, 128.9, 128.2, 127.8, 126.9, 126.6, 125.0, 123.6, 120.6, 119.8, 34.5, 31.3 ppm. UV/Vis ($CHCl_3$): λ_{max} (ε) = 509 nm (31900 M^{-1} cm^{-1}). fluorescence ($CHCl_3$): $\lambda_{em,max}$ (ϕ_{fl}) = 539 nm (84±4%). MS (MALDI-TOF, pos. mode, DCTB in chloroform): *m/z*: 661.3 [*M*]$^+$. HRMS (ESI-TOF, pos. mode, acetonitrile/chloroform 1/1): *m/z* calcd for $C_{48}H_{39}NO_2$+Na^+: 684.2878 [*M*+Na]$^+$; found: 684.2839.

***N*-[2,6-bis(3,5-di-*tert*-butylphenyl)phenyl]perylene-3,4-dicarboximide (8d)**

General method B: **1d** (50.0 mg, 106 µmol, 1.0 equiv), *n*-BuLi (133 µL, 1.6 M in *n*-hexane, 212 µmol, 2.0 equiv), **4** (39.2 mg, 106 µmol, 1.0 equiv) and THF (1.5 mL) were used. The crude product was purified by silica-gel column chromatography (gradient of cyclohexane/dichloromethane from 2/1 to 1/2) and washed with methanol. The product was dried under high vacuum to give **8d** (18.8 mg, 24.3 µmol, 23%) as a red solid. m.p. 316–318 °C. ^{1}H NMR (400 MHz, $CDCl_3$, 298 K): δ = 8.38 (d, *J* = 7.8 Hz, 2H; Ar-*H*), 8.35 (d, *J* = 8.1 Hz, 2H; Ar-*H*), 8.28 (d, *J* = 8.1 Hz, 2H; Ar-*H*), 7.87 (d, *J* = 8.1 Hz, 2H; Ar-*H*), 7.57–7.62 (m, 3H; Ar-*H*), 7.52–7.55 (m, 2H; Ar-*H*), 7.24 (d, *J* = 1.9 Hz, 4H; Ar-*H*), 7.07(t, *J* = 1.9 Hz, 2H; Ar-*H*), 1.07 ppm (s, 36H; *Ar*-H). ^{13}C NMR (101 MHz, $CDCl_3$, 298 K): δ = 163.8, 149.9, 142.7, 138.5, 136.9, 134.4, 132.5, 131.3, 130.8, 130.0, 129.6, 129.4, 128.9, 128.1, 127.1, 126.7, 123.7, 123.2, 121.1, 120.7, 120.0, 34.8, 31.3 ppm. UV/Vis ($CHCl_3$): λ_{max} (ε) = 508 nm (31500 M^{-1} cm^{-1}). fluorescence ($CHCl_3$): $\lambda_{em,max}$ (ϕ_{fl}) = 534 nm (84±4%). MS (MALDI-TOF, pos. mode, DCTB in chloroform): *m/z*: 773.4 [*M*]$^+$. HRMS (ESI-TOF, pos. mode, acetonitrile/chloroform 1/1): *m/z* calcd for $C_{56}H_{55}NO_2$+Na^+: 796.4130 [*M*+Na]$^+$; found: 796.4103.

***N,N*`-Bis[2,6-(diphenyl)phenyl]perylene-3,4:9,10-bis(dicarboximide) (9a)**

General method B: **1a** (50.0 mg, 204 µmol, 2.0 equiv), *n*-BuLi (255 µL, 1.6 M in *n*-hexane, 408 µmol, 4.0 equiv), **5** (49.4 mg, 102 µmol, 1.0 equiv) and THF (2.9 mL) were used. The crude product was purified by silica-gel column chromatography (gradient of cyclohexane/dichloromethane from 3/1 to 1/3) and washed with *n*-hexane and methanol. The product was dried under high vacuum to give **9a** (16.5 mg, 19.5 µmol, 19%) as a red solid. m.p. >350 °C. ^{1}H NMR (400 MHz, CD_2Cl_2, 298 K): δ =8.37 (d, *J* = 8.1 Hz, 4H; Ar-*H*), 8.17–8.25 (br, 4H; Ar-*H*), 7.61–7.65 (m, 2H; Ar-*H*), 7.49–7.52 (m, 4H; Ar-*H*), 7.33–7.38 (m, 8H; Ar-*H*), 7.13–7.19 (m, 8H; Ar-*H*), 7.06–7.11 ppm (m, 4H; Ar-*H*). ^{13}C NMR (101 MHz, $CDCl_3$, 298 K): δ = 163.4, 141.8, 139.5, 134.3, 131.4, 131.2, 130.4, 129.3, 129.2, 128.6, 128.1, 127.5, 126.1, 122.6, 122.5 ppm. UV/Vis ($CHCl_3$): λ_{max} (ε) = 528 nm (77400 M^{-1} cm^{-1}). fluorescence ($CHCl_3$): $\lambda_{em,max}$ (ϕ_{fl}) = 535 nm (95±2%). MS (MALDI-TOF, pos. mode, DCTB in chloroform): *m/z*: 846.2 [*M*]$^+$. HRMS (ESI-TOF, pos. mode, acetonitrile/chloroform 1/1): *m/z* calcd for $C_{60}H_{34}N_2O_4$+Na^+: 869.2416 [*M*+Na]$^+$; found: 869.2484.

***N,N*`-Bis[2,6-di(phenylethynyl)phenyl]perylene-3,4:9,10-bis(dicarboximide) (9b)**

General method B: **1b** (50.0 mg, 170 µmol, 2.0 equiv), *n*-BuLi (213 µL, 1.6 M in *n*-hexane, 341 µmol, 4.0 equiv), **5** (41.3 mg, 85.2 µmol, 1.0 equiv) and THF (2.4 mL) were used. The crude product was purified by silica-gel column chromatography (gradient of cyclohexane/dichloromethane from 1/2 to 0/1) and washed with *n*-hexane and methanol. The product was dried under high vacuum to give **9b** (9.7 mg, 10.3 µmol, 12%) as a red solid. m.p. >350 °C. ^{1}H NMR (400 MHz, $CDCl_3$, 298 K): δ = 8.84 (d, *J* = 8.0 Hz, 4H; Ar-*H*), 8.74 (d, *J* = 8.0 Hz, 4H; Ar-*H*), 7.75 (d, *J* = 7.9 Hz, 4H; Ar-*H*), 7.50–7.55 (m, 2H; Ar-*H*), 7.04–7.16 ppm (m, 20H; Ar-*H*). ^{13}C NMR (101 MHz, $CDCl_3$, 298 K): δ = 162.8, 138.8, 135.3, 132.8, 132.1, 131.6, 130.1, 129.2, 128.6, 128.3, 127.0, 124.0, 123.6, 123.5, 122.6, 94.2, 85.0 ppm. UV/Vis ($CHCl_3$): λ_{max} (ε) = 527 nm (78800 M^{-1} cm^{-1}). fluorescence ($CHCl_3$): $\lambda_{em,max}$ (ϕ_{fl}) = 534 nm (96±1%). MS (MALDI-TOF, pos. mode, DCTB in chloroform): *m/z*: 942.3 [*M*]$^+$. HRMS (ESI-TOF, pos. mode, acetonitrile/chloroform 1/1): *m/z* calcd for $C_{68}H_{34}N_2O_4$+Na^+: 965.2416 [*M*+Na]$^+$; found: 965.2419.

***N,N*`-Bis[2,6-bis(4-*tert*-butylphenyl)phenyl]perylene-3,4:9,10-bis(dicarboximide) (9c)**

General method B: **1c** (50.0 mg, 140 µmol, 2.0 equiv), *n*-BuLi (175 µL, 1.6 M in *n*-hexane, 280 µmol, 4.0 equiv), **5** (33.9 mg, 69.9 µmol, 1.0 equiv) and THF (2.0 mL) were used. The crude product was purified by silica-gel column chromatography (gradient of cyclohexane/dichloromethane from 1/1 to 1/5) and washed with *n*-hexane and methanol. The product was dried under high vacuum to give **9c** (15.6 mg, 14.6 µmol, 21%) as a red solid. m.p. >350 °C. ^{1}H NMR (400 MHz, $C_2D_2Cl_4$,

373 K): δ = 8.43–8.18 (br, 8H; *Ar*-H, due to broad signal not all protons could be resolved), 7.51–7.56 (m, 2H; Ar-*H*), 7.44–7.47 (m, 4H; Ar-*H*), 7.23 (d, *J* = 8.3 Hz, 8H; Ar-*H*), 7.08 (d, *J* = 8.3 Hz, 8H; Ar-*H*), 1.03 ppm (s, 36H; Ar-*H*). ^{13}C NMR (101 MHz, $C_2D_2Cl_4$, 373 K): δ = 163.4, 150.4, 142.1, 136.7, 132.2, 131.1, 130.2, 129.4, 128.91, 128.86, 128.6, 124.7, 123.4, 122.6, 120.6, 34.4, 31.3 ppm. UV/Vis ($CHCl_3$): λ_{max} (ε) = 528 nm (87700 M^{-1} cm^{-1}). fluorescence ($CHCl_3$): $\lambda_{em,max}$ (ϕ_{fl}) = 533 nm (97±1%). MS (MALDI-TOF, pos. mode, DCTB in chloroform): *m*/*z*: 1070.4 [*M*]$^+$. HRMS (ESI-TOF, pos. mode, acetonitrile/chloroform 1/1): *m*/*z* calcd for $C_{76}H_{66}N_2O_4$+Na^+: 1093.4920 [*M*+Na]$^+$; found: 1093.4865.

_N,N_`-Bis[2,6-bis(3,5-di-_tert_-butylphenyl)phenyl]perylene-3,4:9,10-bis(dicarboximide) (9d)

General method B: **1d** (50.0 mg, 106 µmol, 2.0 equiv), *n*-BuLi (133 µL, 1.6 M in *n*-hexane, 212 µmol, 4.0 equiv), **5** (25.8 mg, 53.2 µmol, 1.0 equiv) and THF (1.5 mL) were used. The crude product was purified by silica-gel column chromatography (gradient of cyclohexane/dichloromethane from 2/1 to 1/2) and washed with methanol. The product was dried under high vacuum to give **9d** (14.1 mg, 10.8 µmol, 18%) as a red-orange solid. m.p. >350 °C. ^{1}H NMR (400 MHz, $CDCl_3$, 298 K): δ = 8.40 (s, 8H; Ar-*H*), 7.59–7.64 (m, 2H; Ar-*H*), 7.52–7.55 (m, 4H; Ar-*H*), 7.20 (d, *J* = 1.9 Hz, 8H; Ar-*H*), 7.06 (t, *J* = 1.9 Hz, 4H; Ar-*H*), 1.06 ppm (s, 72H; Ar-*H*). ^{13}C NMR (101 MHz, $CDCl_3$, 298 K): δ = 163.2, 149.9, 142.6, 138.4, 134.6, 132.0, 131.2, 129.7, 129.4, 129.1, 126.4, 123.2, 123.1, 122.8, 120.7, 34.8, 31.4 ppm. UV/Vis ($CHCl_3$): λ_{max} (ε) = 527 nm (91100 M^{-1} cm^{-1}). fluorescence ($CHCl_3$): $\lambda_{em,max}$ (ϕ_{fl}) = 534 nm (93±2%).MS (MALDI-TOF, pos. mode, DCTB in chloroform): *m*/*z*: 1294.6 [*M*]$^+$. HRMS (ESI-TOF, pos. mode, acetonitrile/chloroform 1/1): *m*/*z* calcd for $C_{92}H_{98}N_2O_4$+Na^+: 1317.7424 [*M*+Na]$^+$; found: 1317.7379.

3-[2,6-(Diphenyl)phenyl]imino-_1H_,_3H_-Naphtho[1,8-_cd_]pyran-1-one (10a)

In a Schlenk-tube **1a** (50.0 mg, 204 µmol, 1.0 equiv) was dissolved in THF (1.5 mL) under a nitrogen atmosphere and cooled to –78 °C. Subsequently, LDA (102 µL, 2.0 M in THF/heptane/ethylbenzene, 204 µmol, 1.0 equiv) was added and the reaction mixture was stirred for 1 h at –78 °C. The solution was allowed to warm up to room temperature and **3** (40.4 mg, 204 µmol, 1.0 equiv) was added. The mixture was stirred at 75 °C for 6 h and then cooled to room temperature. The resulting mixture was concentrated under reduced pressure. The crude product was purified by silica-gel column chromatography (gradient of cyclohexane/dichloromethane from 1/1 to 1/3) and washed with cold methanol. The product was dried under high vacuum to give **10a** (10.9 mg, 25.6 µmol, 13%) as a yellow solid. m.p. 249–251 °C. ^{1}H NMR (400 MHz, $CDCl_3$, 298 K): δ = 8.48 (dd, *J* = 1.0 Hz, *J* = 7.4 Hz, 1H; *Ar*-H), 8.30 (dd, *J* = 1.2 Hz, *J* = 7.4 Hz, 1H; *Ar*-H), 8.14 (dd, *J* = 1.1 Hz, *J* = 8.3 Hz, 1H; *Ar*-H), 8.05 (dd, *J* = 1.0 Hz, *J* = 8.3 Hz, 1H; *Ar*-H), 7.61–7.67 (m, 2H; *Ar*-H), 7.51–7.55 (m, 4H; *Ar*-H), 7.39–7.42 (m, 2H; *Ar*-H), 7.30–7.34 (m, 1H; *Ar*-H), 7.19–7.25 (m, 4H; *Ar*-H), 7.08–7.13 ppm (m, 2H; *Ar*-H). ^{13}C NMR (101 MHz, $CDCl_3$, 298 K): δ = 159.4, 146.3, 141.3, 140.6, 134.6, 133.8, 131.8, 131.7, 131.6, 129.7, 129.4, 129.3, 129.0, 127.9, 127.5, 126.7, 126.7, 124.7, 121.4, 118.7 ppm. MS (MALDI-TOF, pos. mode, DCTB in chloroform): *m*/*z*: 425.1 [*M*]$^+$. HRMS (ESI-TOF, pos. mode, acetonitrile/chloroform 1/1): *m*/*z* calcd for $C_{30}H_{19}NO_2$+H^+: 426.1494 [*M*+H]$^+$; found: 426.1490.

Conversion of isoimide 10a to imide 6a

In a Schlenk-tube **10a** (10.0 mg, 23.5 µmol, 1.0 equiv) was dissolved in THF (1.0 mL) under a nitrogen atmosphere and heated to 75 °C. Subsequently, *tert*-BuOK solution (0.24 mL, 0.5 M in water, 118 µmol, 5.0 equiv) was carefully added and heated for 6 h at 75 °C. The reaction mixture was cooled to room temperature and 20 mL dichloromethane and 20 mL water was added. The mixture was extracted with dichloromethane. The combined organic layers were washed with water, dried over $MgSO_4$ and concentrated under reduced pressure. The product was dried under high vacuum to give **6a** (9.9 mg, 23.5 µmol, 99%) as a white solid. ^{1}H NMR spectrum was in accordance with product identity of one-pot procedure.

Single crystal X-ray analysis

Crystals suitable for single x-ray diffraction were grown by slow evaporation of concentrated dichloromethane or chloroform solutions or slow diffusion of *n*-hexane or methanol into dichloromethane or chloroform solutions.

Table S3. Crystal data and structure refinement for **6a**.

CCDC Number	1988196
Empirical formula	$C_{30}H_{19}NO_2$, 0.237(CH_4O)
Formula weight	433.07
Temperature	100(2) K
Wavelength	1.54178 Å
Crystal system, space group	Monoclinic, $P2_1/n$
Unit cell dimensions	a = 9.4521(3) Å
	b = 27.4078(8) Å
	c = 17.1937(5) Å
	α = 90 °
	β = 92.3620(10) °
	γ = 90 °
Volume	4450.4(2) Å^3
Z	8
Calculated density	1.293 Mg/m^3
Absorption coefficient	0.644 mm^{-1}
F(000)	1810
Crystal size	0.520 × 0.490 × 0.470 mm
Theta range for data collection	3.036 to 72.417 °
Limiting indices	–11 <= h <= 11, –32 <= k <= 33, –21 <=l <= 21
Reflections collected / unique	93592 / 8779 [R_{int} = 0.0271]
Completeness	99.7%
Absorption correction	Semi-empirical from equivalents
Max. and min. transmission	0.7536 and 0.6771
Refinement method	Full-matrix least-squares on F^2
Data / restraints / parameters	8779 / 1 / 635
Goodness-of-fit	1.037
Final R indices [$I > 2\sigma(I)$]	R_1 = 0.0421, wR_2 = 0.1068
R indices (all data)	R_1 = 0.0441, wR_2 = 0.1085
Largest diff. peak and hole	0.526 and –0.349 e·Å^{-3}

Table S4. Crystal data and structure refinement for **6b**.

CCDC Number	1988190
Empirical formula	$C_{34}H_{19}NO_2$
Formula weight	473.50
Temperature	100(2) K
Wavelength	1.54178 Å
Crystal system, space group	Triclinic, $P\bar{1}$
Unit cell dimensions	a = 9.5047(4) Å
	b = 11.4030(5) Å
	c = 13.0745(5) Å
	α = 113.5620(10) °
	β = 96.4210(10) °
	γ = 106.8600(10) °
Volume	1200.43(9) $Å^3$
Z	2
Calculated density	1.310 Mg/m^3
Absorption coefficient	0.641 mm^{-1}
F(000)	492
Crystal size	0.580 × 0.520 × 0.520 mm
Theta range for data collection	3.819 to 72.165 °
Limiting indices	$-11 <= h <= 11$, $-14 <= k <= 14$, $-16 <= l <= 16$
Reflections collected / unique	20993 / 4697 [R_{int} = 0.0352]
Completeness	99.7%
Absorption correction	Semi-empirical from equivalents
Max. and min. transmission	0.7536 and 0.5996
Refinement method	Full-matrix least-squares on F^2
Data / restraints / parameters	4697 / 0 / 335
Goodness-of-fit on F^2	1.078
Final R indices [$I > 2\sigma(I)$]	R_1 = 0.0384, wR_2 = 0.0959
R indices (all data)	R_1 = 0.0417, wR_2 = 0.0983
Largest diff. peak and hole	0.270 and –0.296 $e \cdot Å^{-3}$

Table S5. Crystal data and structure refinement for **6c**.

CCDC Number	1988198
Empirical formula	$C_{38}H_{35}NO_2$, 0.276(C_6H_{14})
Formula weight	561.44
Temperature	100(2) K
Wavelength	1.54178 Å
Crystal system, space group	Trigonal, $R\overline{3}$
Unit cell dimensions	a = 42.7740(12) Å
	b = 42.774(3) Å
	c = 9.467(5) Å
	a = 90 °
	β = 90 °
	γ = 120 °
Volume	15000(8) Å^3
Z	18
Calculated density	1.119 Mg/m^3
Absorption coefficient	0.525 mm^{-1}
F(000)	5396
Crystal size	0.391 × 0.244 × 0.236 mm
Theta range for data collection	3.579 to 72.359 °
Limiting indices	–52 <= *h* <= 50, –52 <= *k* <= 52, –11 <=*l* <= 11
Reflections collected / unique	128131 / 6597 [R_{int} = 0.0492]
Completeness	100.0%
Absorption correction	Semi-empirical from equivalents
Max. and min. transmission	0.7536 and 0.6340
Refinement method	Full-matrix least-squares on F^2
Data / restraints / parameters	6597 / 0 / 384
Goodness-of-fit	1.057
Final *R* indices [$I > 2\sigma(I)$]	R_1 = 0.0644, wR_2 = 0.1700
R indices (all data)	R_1 = 0.0669, wR_2 = 0.1722
Largest diff. peak and hole	0.704 and –0.357 e·Å^{-3}

Table S6. Crystal data and structure refinement for **6d**.

CCDC Number	1988197
Empirical formula	$C_{46}H_{51}NO_2$
Formula weight	649.88
Temperature	100(2) K
Wavelength	1.54178 Å
Crystal system, space group	Monoclinic, $P2_1/n$
Unit cell dimensions	a = 9.3190(6) Å
	b = 23.5604(14) Å
	c = 17.6446(11) Å
	a = 90 °
	β = 97.518(3) °
	γ = 90 °
Volume	3840.7(4) $Å^3$
Z	4
Calculated density	1.124 Mg/m^3
Absorption coefficient	0.516 mm^{-1}
F(000)	1400
Crystal size	0.226 × 0.110 × 0.078 mm
Theta range for data collection	3.146 to 72.674 °
Limiting indices	–11 <= *h* <= 11, –29 <= *k* <= 29, –21 <=*l* <= 21
Reflections collected / unique	69915 / 7613 [R_{int} = 0.0565]
Completeness	100.0%
Absorption correction	Semi-empirical from equivalents
Max. and min. transmission	0.7536 and 0.6670
Refinement method	Full-matrix least-squares on F^2
Data / restraints / parameters	7613 / 0 / 454
Goodness-of-fit	1.036
Final *R* indices [$I > 2\sigma(I)$]	R_1 = 0.0498, wR_2 = 0.1250
R indices (all data)	R_1 = 0.0581, wR_2 = 0.1317
Largest diff. peak and hole	0.599 and –0.284 e·$Å^{-3}$

Table S7. Crystal data and structure refinement for **8a**.

CCDC Number	1988191
Empirical formula	$C_{40}H_{23}NO_2$
Formula weight	549.59
Temperature	100(2) K
Wavelength	1.54178 Å
Crystal system, space group	Monoclinic, $P2_1/n$
Unit cell dimensions	a = 8.2649(8) Å
	b = 18.9881(18) Å
	c = 16.9669(16) Å
	a = 90 °
	β = 94.814(4) °
	γ = 90 °
Volume	2653.3(4) $Å^3$
Z	4
Calculated density	1.376 Mg/m^3
Absorption coefficient	0.662 mm^{-1}
F(000)	1144
Crystal size	0.691 × 0.328 × 0.248 mm
Theta range for data collection	3.500 to 72.820 °
Limiting indices	$-10 <= h <= 10, -19 <= k <= 23, -20 <= l <= 20$
Reflections collected / unique	46803 / 5254 [R_{int} = 0.0530]
Completeness	100.0%
Absorption correction	Semi-empirical from equivalents
Max. and min. transmission	0.7536 and 0.6546
Refinement method	Full-matrix least-squares on F^2
Data / restraints / parameters	5254 / 0 / 388
Goodness-of-fit	1.050
Final R indices [$I > 2\sigma(I)$]	R_1 = 0.0382, wR_2 = 0.0953
R indices (all data)	R_1 = 0.0453, wR_2 = 0.1005
Largest diff. peak and hole	0.199 and –0.252 e·$Å^{-3}$

Table S8. Crystal data and structure refinement for **8d**.

CCDC Number	1988194
Empirical formula	$C_{56}H_{55}NO_2$, 2($CHCl_3$)
Formula weight	1012.74
Temperature	100(2) K
Wavelength	1.54178 Å
Crystal system, space group	Monoclinic, $P2_1/c$
Unit cell dimensions	a = 12.3323(3) Å
	b = 16.5743(4) Å
	c = 25.3982(6) Å
	a = 90 °
	β = 92.2790(10) °
	γ = 90 °
Volume	5187.3(2) Å ^{3}
Z	4
Calculated density	1.297 Mg/m^3
Absorption coefficient	3.352 mm^{-1}
F(000)	2120
Crystal size	0.485 × 0.358 × 0.142 mm
Theta range for data collection	3.185 to 72.222 °
Limiting indices	$-15 <= h <= 15$, $-19 <= k <= 20$, $-31 <= l <= 31$
Reflections collected / unique	69426 / 10235 [R_{int} = 0.0438]
Completeness	100.0%
Absorption correction	Semi-empirical from equivalents
Max. and min. transmission	0.7536 and 0.4999
Refinement method	Full-matrix least-squares on F^2
Data / restraints / parameters	10235 / 24 / 673
Goodness-of-fit	1.027
Final R indices [$I > 2\sigma(I)$]	R_1 = 0.0499, wR_2 = 0.1324
R indices (all data)	R_1 = 0.0574, wR_2 = 0.1378
Largest diff. peak and hole	0.530 and –0.889 e·Å $^{-3}$

Table S9. Crystal data and structure refinement for **9b**.

CCDC Number	1988192
Empirical formula	$C_{68}H_{34}N_2O_4$, 2(C_7H_8)
Formula weight	1127.24
Temperature	173(2) K
Wavelength	1.54178 Å
Crystal system, space group	Triclinic, $P\bar{1}$
Unit cell dimensions	*a* = 9.8083(3) Å
	b = 10.9966(3) Å
	c = 13.7096(4) Å
	a = 95.4630(10) °
	β = 96.3940(10) °
	γ = 91.7980(10) °
Volume	1461.50(7) $Å^3$
Z	1
Calculated density	1.281 Mg/m^3
Absorption coefficient	0.614 mm^{-1}
F(000)	588
Crystal size	0.271 × 0.133 × 0.068 mm
Theta range for data collection	3.260 to 72.215 °
Limiting indices	–12 <= *h* <= 12, –13 <= *k* <= 12, –16 <=*l* <= 16
Reflections collected / unique	28837 / 5734 [R_{int} = 0.0448]
Completeness	99.8%
Absorption correction	Semi-empirical from equivalents
Max. and min. transmission	0.7536 and 0.6827
Refinement method	Full-matrix least-squares on F^2
Data / restraints / parameters	5734 / 1 / 393
Goodness-of-fit	1.031
Final *R* indices [$I > 2\sigma(I)$]	R_1 = 0.0401, wR_2 = 0.1064
R indices (all data)	R_1 = 0.0495, wR_2 = 0.1130
Largest diff. peak and hole	0.186 and –0.208 e·$Å^{-3}$

Table S10. Crystal data and structure refinement for **9d**.

CCDC Number	1988195
Empirical formula	$C_{92}H_{98}N_2O_4$, (CH_2Cl_2) (CH_4O)
Formula weight	1412.69
Temperature	100(2) K
Wavelength	1.54178 Å
Crystal system, space group	Triclinic, $P\bar{1}$
Unit cell dimensions	*a* = 16.5016(7) Å
	b = 17.4206(8) Å
	c = 17.6995(9) Å
	a = 99.922(3) °
	β = 112.305(3) °
	γ = 112.149(2) °
Volume	4053.9(3) $Å^3$
Z	2
Calculated density	1.157 Mg/m^3
Absorption coefficient	1.128 mm^{-1}
F(000)	1512
Crystal size	0.122 × 0.074 × 0.059 mm
Theta range for data collection	2.902 to 72.314 °
Limiting indices	–20 <= *h* <= 20, –21 <= *k* <= 21, –17 <= *l* <= 21
Reflections collected / unique	80522 / 15965 [R_{int} = 0.0475]
Completeness	99.9%
Absorption correction	Semi-empirical from equivalents
Max. and min. transmission	0.7536 and 0.5884
Refinement method	Full-matrix least-squares on F^2
Data / restraints / parameters	15965 / 1 / 975
Goodness-of-fit	1.027
Final *R* indices [$I > 2\sigma(I)$]	R_1 = 0.0695, wR_2 = 0.1940
R indices (all data)	R_1 = 0.0855, wR_2 = 0.2117
Largest diff. peak and hole	0.922 and –1.101 $e{\cdot}Å^{-3}$

Table S11. Crystal data and structure refinement for **10a**.

CCDC Number	1988193
Empirical formula	$C_{30}H_{19}NO_2$
Formula weight	425.46
Temperature	100(2) K
Wavelength	1.54178 Å
Crystal system, space group	Orthorhombic, *Pbca*
Unit cell dimensions	a = 7.5150(7) Å
	b = 18.211(2) Å
	c = 31.154(3) Å
	a = 90 °
	β = 90 °
	γ = 90 °
Volume	4263.5(7) Å^3
Z	8
Calculated density	1.326 Mg/m^3
Absorption coefficient	0.655 mm^{-1}
F(000)	1776
Crystal size	0.202 × 0.088 × 0.024 mm
Theta range for data collection	2.837 to 72.481 °
Limiting indices	$-8 <= h <= 9$, $-22 <= k <= 22$, $-37 <= l <= 26$
Reflections collected / unique	26331 / 4217 [R_{int} = 0.0628]
Completeness	99.9%
Absorption correction	Semi-empirical from equivalents
Max. and min. transmission	0.7536 and 0.6494
Refinement method	Full-matrix least-squares on F^2
Data / restraints / parameters	4217 / 0 / 298
Goodness-of-fit	1.031
Final R indices [$I > 2\sigma(I)$]	R_1 = 0.0437, wR_2 = 0.1059
R indices (all data)	R_1 = 0.0585, wR_2 = 0.1164
Largest diff. peak and hole	0.186 and –0.205 e·Å^{-3}

(a) (b) (c)

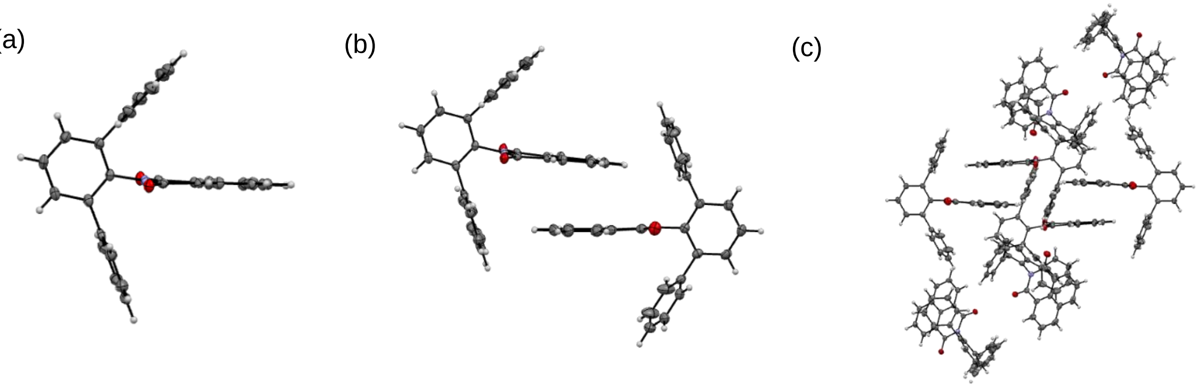

Figure S1. Molecular structure of **6a** in the solid-state. (a) Side view on the imide substituent, (b) dimeric fashion motif and (c) packing arrangement in the solid-state (methanol molecules were omitted for clarity.

(a) (b) (c)

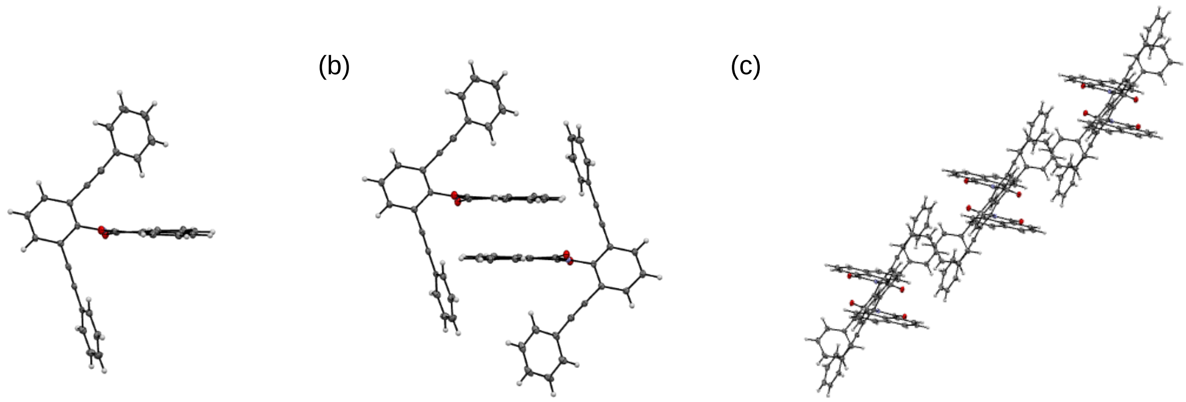

Figure S2. Molecular structure of **6b** in the solid-state. (a) Side view on the imide substituent, (b) dimeric fashion motif and (c) packing arrangement in the solid-state.

(a) (b) (c)

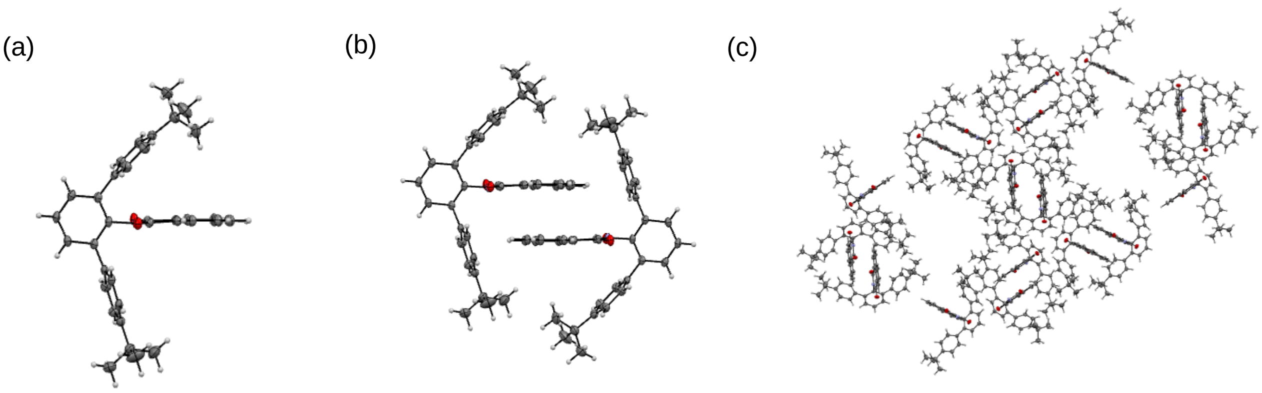

Figure S3. Molecular structure of **6c** in the solid-state. (a) Side view on the imide substituent, (b) dimeric fashion motif and (c) packing arrangement in the solid-state (*n*-hexane molecules were omitted for clarity).

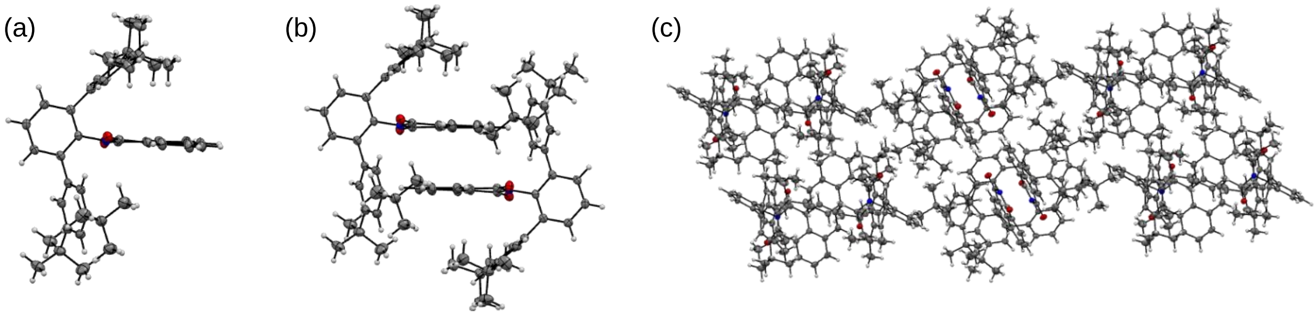

Figure S4. Molecular structure of **6d** in the solid-state. (a) Side view on the imide substituent, (b) dimeric fashion motif and (c) packing arrangement in the solid-state.

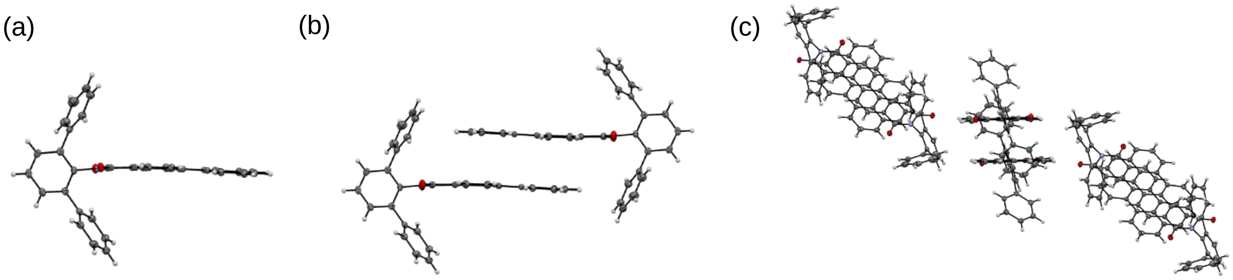

Figure S5. Molecular structure of **8a** in the solid-state. (a) Side view on the imide substituent, (b) dimeric fashion motif and (c) packing arrangement in the solid-state.

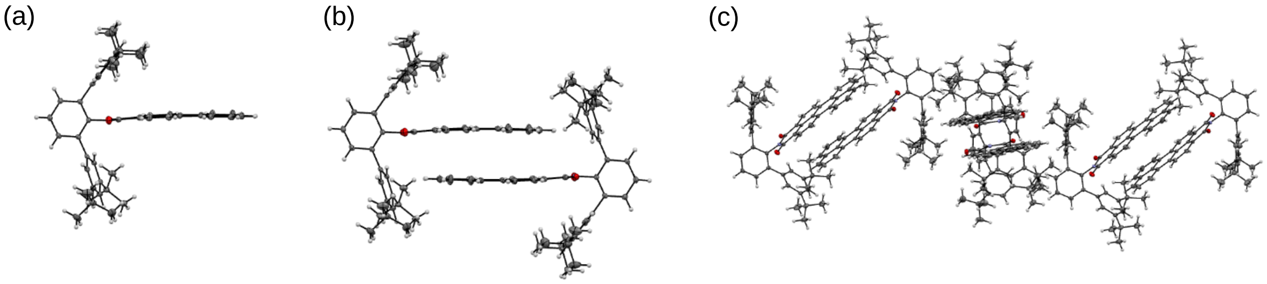

Figure S6. Molecular structure of **8d** in the solid-state. (a) Side view on the imide substituent, (b) dimeric fashion motif and (c) packing arrangement in the solid-state (chloroform molecules were omitted for clarity).

(a) (b) (c)

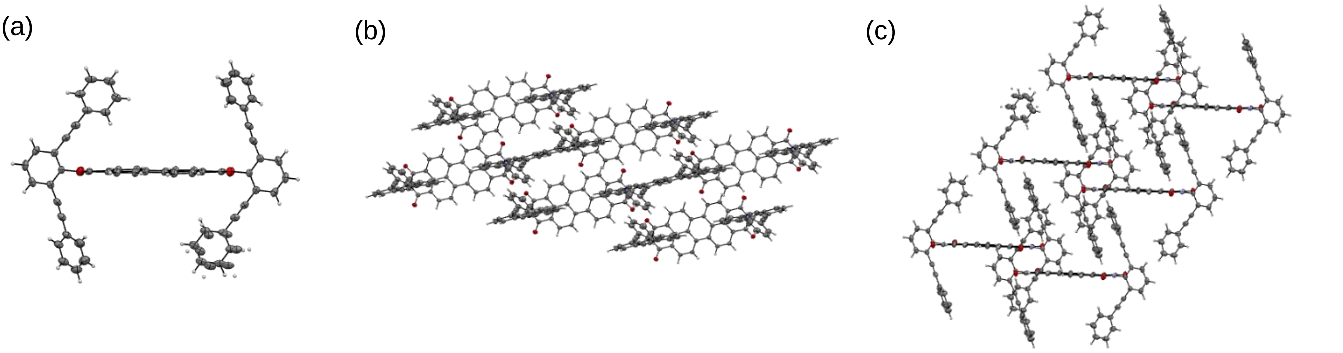

Figure S7. Molecular structure of **9b** in the solid-state. (a) Side view on the imide substituent, (b) and (c) packing arrangement in the solid-state (toluene molecules were omitted for clarity).

(a) (b) (c)

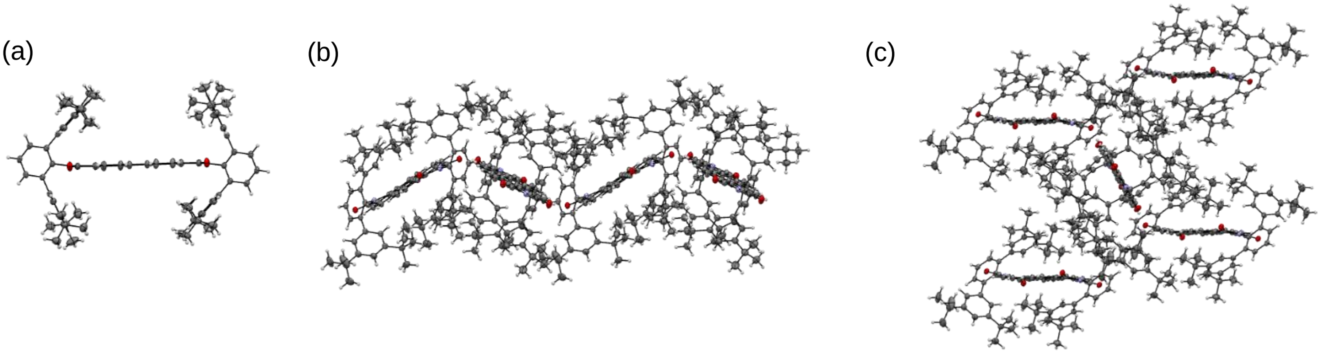

Figure S8. Molecular structure of **9d** in the solid-state. (a) Side view on the imide substituent, (b) and (c) packing arrangement in the solid-state (dichloromethane and methanol molecules were omitted for clarity).

(a) (b) (c)

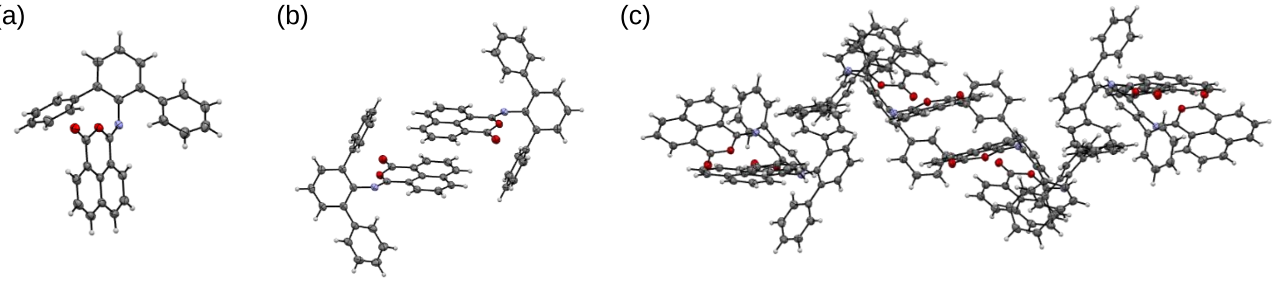

Figure S9. Molecular structure of **10a** in the solid-state. (a) Side view, (b) dimeric fashion motif and (c) packing arrangement in the solid-state.

Optical properties in solution

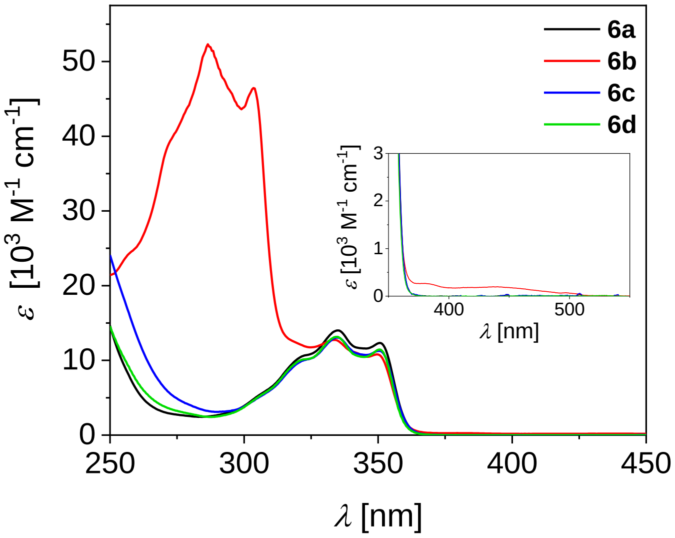

Figure S10. UV/Vis spectra of **6a**–**d** in chloroform solutions at room temperature (c ~ 10^{-5} M). Inset: Magnified region between 350-550 nm.

Table S11. UV/Vis absorption properties of naphthalene monoimides **6a**–**d** in chloroform solutions at room temperature.

	λ_{abs} [nm] (ε [M^{-1} cm^{-1}])
**6a**	351 (12300)
**6b**	350 (10800)
**6c**	351 (11300)
**6d**	351 (11400)

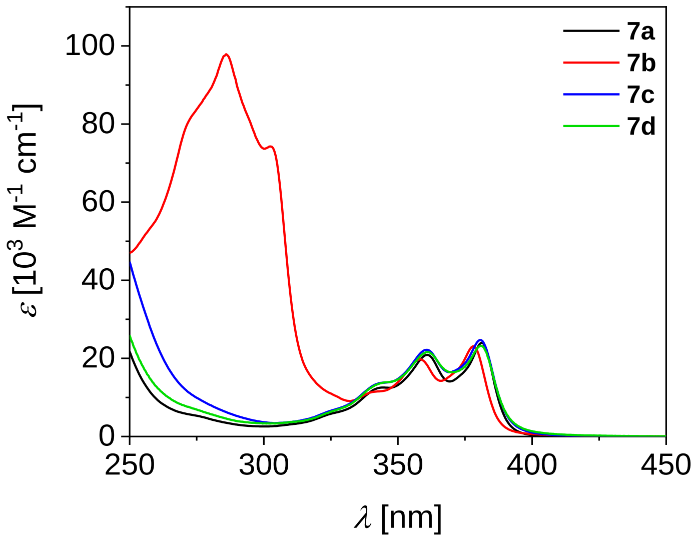

Figure S11. UV/Vis spectra of **7a**–**d** in chloroform solutions at room temperature (c ~ 10^{-5} M).

Table S12. UV/Vis absorption properties of naphthalene bisimides **7a**–**d** in chloroform solutions at room temperature.

	λ_{abs} [nm] (ε [M^{-1} cm^{-1}])
**7a**	381 (24000)
**7b**	378 (23100)
**7c**	381 (24700)
**7d**	381 (23200)

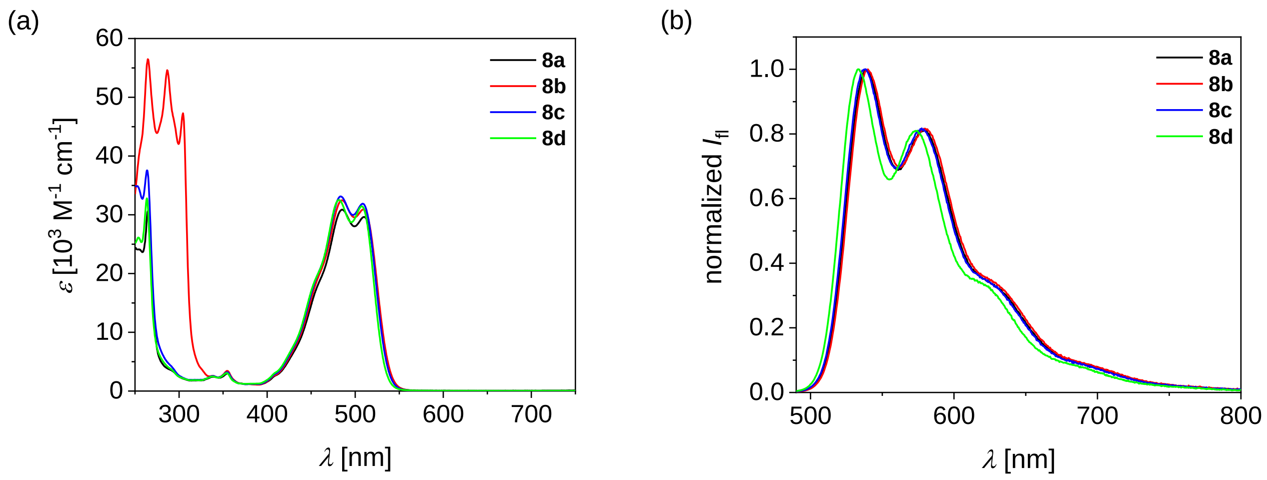

Figure S12. (a) UV/Vis and (b) fluorescence spectra and fluorescence of **8a**–**d** in chloroform solutions at room temperature (c ~ 10^{-5}–10^{-7}M).

Table S13. Optical properties of perylene monoimides **8a**–**d** in chloroform solutions at room temperature.

	λ_{abs} [nm] (ε [M^{-1} cm^{-1}])	λ_{em} [nm]	Stokes shift [cm^{-1}]	Φ_f [%][a]	τ [ns][b]
**8a**	510 (29600)	538	1020	87 ± 4	5.0
**8b**	510 (30900)	540	1090	86 ± 4	5.2
**8c**	509 (31900)	539	1090	84 ± 4	5.0
**8d**	508 (31500)	534	960	84 ± 4	4.8

[a] Fluorescence quantum yields were determined using the relative method (A < 0.05) and *N*,*N*'-bis(2,6-diisopropylphenyl)perylene-3,4:9,10-bis(dicarboximide) (Φ_f ($CHCl_3$) = 1.00) as reference. [b] excitation wavelength: λ_{ex} = 506 nm; detection wavelength: λ_{det} = 537 nm.

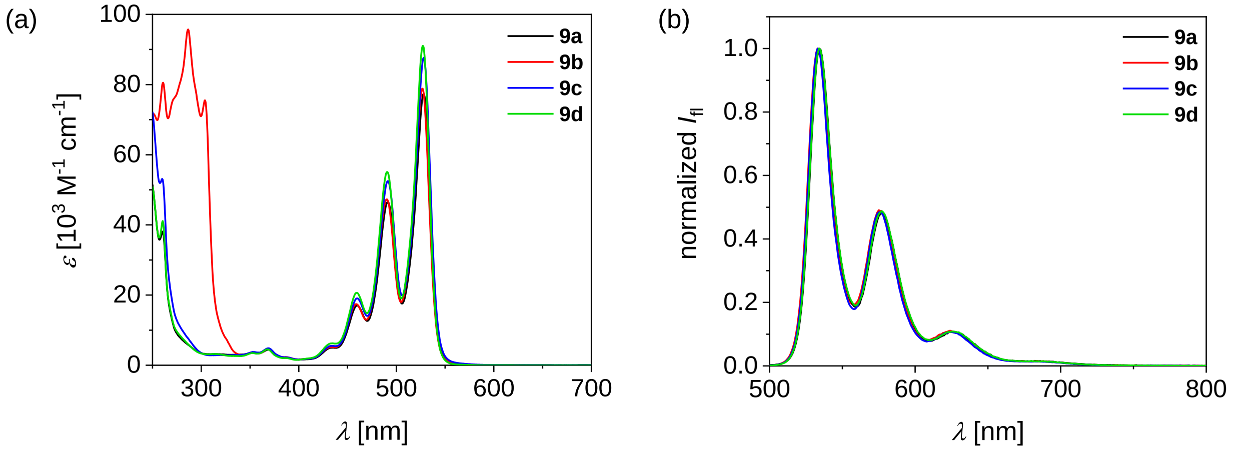

Figure S13. (a) UV/Vis and (b) fluorescence spectra of **9a**–**d** in chloroform solutions at room temperature (c ~ 10^{-5}–10^{-7}M).

Table S14. Optical properties of perylene bisimides **9a**–**d** in chloroform solutions at room temperature.

	λ_{abs} [nm] (ε [M^{-1} cm^{-1}])	λ_{em} [nm]	Stokes shift [cm^{-1}]	Φ_f [%][a]	τ [ns][b]
**9a**	528 (77400)	535	250	95 ± 2	3.8
**9b**	527 (78800)	534	250	96 ± 1	3.7
**9c**	528 (87700)	533	180	97 ± 1	3.8
**9d**	527 (91100)	534	250	93 ± 2	3.8

[a] Fluorescence quantum yields were determined using the relative method (A < 0.05) and *N*,*N'*-bis(2,6-diisopropylphenyl)perylene-3,4:9,10-bis(dicarboximide) (Φ_f ($CHCl_3$) = 1.00) as reference. [b] excitation wavelength: λ_{ex} = 506 nm; detection wavelength: λ_{det} = 537 nm.

Optical properties in the solid-state

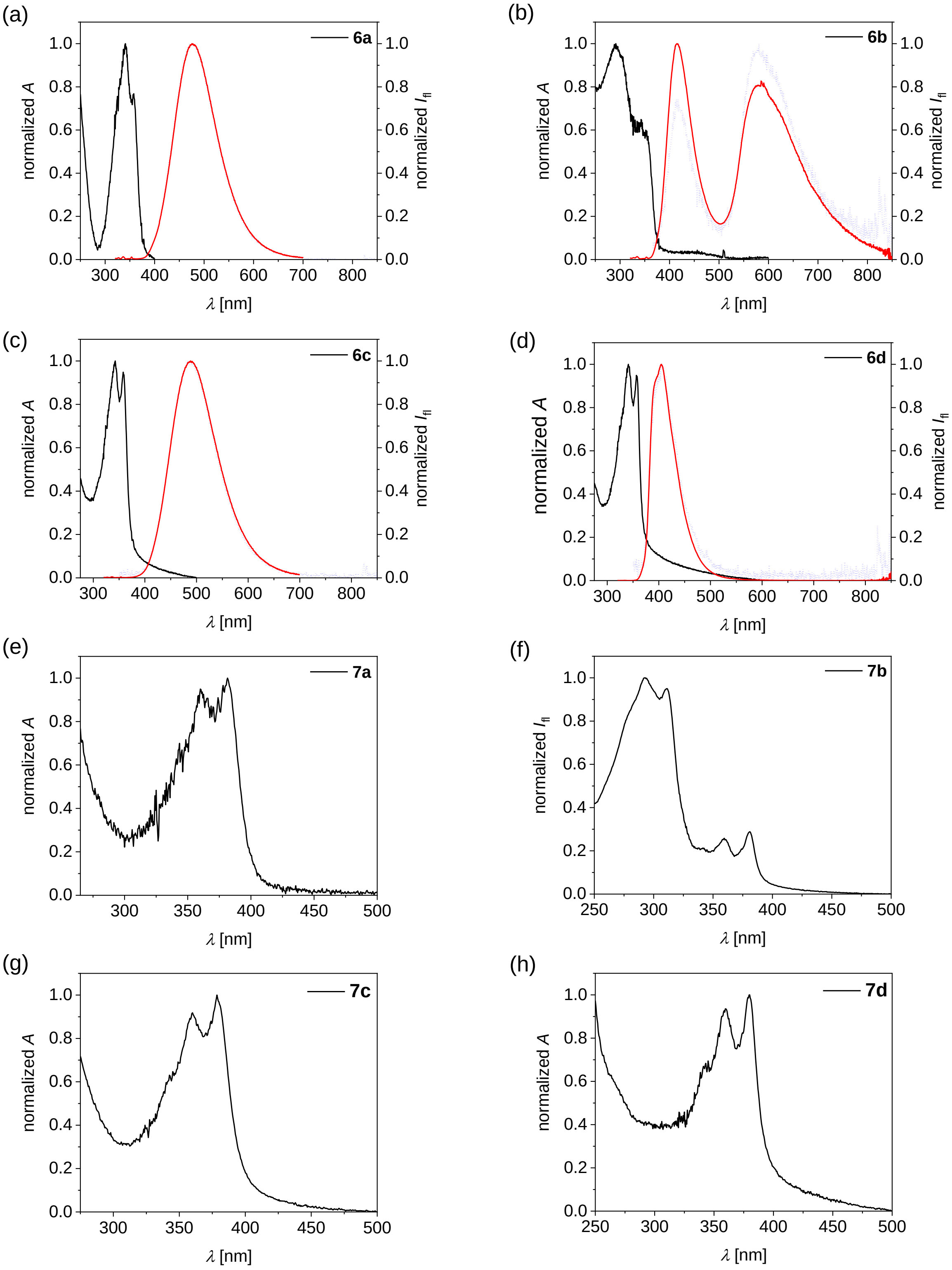

Figure S14. UV/Vis absorption (black solid line; spin-coated films) and fluorescence (dotted lines, red: measurement with front face setup (λ_{ex} = 320 nm), blue: measurement with integrations sphere (λ_{ex} = 325 nm); powder samples) spectra of (a) **6a**, (b) **6b**, (a) **6c**, (b) **6d**, (c) **7a**, (d) **7b**, (e) **7c** and (f) **7d** (no fluorescence spectra for non-emissive compounds **7a–d**).

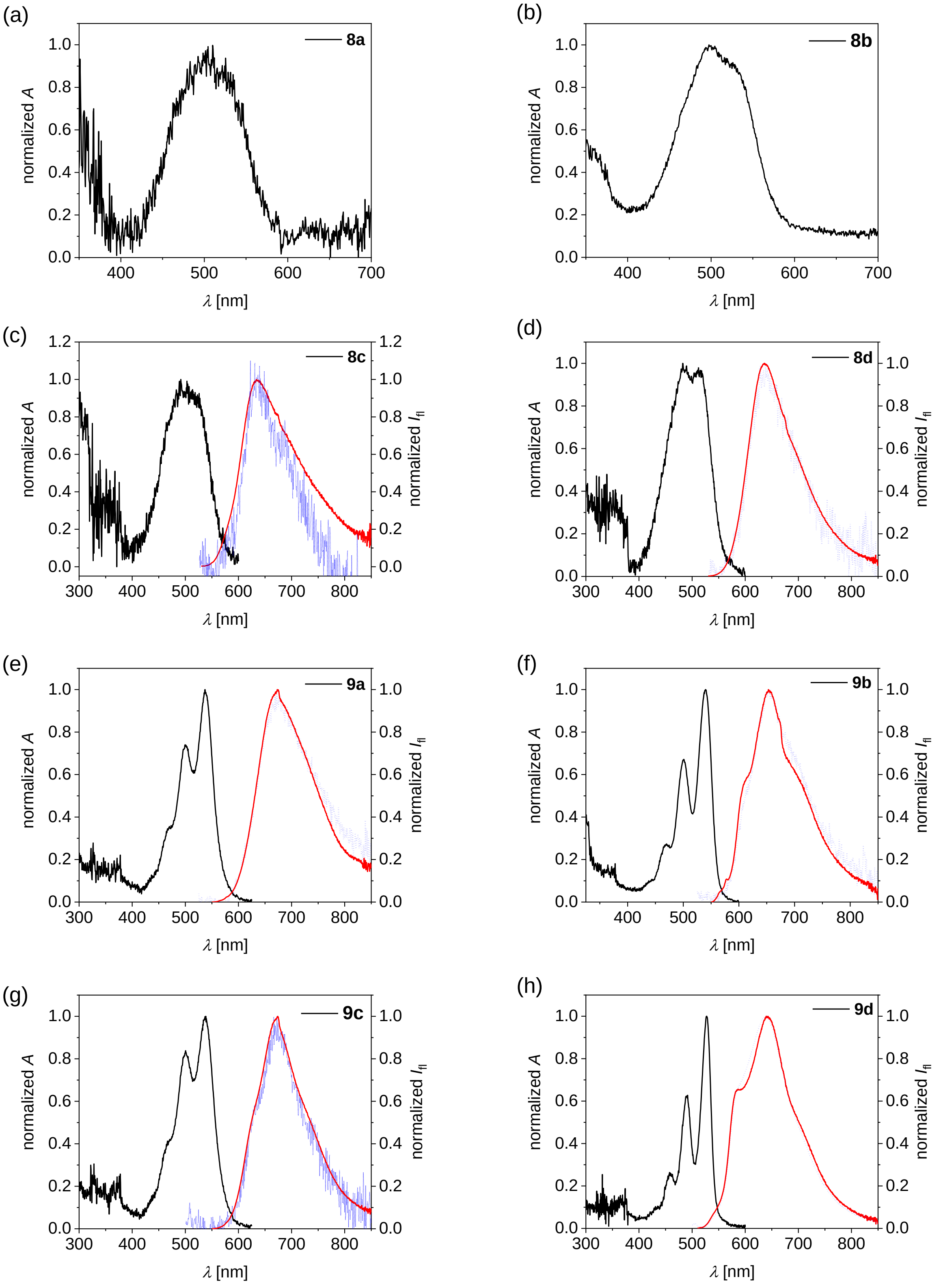

Figure S15. UV/Vis absorption (black solid line; spin-coated films) and fluorescence (red solid lines: measurement in front face setup (**8**: λ_{ex} = 470 nm, **9**: λ_{ex} = 490 nm), blue dotted lines: measurement with integrations sphere (λ_{ex} = 490 nm); powder samples) spectra of (a) **8a**, (b) **8b**, (a) **8c**, (b) **8d**, (c) **9a**, (d) **9b**, (e) **9c** and (f) **9d** (no fluorescence spectra for non-emissive compounds **8a**–**b**).

Table S15. Comparison of optical properties from **6**–**9** in the solid-state.

	λ_{abs} [nm][a]	λ_{em} [nm][b]	Φ_f [%][c]
**6a**	357, 343	478	18
**6b**	[d]	415, 582	3
**6c**	358, 343	490	7
**6d**	357, 341	405	1
**7a**	382, 360	-	<0.5
**7b**	381, 360, 311, 293	-	<0.5
**7c**	379, 360	-	<0.5
**7d**	380, 360	-	<0.5
**8a**	[d]	-	<0.5
**8b**	[d]	-	<0.5
**8c**	[d]	634	1
**8d**	512, 487	636	2
**9a**	539, 501	670	5
**9b**	540, 501, 469	653	3
**9c**	537, 501	670	5
**9d**	528, 489, 458	585, 640	17

[a] Absorption properties from spin-coated thin-films. [b] Fluorescence properties from bulk powder samples, which might be affected by reabsorption effects. [c] Effective fluorescence quantum yields of bulk powder samples were determined using an integration sphere. Represent the lower limit of the intrinsic fluorescence quantum yields due to reabsorption effects. [d] Precise determination not possible due to weak resolution of absorption spectra (low solubility of substrate).

Table S16. Comparison of fluorescence quantum yields from **6**–**9** in chloroform solution and bulk powder.

	Φ_f ($CHCl_3$) [%][a]	Φ_f (powder) [%][b]
**6a**	<0.5	18
**6b**	<0.5	3
**6c**	<0.5	7
**6d**	<0.5	1
**7a**	<0.5	<0.5
**7b**	<0.5	<0.5
**7c**	<0.5	<0.5
**7d**	<0.5	<0.5
**8a**	87 +/-4	<0.5
**8b**	86 +/-4	<0.5
**8c**	84 +/-4	1
**8d**	84 +/-4	2
**9a**	95 +/-2	5
**9b**	96 +/-1	3
**9c**	97 +/-1	5
**9d**	93 +/-2	17

[a] Fluorescence quantum yields were determined using the relative method (A < 0.05) and *N*,*N*'-bis(2,6-diisopropylphenyl)perylene-3,4:9,10-bis(dicarboximide) (Φ_f ($CHCl_3$) = 1.00). [b] Fluorescence quantum yields of bulk powder samples were determined using an integration sphere.

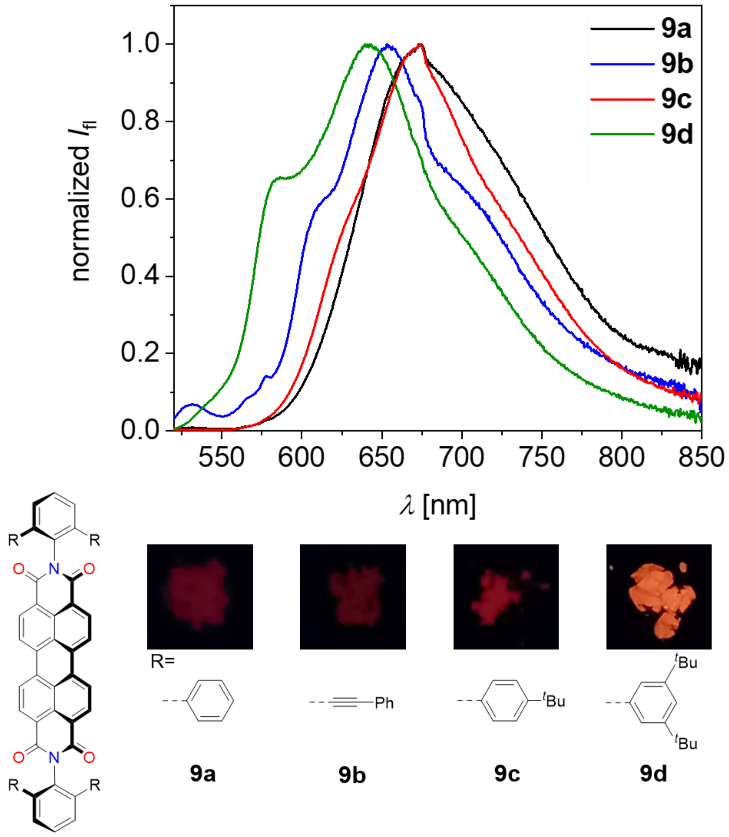

Figure S16. Solid-state (powder) fluorescence spectra of **9a–d** (top) and photographs of powder samples from **9a–d** under UV–light irradiation.

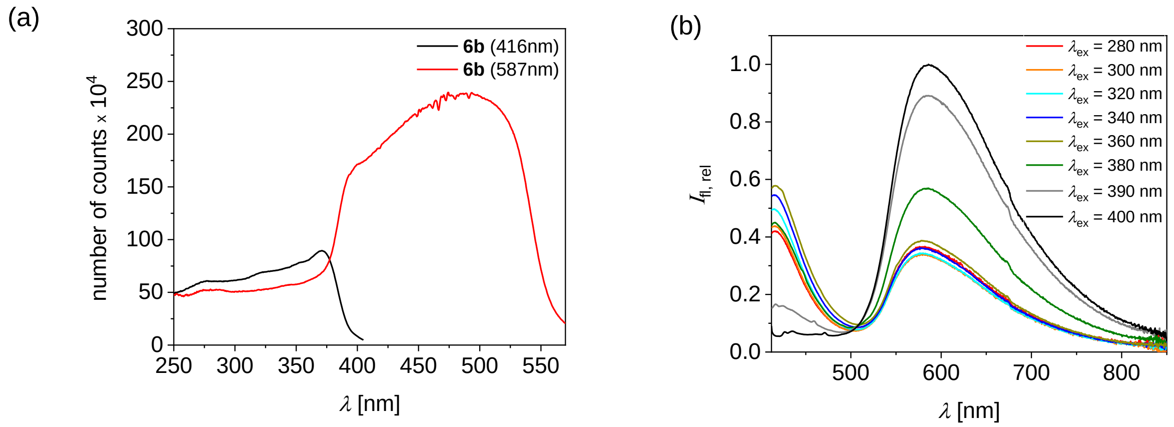

Figure S17. (a) Excitation spectra of **6b** (black line: λ_{rec} = 416 nm; red line: λ_{rec} = 587 nm). (b) Relative excitation-dependent fluorescence spectra from powder sample of **6b**.

Table S17. Determined fluorescence lifetime decays of **6a–d** (λ_{ex} = 378 nm) in powder samples.

	λ_{det} [nm]	τ [ns]
**6a**	477	τ_1 = 12.97 ± 0.64 (5.4%)<br>τ_2 = 30.45 ± 0.07 (94.6%)
**6b**	416	τ_1 = 2.57 ± 0.01
	578	τ_1 = 1.00 ± 0.04 (38.0%)<br>τ_2 = 3.39 ± 0.09 (62.0%)
**6c**	489	τ_1 = 6.33 ± 0.22 (7.3%)<br>τ_2 = 18.84 ± 0.04 (92.7%)
**6d**	405	τ_1 = 0.32 ± 0.002 (60.5%)<br>τ_2 = 1.90 ± 0.01 (39.5%)

NMR spectra

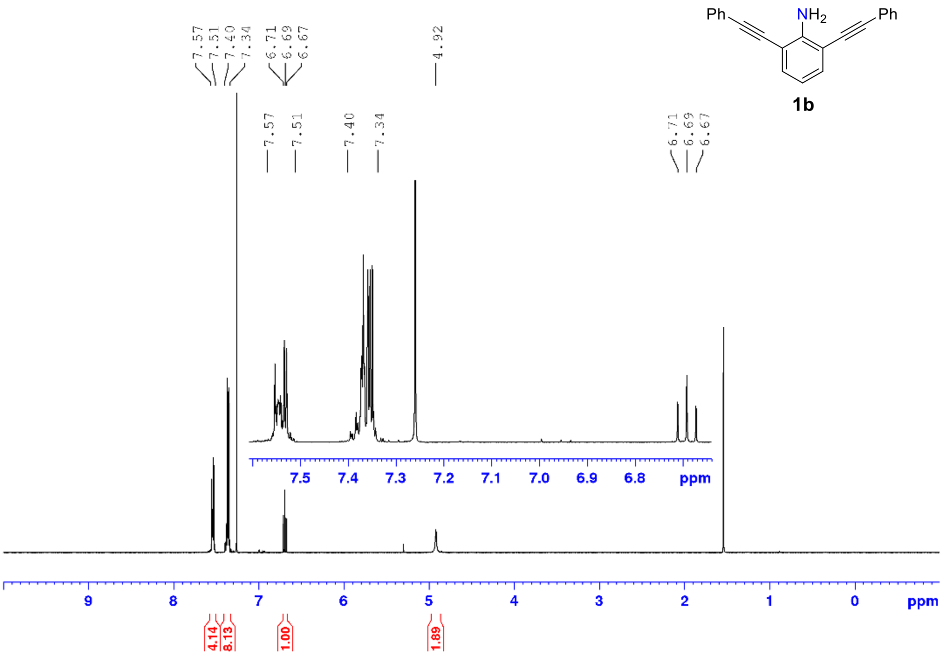

Figure S18. ^1H NMR (400 MHz) of **1b** in $CDCl_3$ at room temperature.

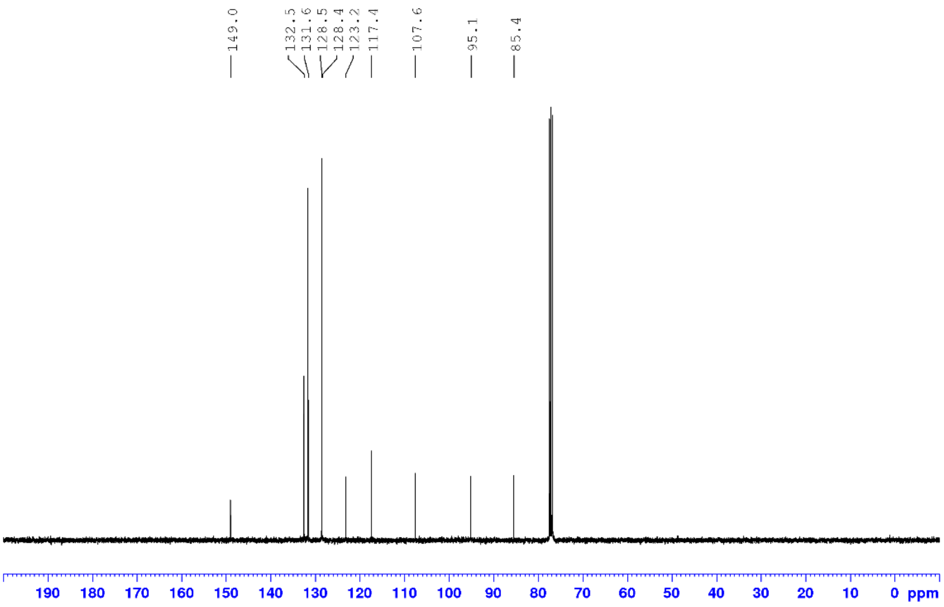

Figure S19. ^{13}C NMR (101 MHz) of **1b** in $CDCl_3$ at room temperature.

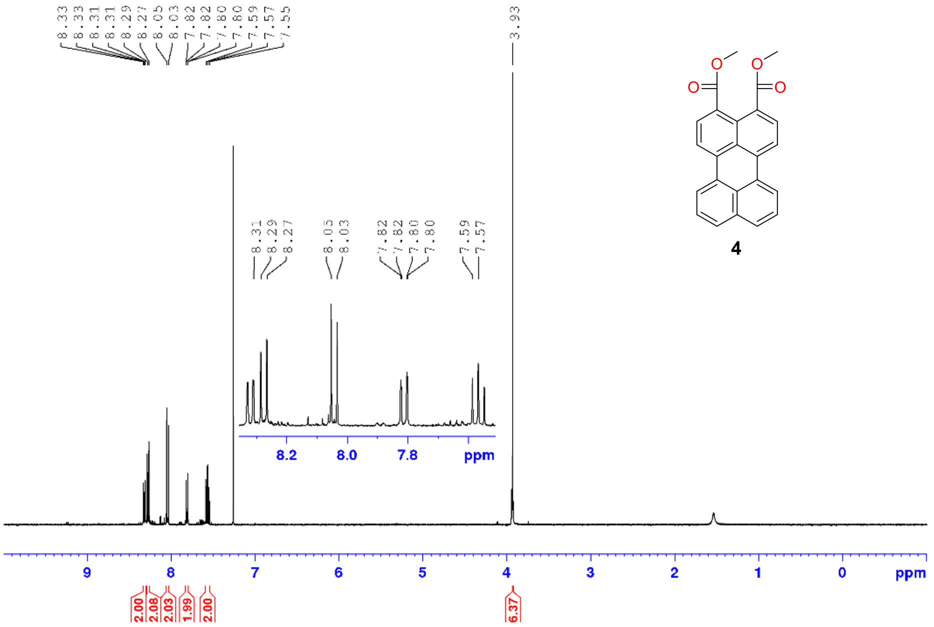

Figure S20. ^{1}H NMR (400 MHz) of **4** in $CDCl_3$ at room temperature.

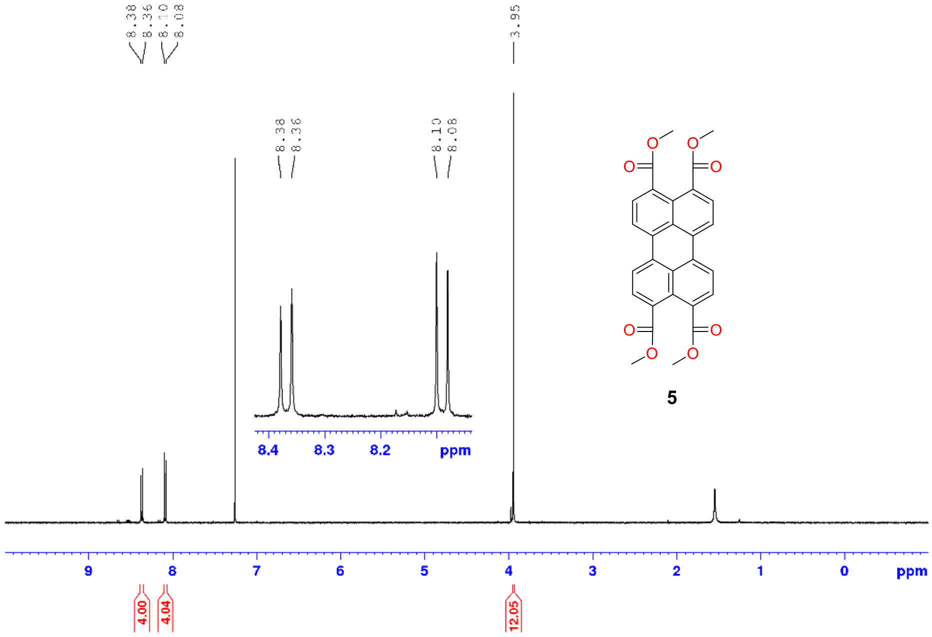

Figure S21. ^{1}H NMR (400 MHz) of **5** in $CDCl_3$ at room temperature.

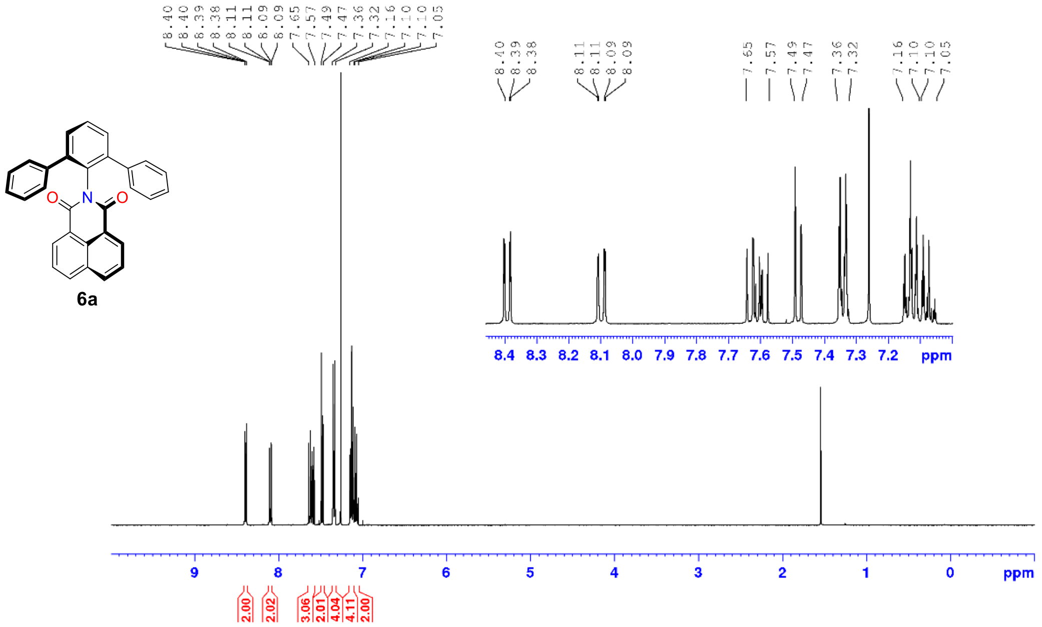

Figure S22. ^{1}H NMR (400 MHz) of **6a** in $CDCl_3$ at room temperature.

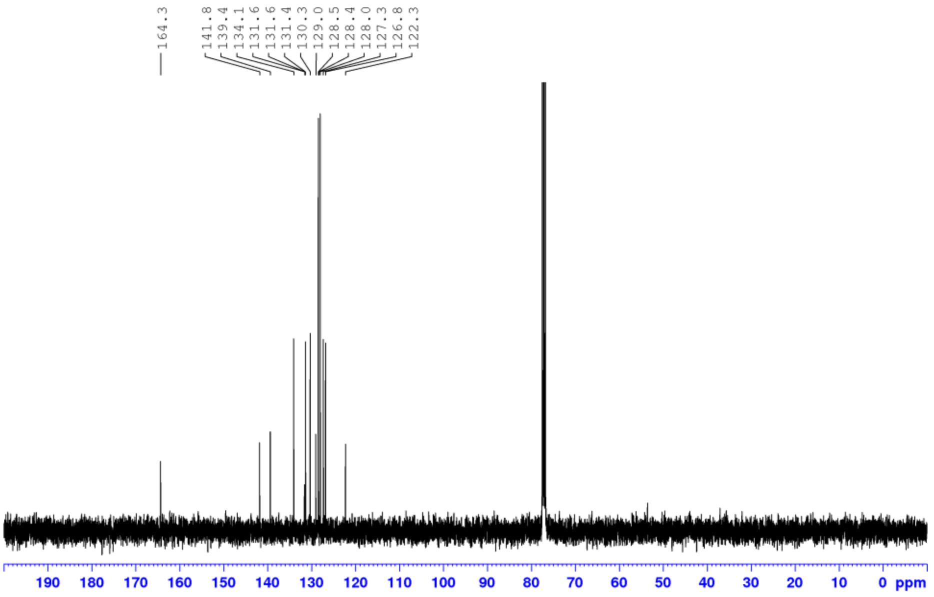

Figure S23. ^{13}C NMR (101 MHz) of **6a** in $CDCl_3$ at room temperature.

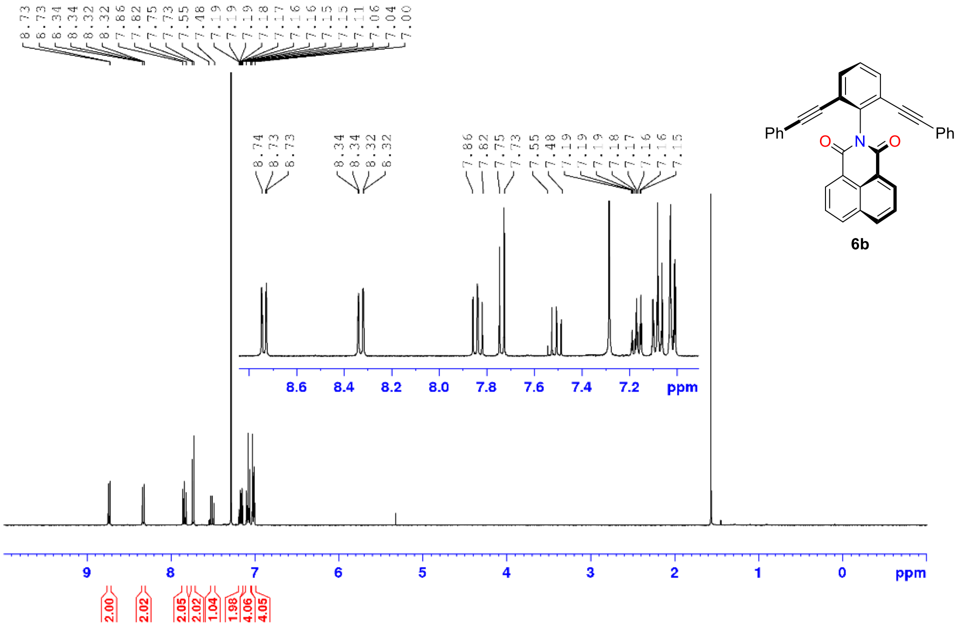

Figure S24. ^{1}H NMR (400 MHz) of **6b** in $CDCl_3$ at room temperature.

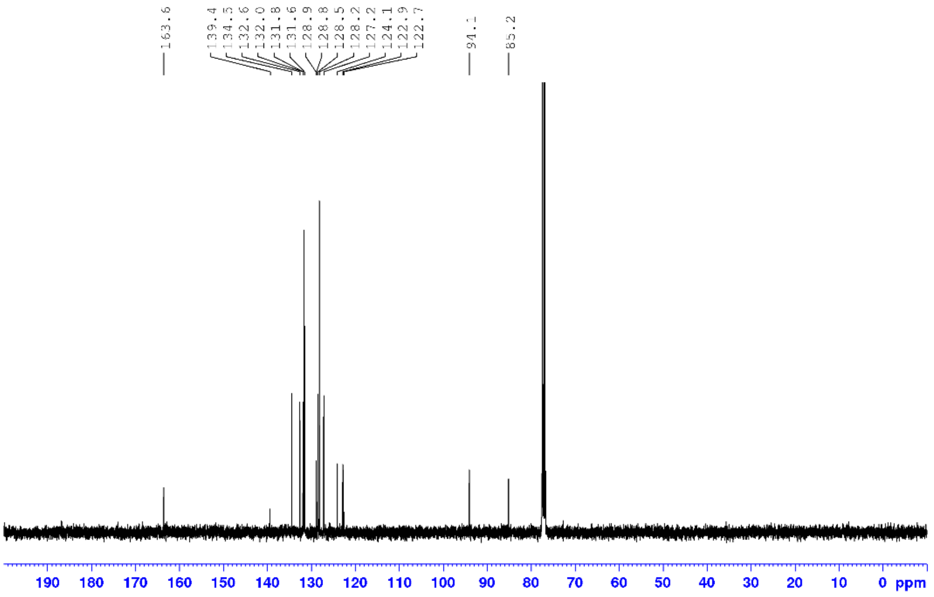

Figure S25. ^{13}C NMR (101 MHz) of **6b** in $CDCl_3$ at room temperature.

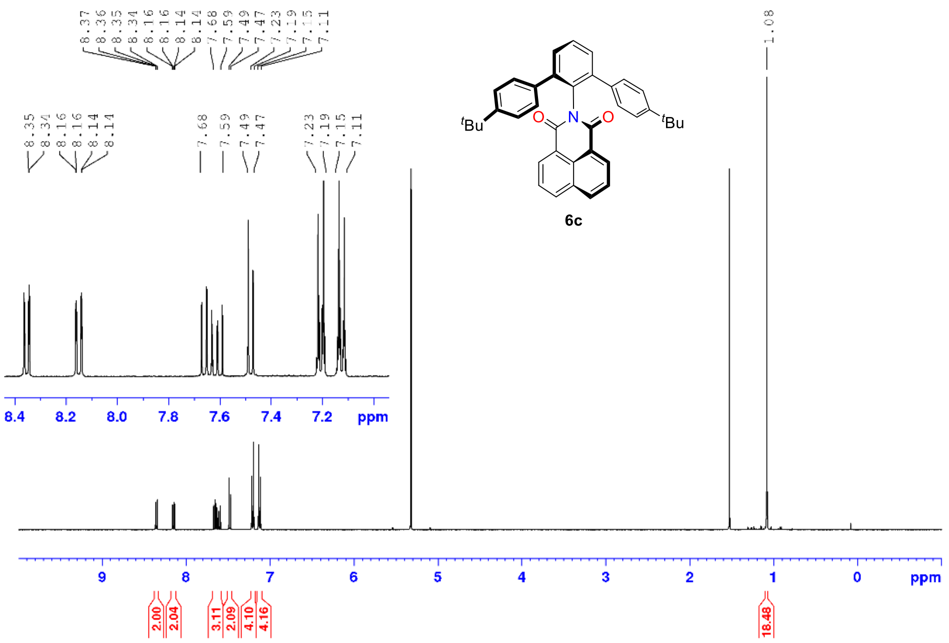

Figure S26. ^{1}H NMR (400 MHz) of **6c** in CD_2Cl_2 at room temperature.

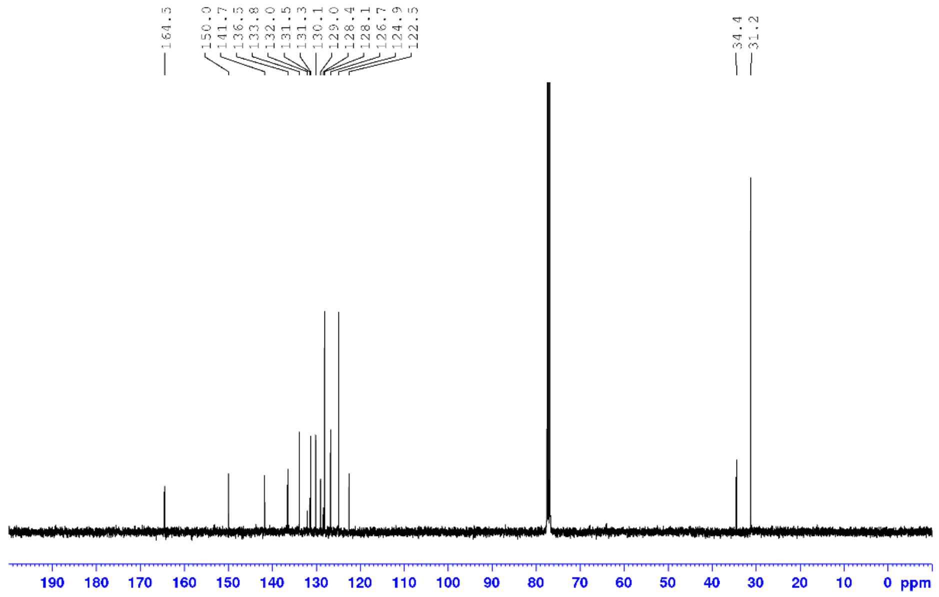

Figure S27. ^{13}C NMR (101 MHz) of **6c** in $CDCl_3$ at room temperature.

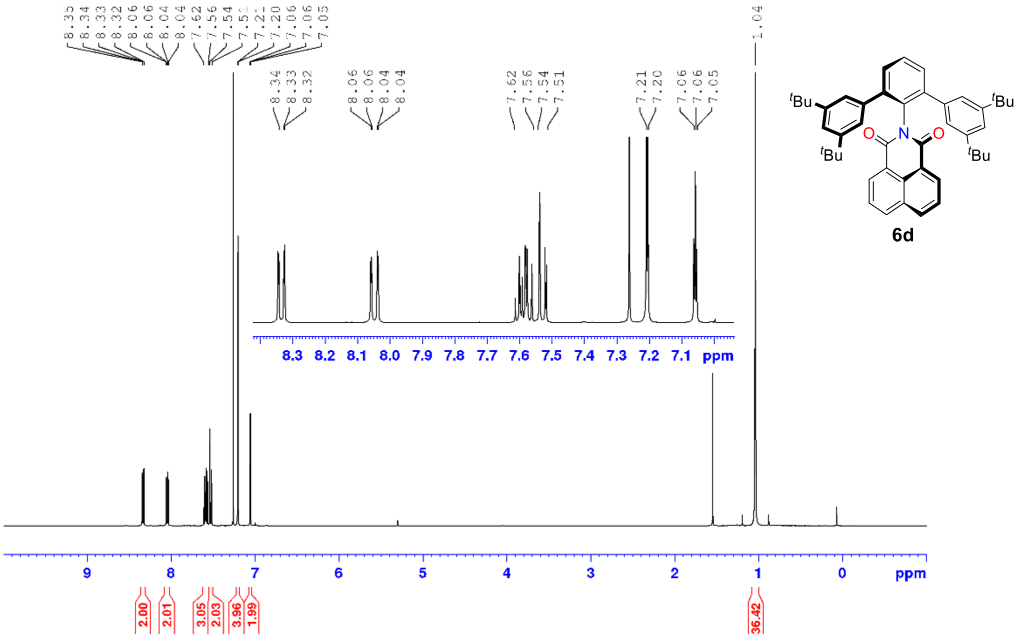

Figure S28. ^{1}H NMR (400 MHz) of **6d** in $CDCl_3$ at room temperature.

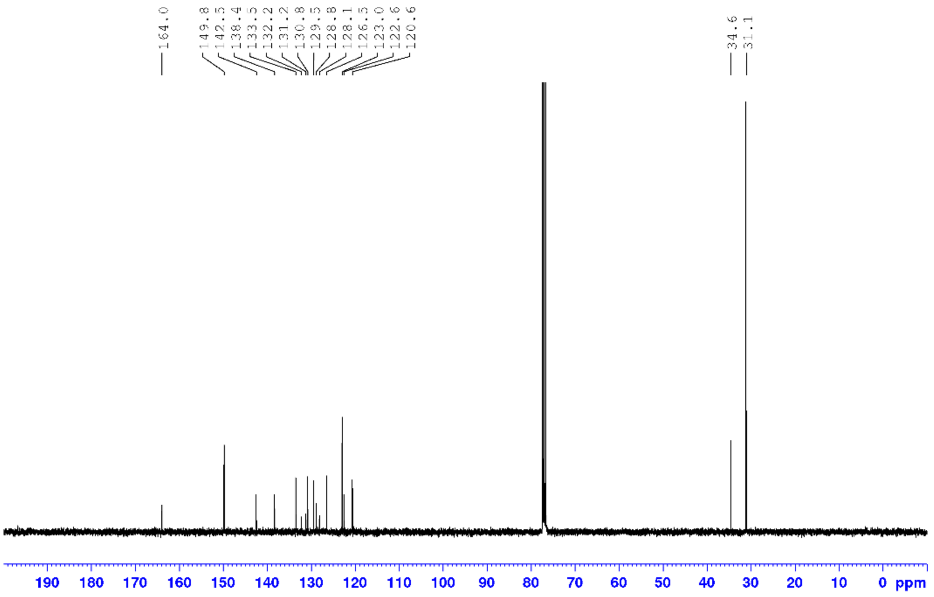

Figure S29. ^{13}C NMR (101 MHz) of **6d** in $CDCl_3$ at room temperature.

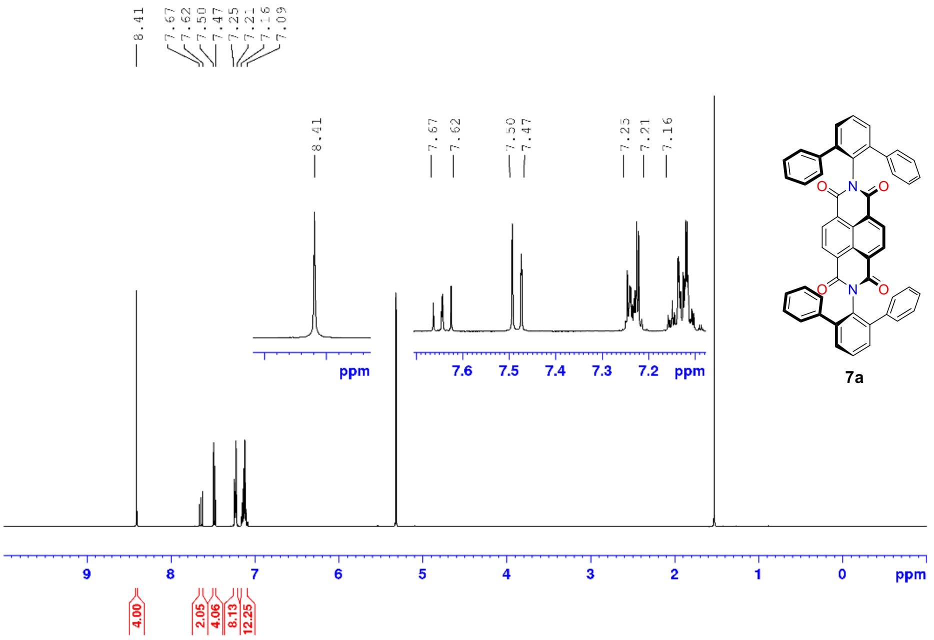

Figure S30. ^{1}H NMR (400 MHz) of **7a** in CD_2Cl_2 at room temperature.

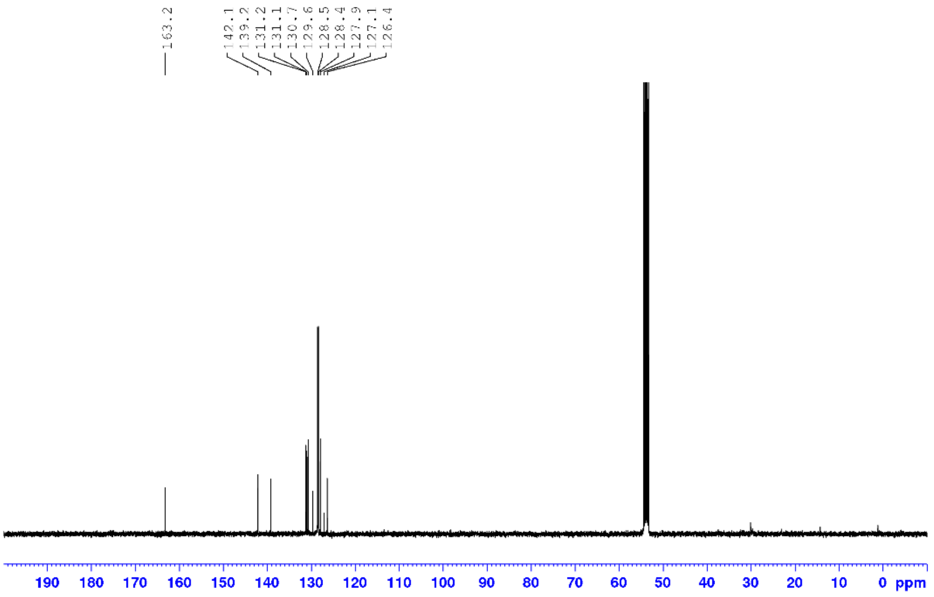

Figure S31. ^{13}C NMR (101 MHz) of **7a** in CD_2Cl_2 at room temperature.

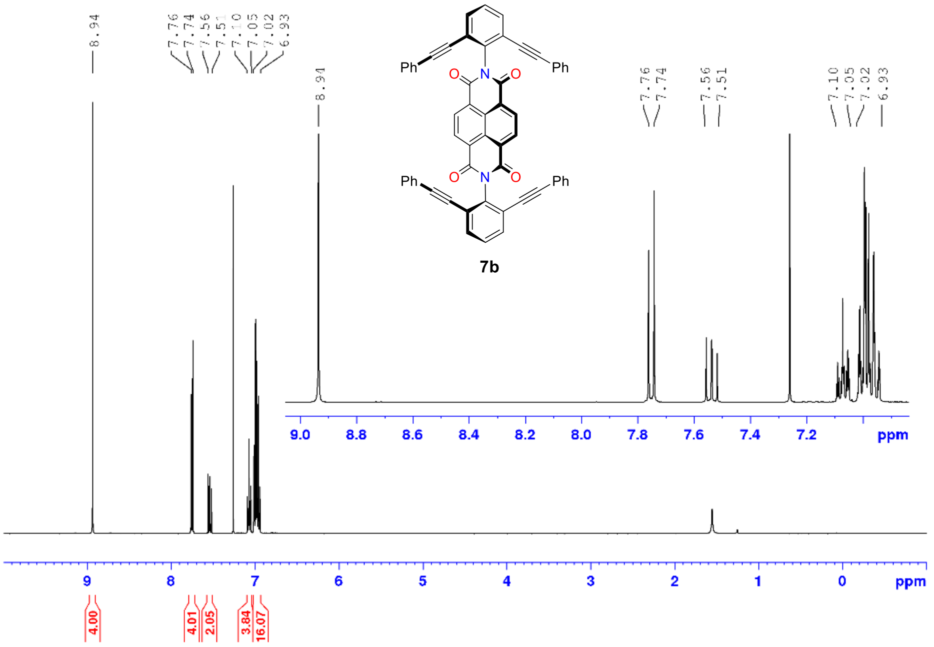

Figure S32. ^{1}H NMR (400 MHz) of **7b** in $CDCl_3$ at room temperature.

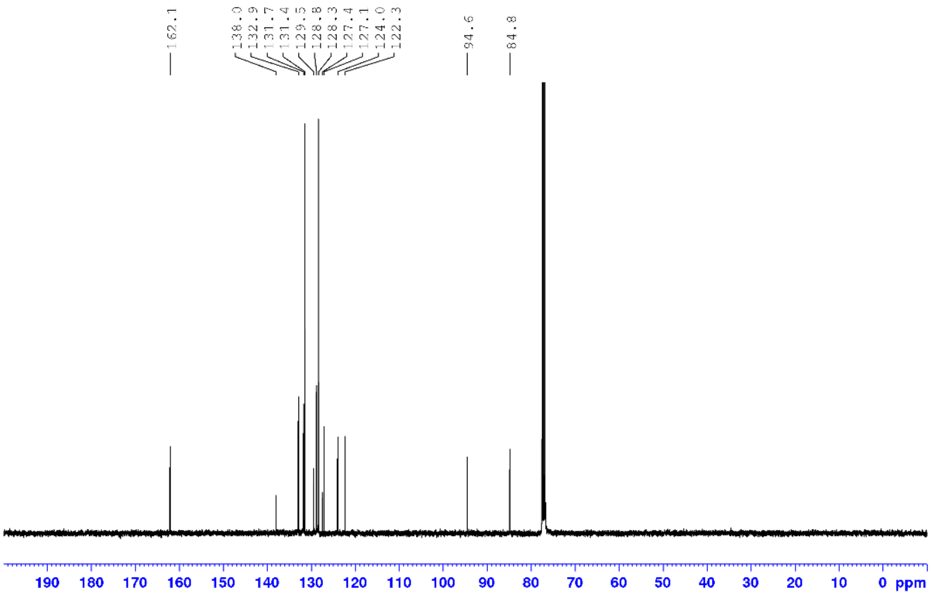

Figure S33. ^{13}C NMR (101 MHz) of **7b** in $CDCl_3$ at room temperature.

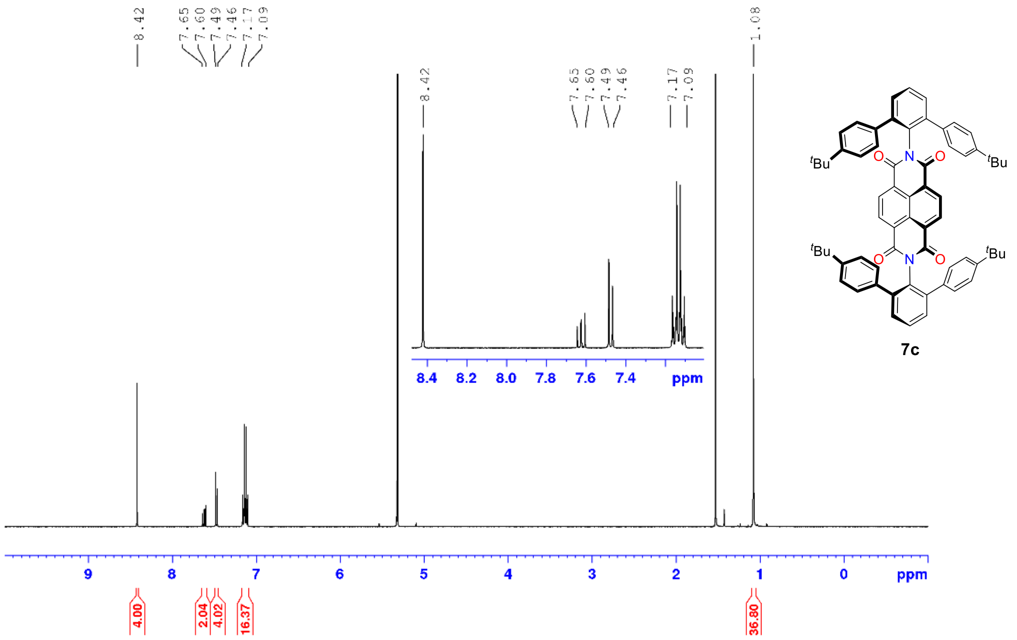

Figure S34. ^{1}H NMR (400 MHz) of **7c** in CD_2Cl_2 at room temperature.

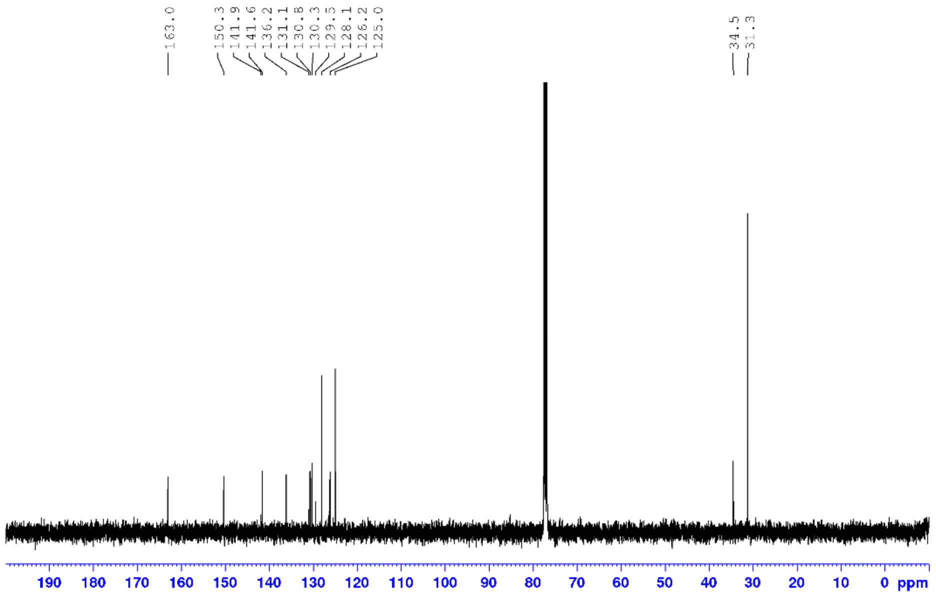

Figure S35. ^{13}C NMR (101 MHz) of **7c** in $CDCl_3$ at room temperature.

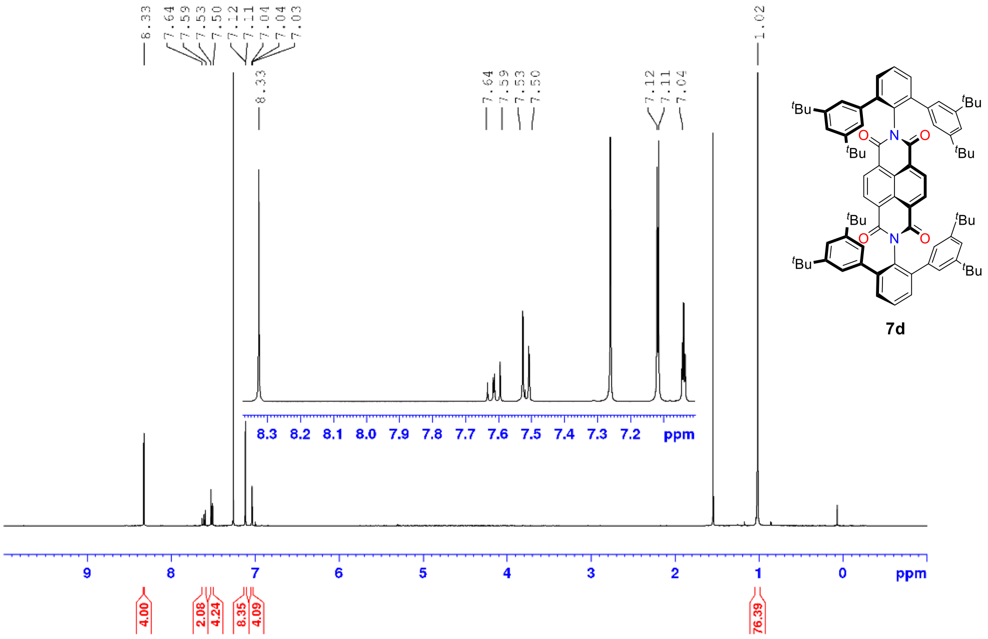

Figure S36. ^{1}H NMR (400 MHz) of **7d** in $CDCl_3$ at room temperature.

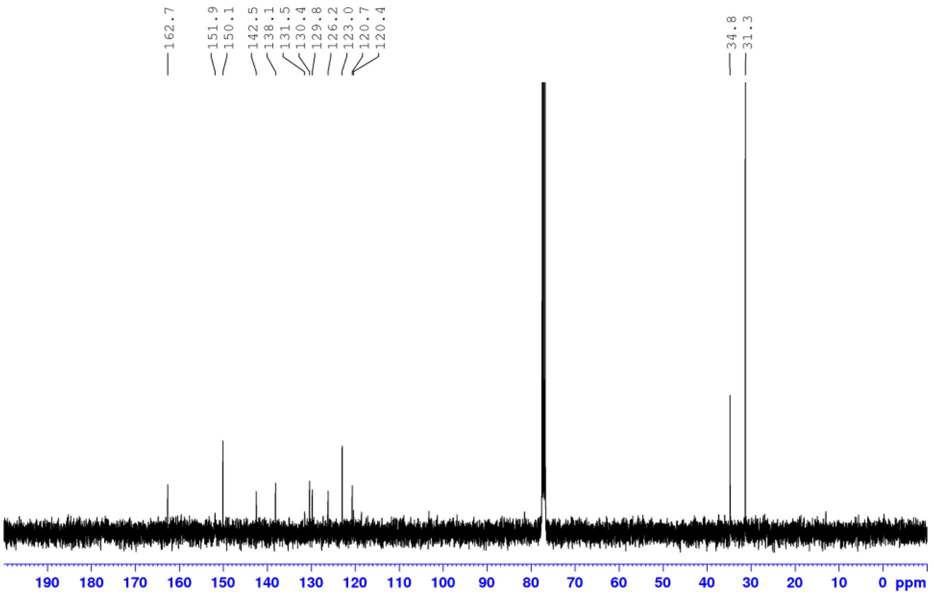

Figure S37. ^{13}C NMR (101 MHz) of **7d** in $CDCl_3$ at room temperature.

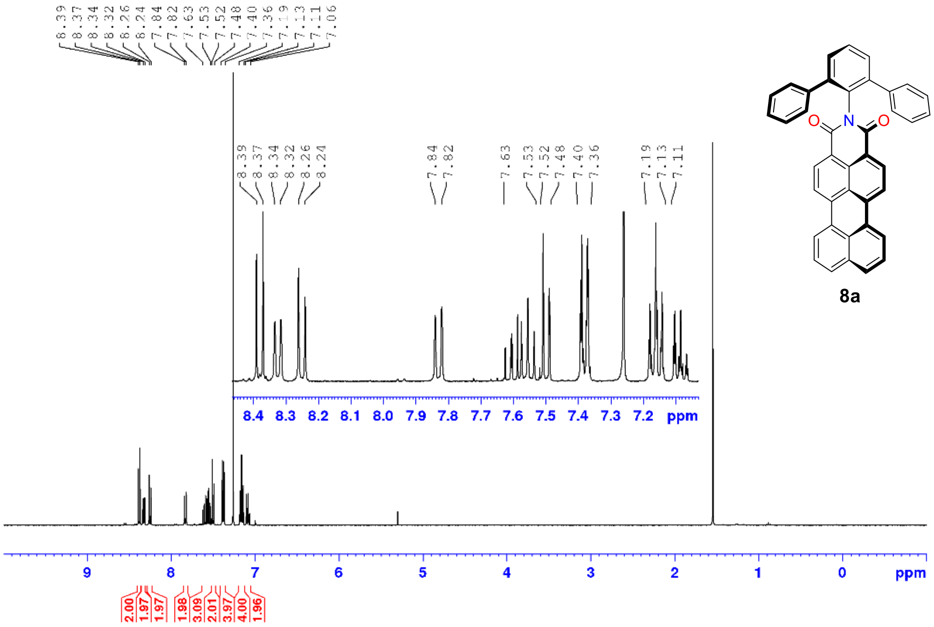

Figure S38. ^1H NMR (400 MHz) of **8a** in $CDCl_3$ at room temperature.

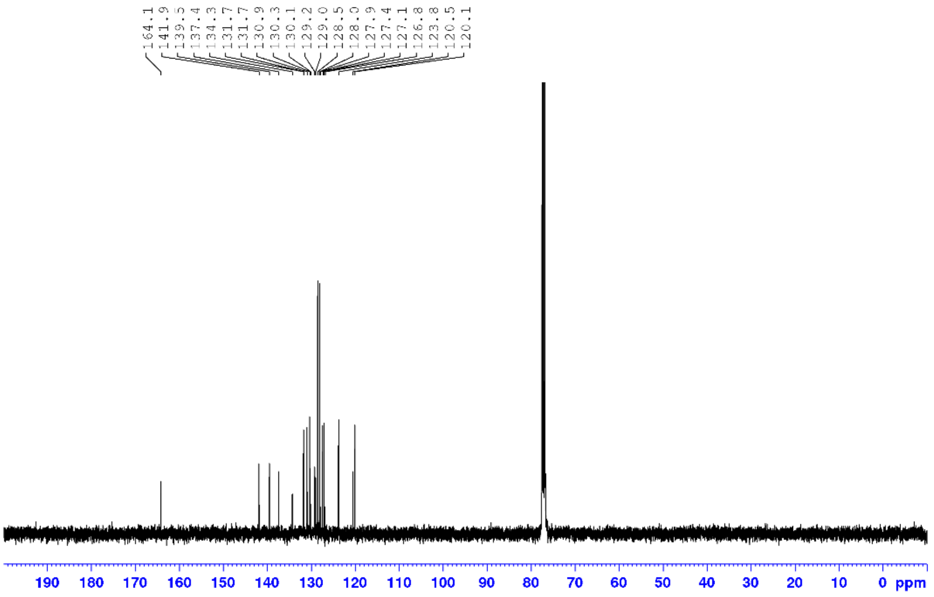

Figure S39. ^{13}C NMR (101 MHz) of **8a** in $CDCl_3$ at room temperature.

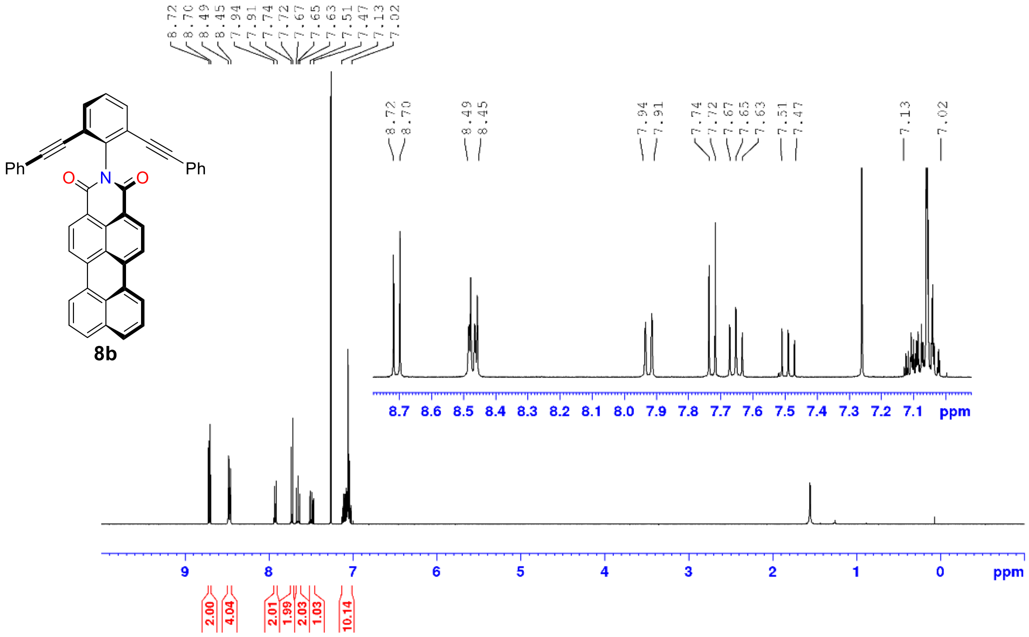

Figure S40. ^{1}H NMR (400 MHz) of **8b** in $CDCl_3$ at room temperature.

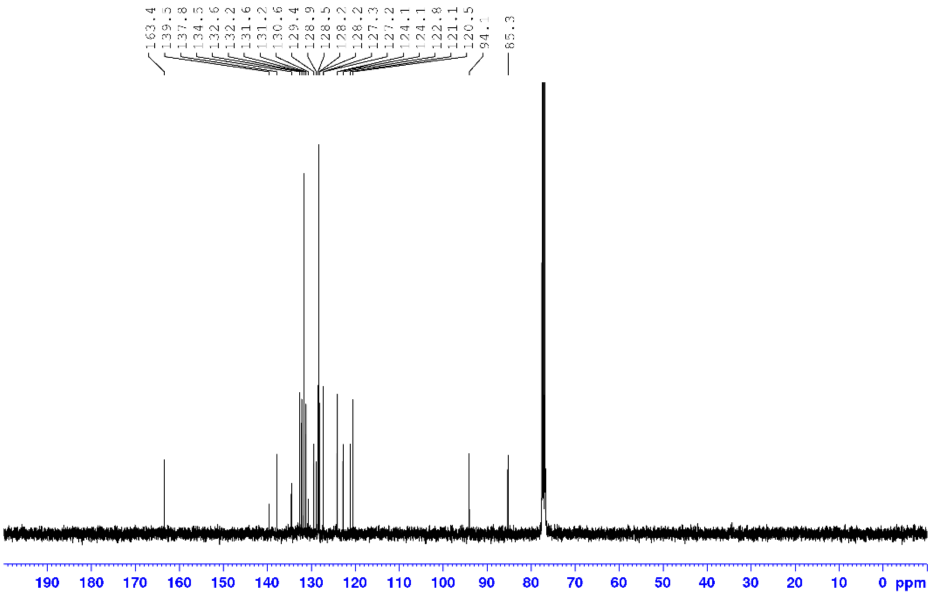

Figure S41. ^{13}C NMR (101 MHz) of **8b** in $CDCl_3$ at room temperature.

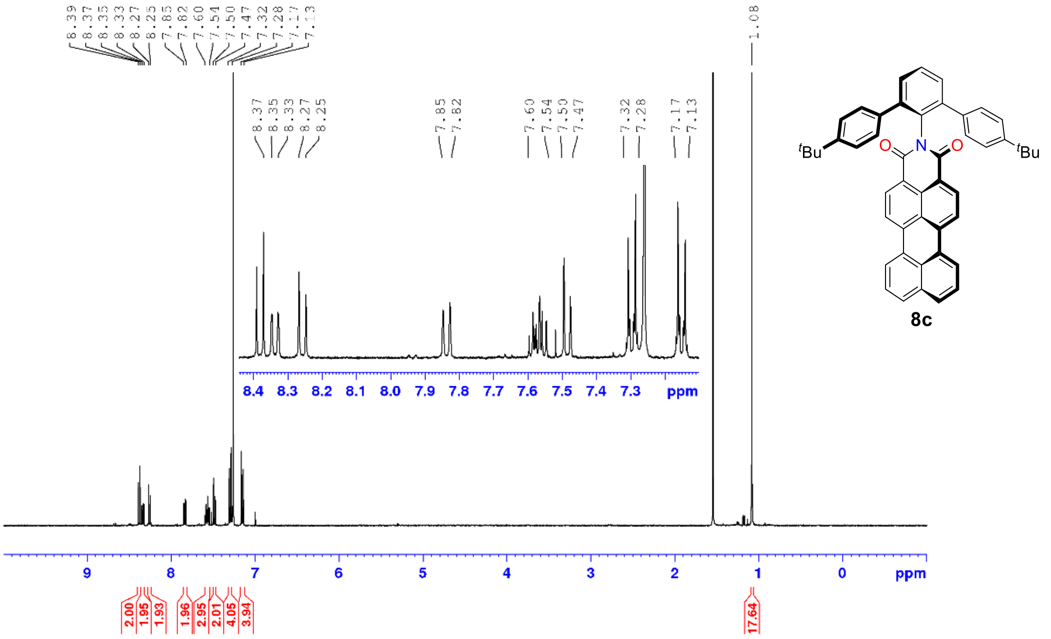

Figure S42. ^{1}H NMR (400 MHz) of **8c** in $CDCl_3$ at room temperature.

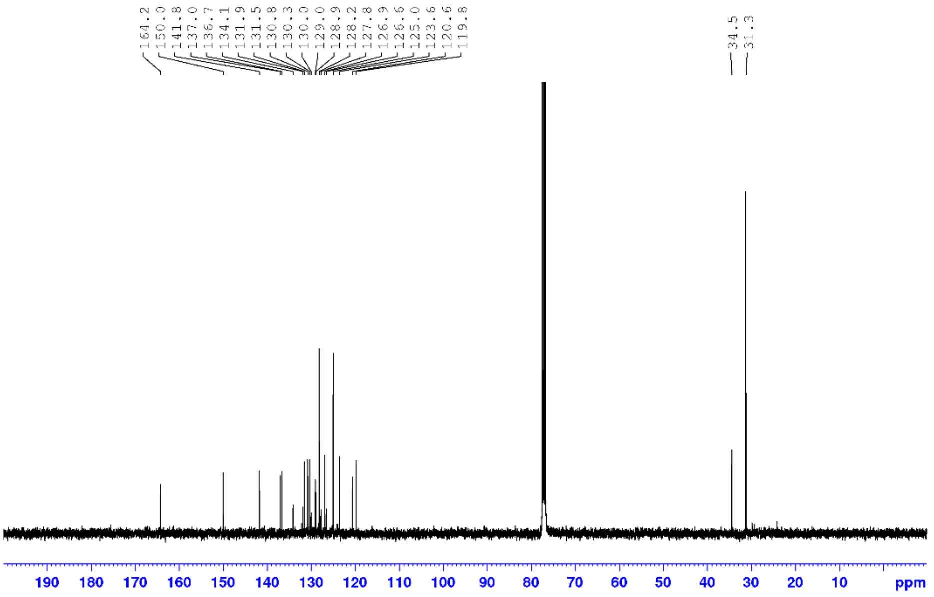

Figure S43. ^{13}C NMR (101 MHz) of **8c** in $CDCl_3$ at room temperature.

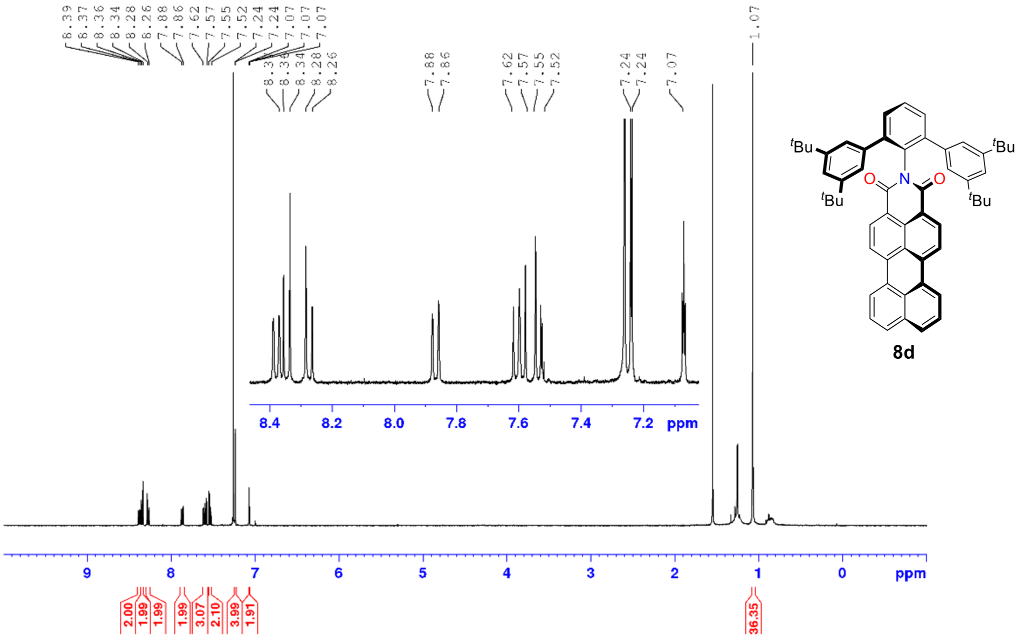

Figure S44. ^{1}H NMR (400 MHz) of **8d** in $CDCl_3$ at room temperature.

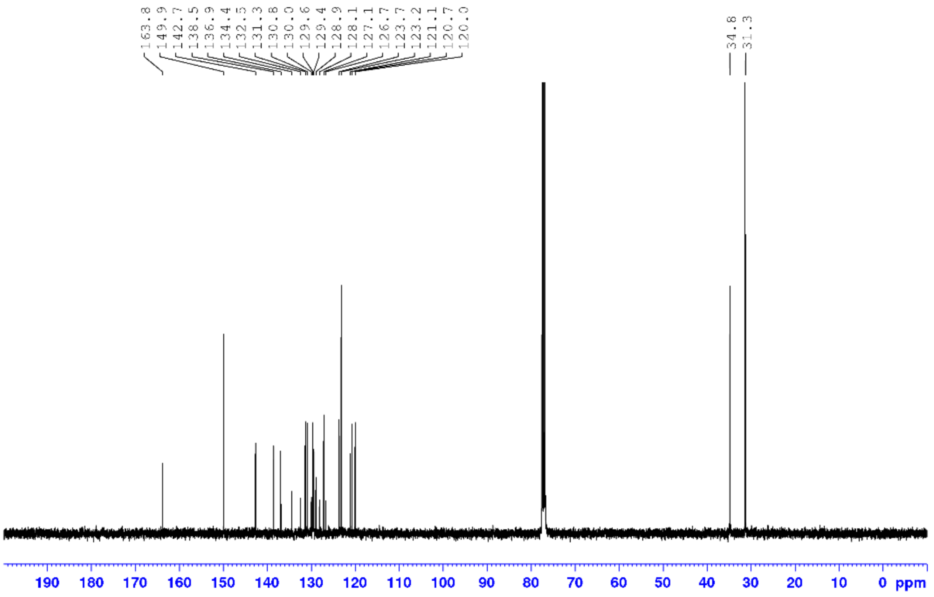

Figure S45. ^{13}C NMR (101 MHz) of **8d** in $CDCl_3$ at room temperature.

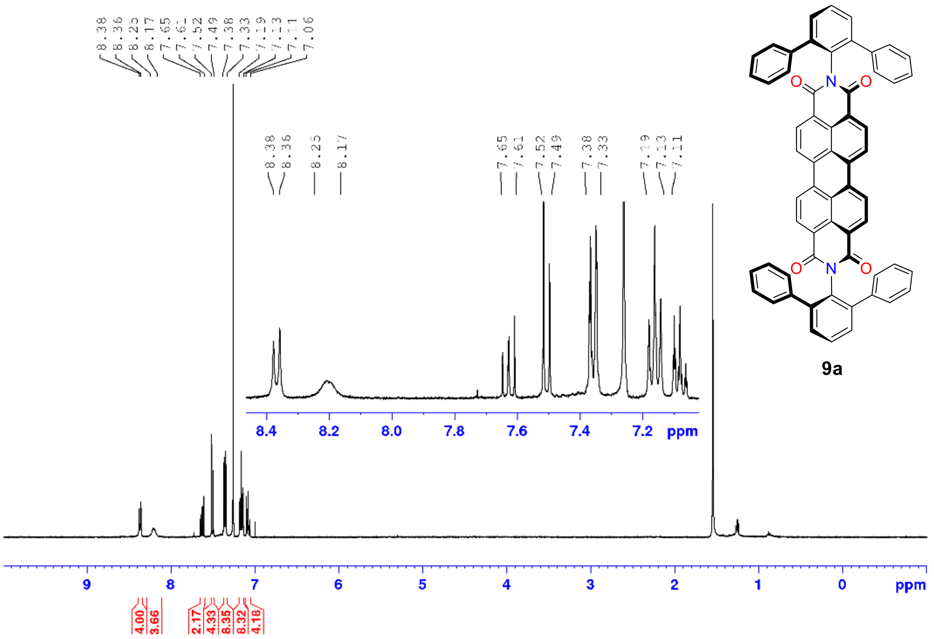

Figure S46. ^{1}H NMR (400 MHz) of **9a** in $CDCl_3$ at room temperature.

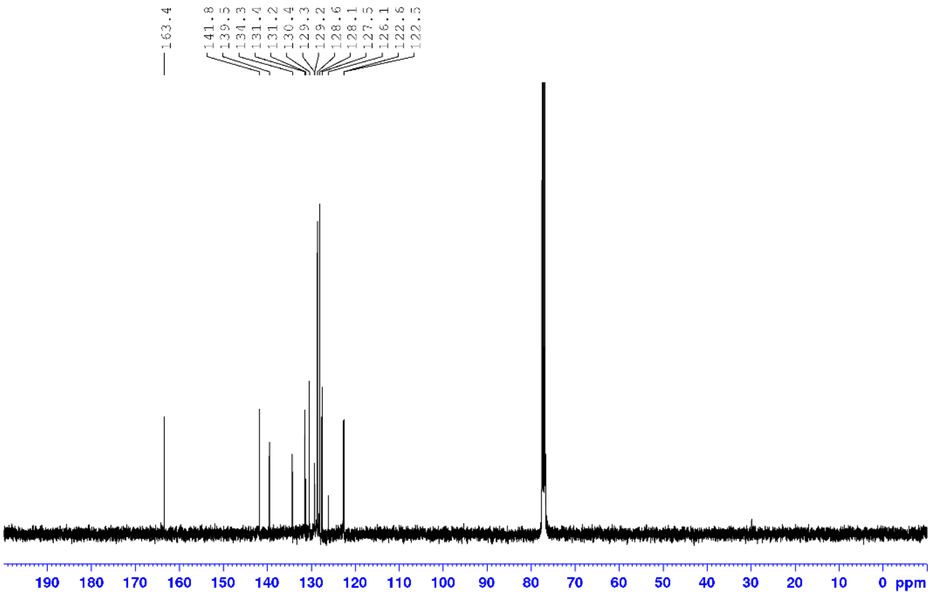

Figure S47. ^{13}C NMR (101 MHz) of **9a** in $CDCl_3$ at room temperature.

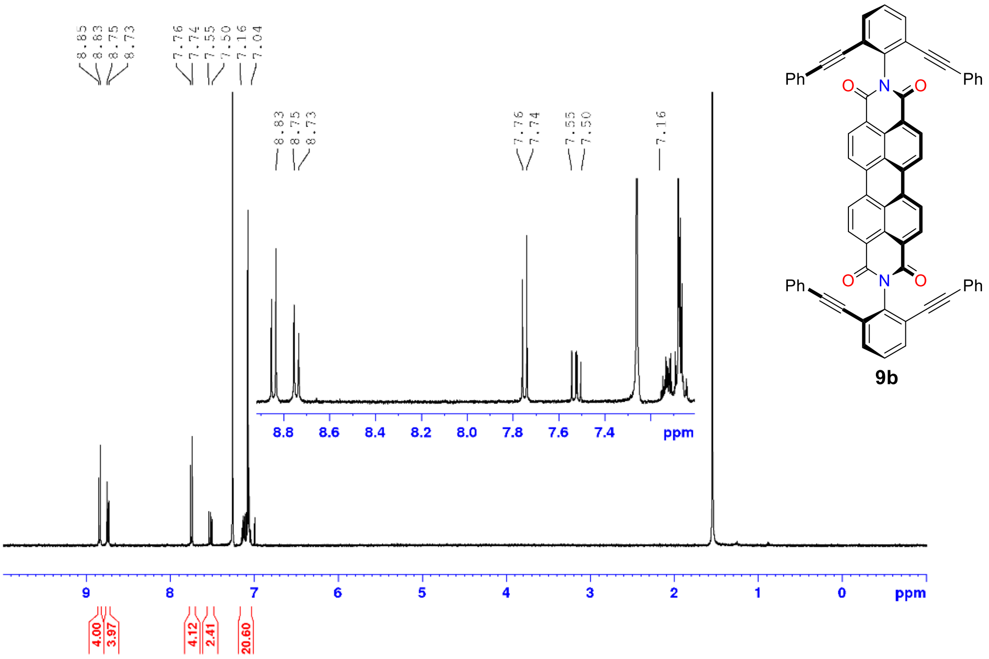

9b

Figure S48. ^{1}H NMR (400 MHz) of **9b** in $CDCl_3$ at room temperature.

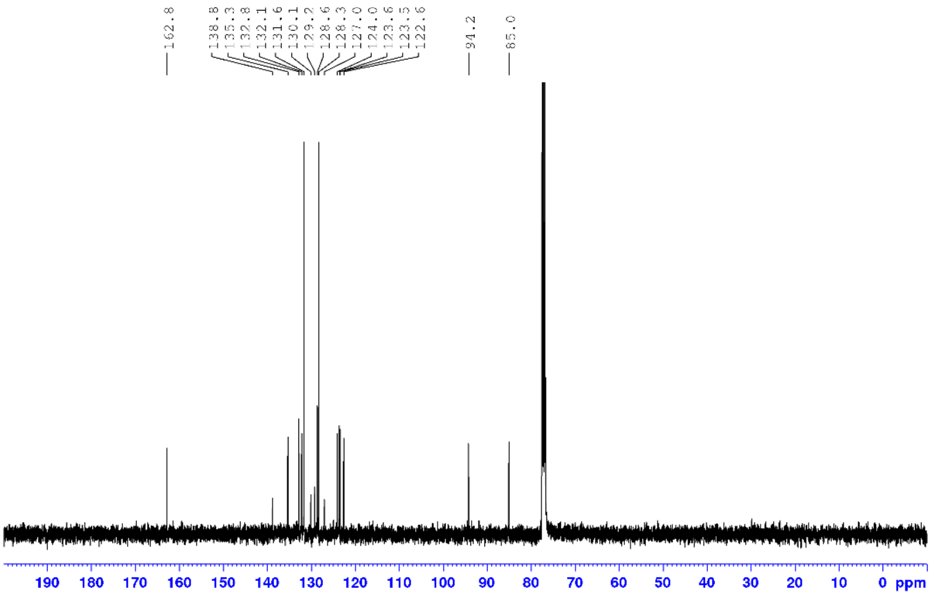

Figure S49. ^{13}C NMR (101 MHz) of **9b** in $CDCl_3$ at room temperature.

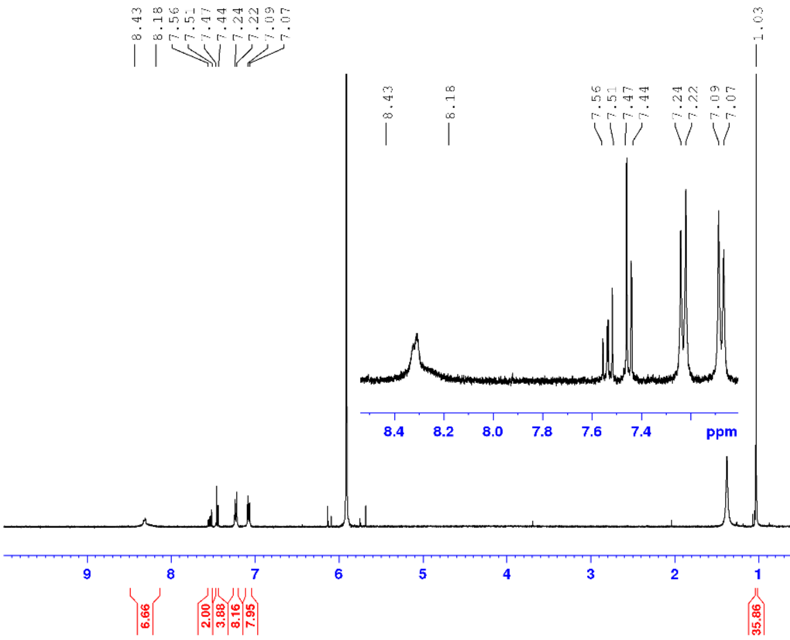

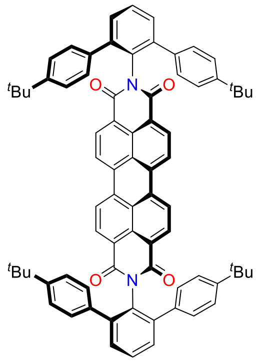

9c

Figure S50. ^{1}H NMR (400 MHz) of **9c** in $C_2D_2Cl_4$ at 373 K.

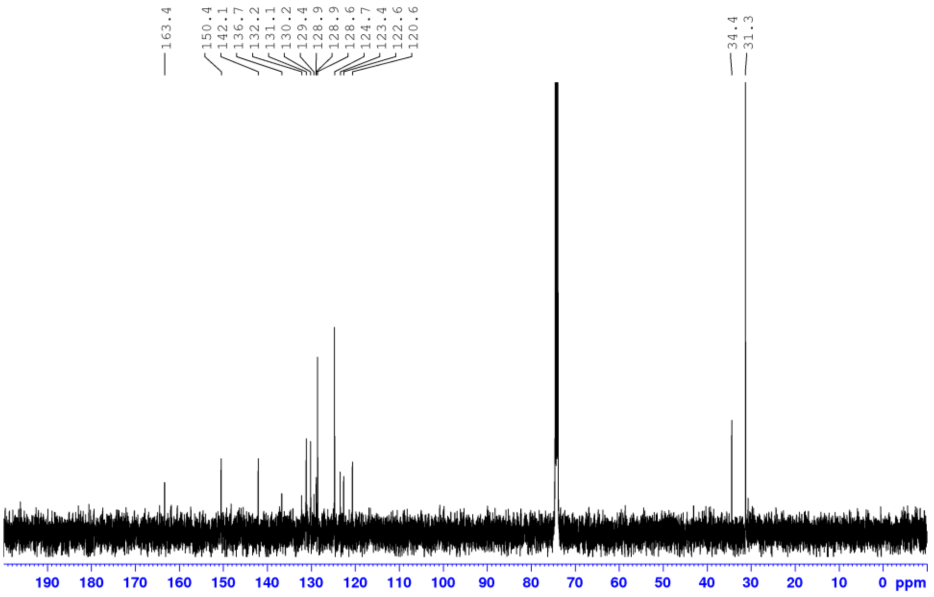

Figure S51. ^{13}C NMR (101 MHz) of **9c** in $C_2D_2Cl_4$ at 373 K.

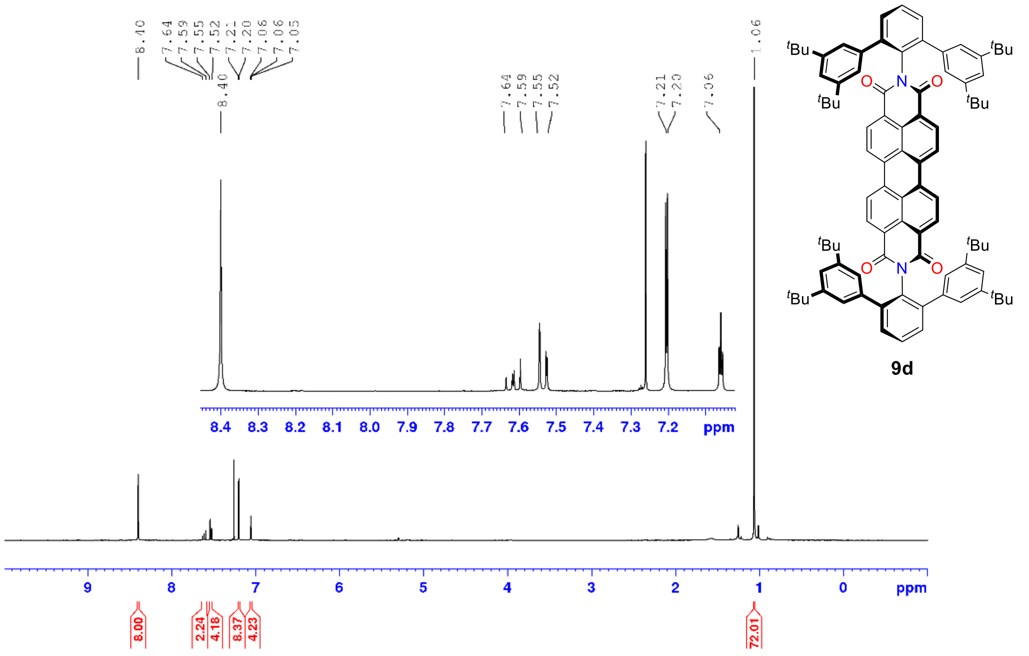

Figure S52. ^{1}H NMR (400 MHz) of **9d** in $CDCl_3$ at room temperature.

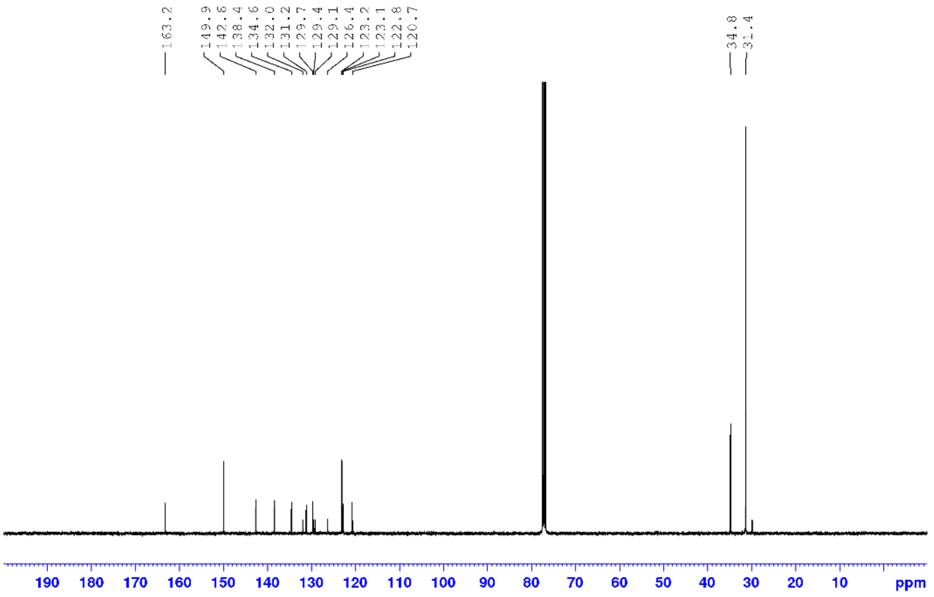

Figure S53. ^{13}C NMR (101 MHz) of **9d** in $CDCl_3$ at room temperature.

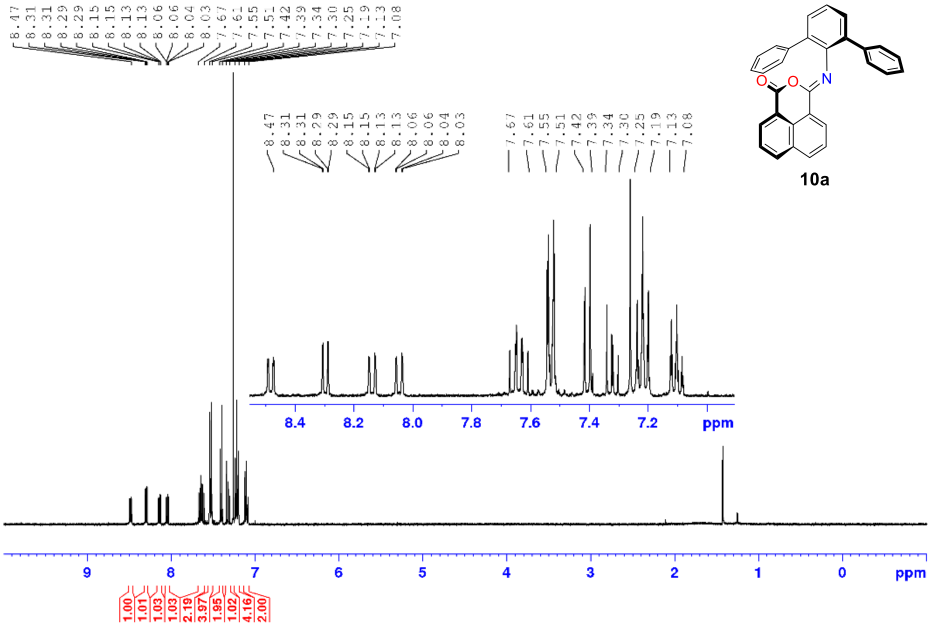

Figure S54. ^{1}H NMR (400 MHz) of **10a** in $CDCl_3$ at room temperature.

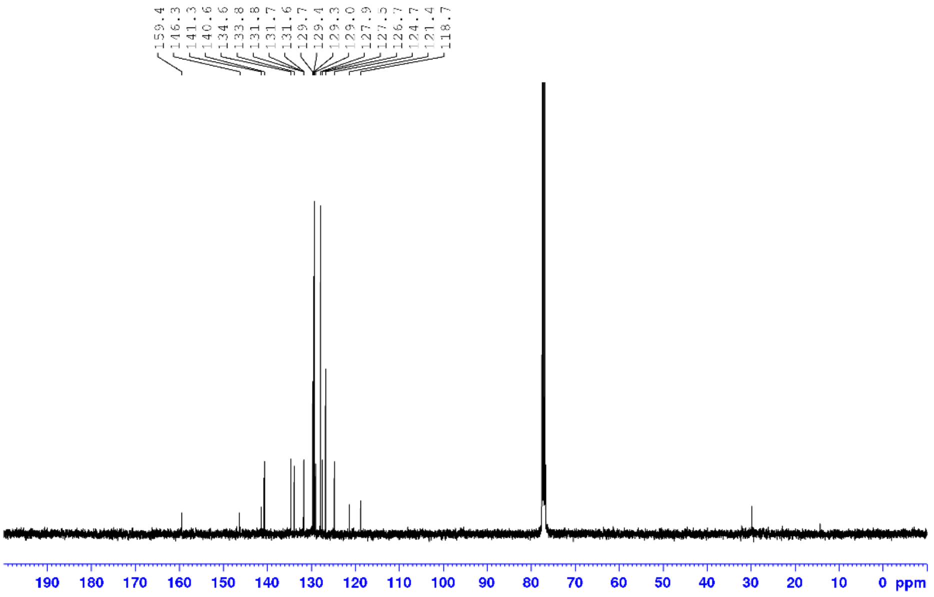

Figure S55. ^{13}C NMR (101 MHz) of **10a** in $CDCl_3$ at room temperature.

Mass spectra

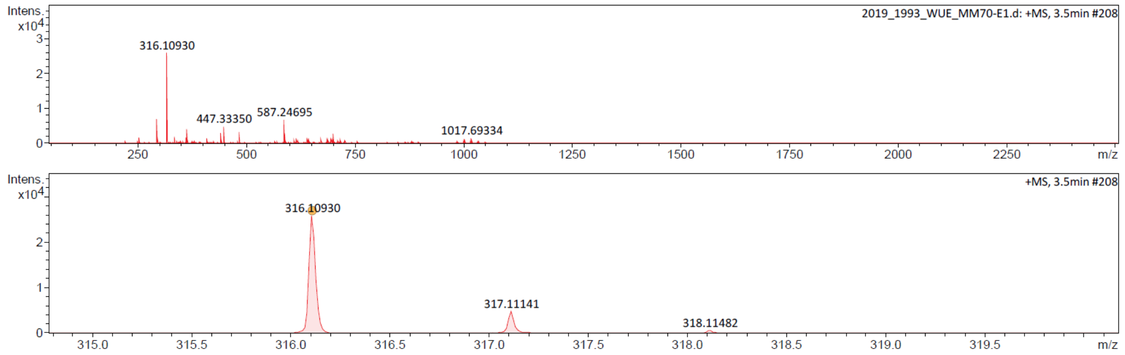

Meas. m/z	#	Ion Formula	m/z	err [ppm]	mSigma	# mSigma	Score	rdb	e¯ Conf	N-Rule
316.10930	1	C22H15NNa	316.10967	1.18	34.8	1	100.00	15.5	even	ok

Figure S56. HRMS (ESI-TOF, pos. mode, acetonitrile/chloroform 1/1) spectra of **1b**.

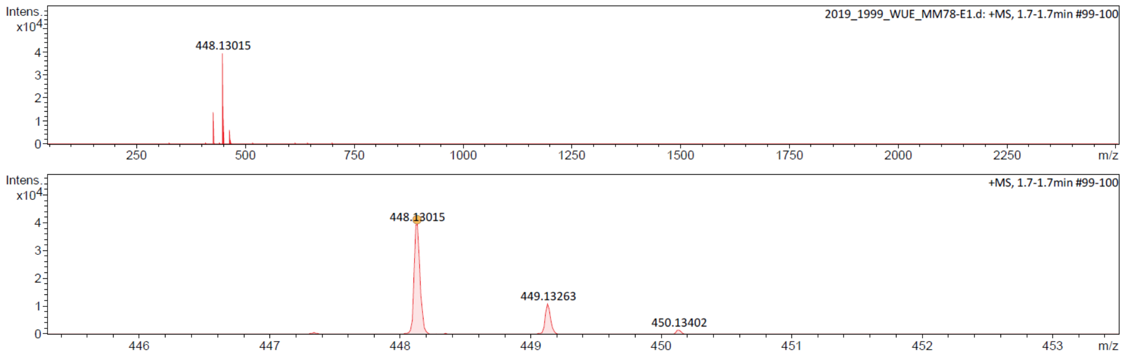

Meas. m/z	#	Ion Formula	m/z	err [ppm]	mSigma	# mSigma	Score	rdb	e¯ Conf	N-Rule
448.13015	1	C30H19NNaO2	448.13080	1.46	32.3	1	100.00	21.5	even	ok

Figure S57. HRMS (ESI-TOF, pos. mode, acetonitrile/chloroform 1/1) spectra of **6a**.

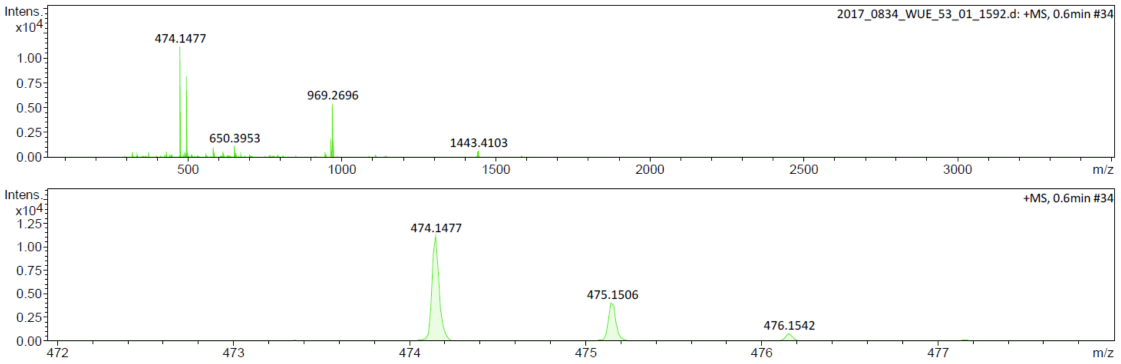

Meas. m/z	#	Ion Formula	m/z	err [ppm]	mSigma	# mSigma	Score	rdb	e¯ Conf	N-Rule
474.1477	1	C34H20NO2	474.1489	2.4	7.3	1	100.00	25.5	even	ok

Figure S58. HRMS (ESI-TOF, pos. mode, acetonitrile/chloroform 1/1) spectra of **6b**.

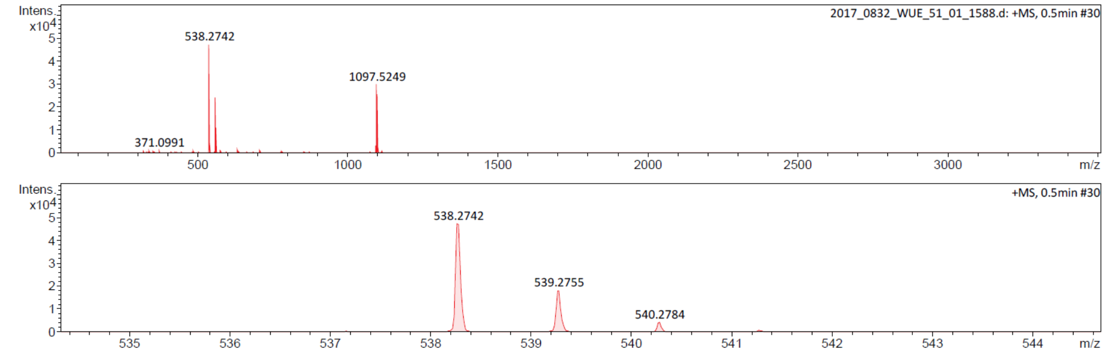

Meas. m/z	#	Ion Formula	m/z	err [ppm]	mSigma	# mSigma	Score	rdb	e¯ Conf	N-Rule
538.2742	1	C38H36NO2	538.2741	-0.3	18.2	1	100.00	21.5	even	ok

Figure S59. HRMS (ESI-TOF, pos. mode, acetonitrile/chloroform 1/1) spectra of **6c**.

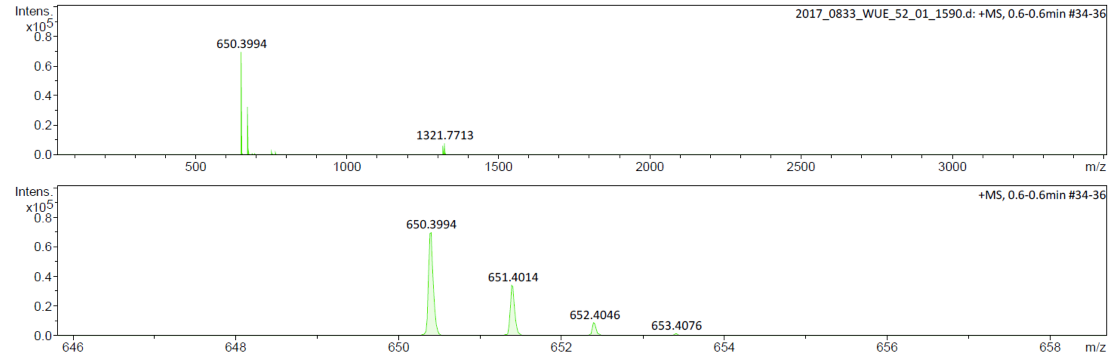

Meas. m/z	#	Ion Formula	m/z	err [ppm]	mSigma	# mSigma	Score	rdb	e¯ Conf	N-Rule
650.3994	1	C46H52NO2	650.3993	-0.2	10.2	1	100.00	21.5	even	ok

Figure S60. HRMS (ESI-TOF, pos. mode, acetonitrile/chloroform 1/1) spectra of **6d**.

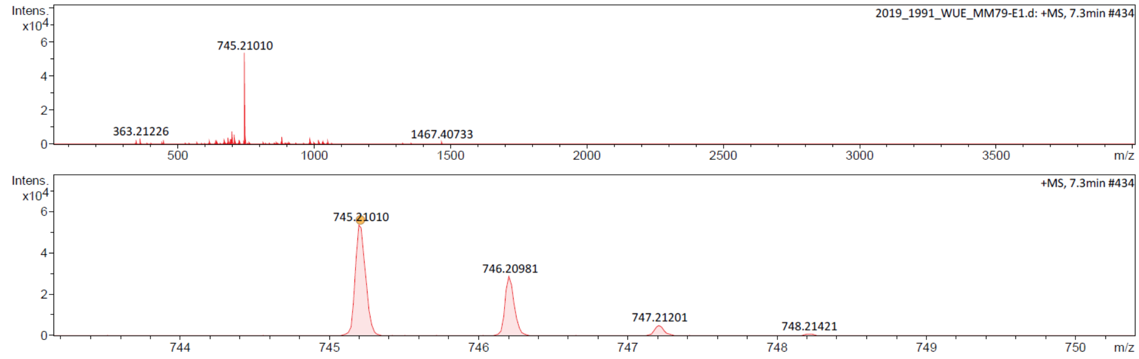

Meas. m/z	#	Ion Formula	m/z	err [ppm]	mSigma	# mSigma	Score	rdb	e¯ Conf	N-Rule
745.21010	1	C50H30N2NaO4	745.20978	-0.43	33.3	1	100.00	36.5	even	ok

Figure S61. HRMS (ESI-TOF, pos. mode, acetonitrile/chloroform 1/1) spectra of **7a**.

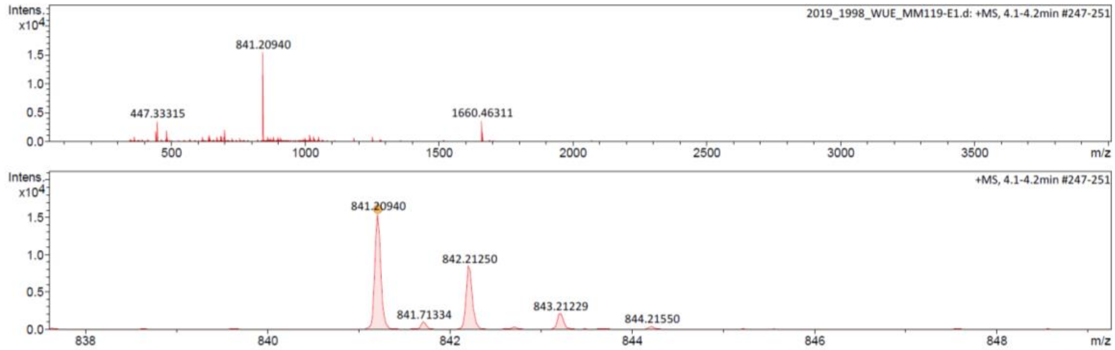

Meas. m/z	#	Ion Formula	m/z	err [ppm]	mSigma	# mSigma	Score	rdb	e⁻ Conf	N-Rule
841.20940	1	C58H30N2NaO4	841.20978	0.45	58.2	1	100.00	44.5	even	ok

Figure S62. HRMS (ESI-TOF, pos. mode, acetonitrile/chloroform 1/1) spectra of **7b**.

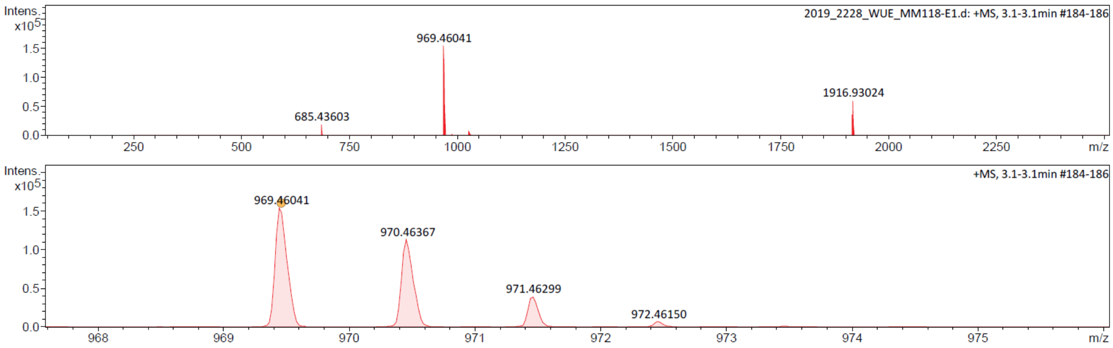

Meas. m/z	#	Ion Formula	m/z	err [ppm]	mSigma	# mSigma	Score	rdb	e⁻ Conf	N-Rule
969.46041	1	C66H62N2NaO4	969.46018	-0.24	14.1	1	100.00	36.5	even	ok

Figure S63. HRMS (ESI-TOF, pos. mode, acetonitrile/chloroform 1/1) spectra of **7c**.

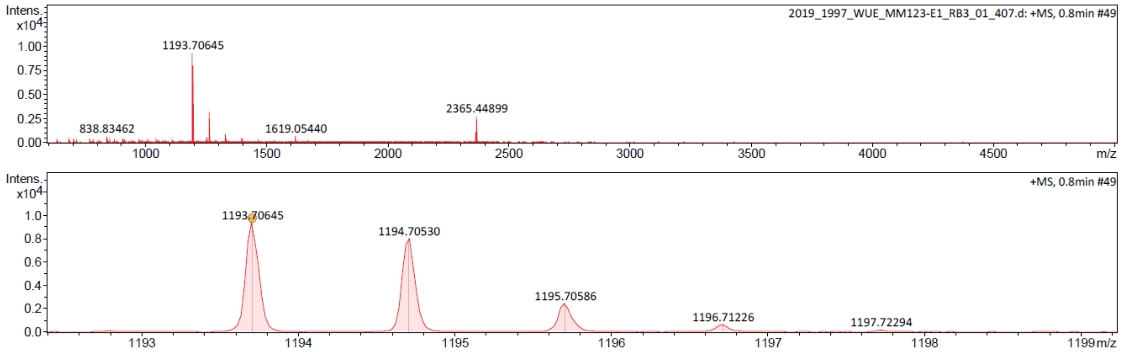

Meas. m/z	#	Ion Formula	m/z	err [ppm]	mSigma	# mSigma	Score	rdb	e⁻ Conf	N-Rule
1193.70645	1	C82H94N2NaO4	1193.71058	3.46	74.8	1	100.00	36.5	even	ok

Figure S64. HRMS (ESI-TOF, pos. mode, acetonitrile/chloroform 1/1) spectra of **7d**.

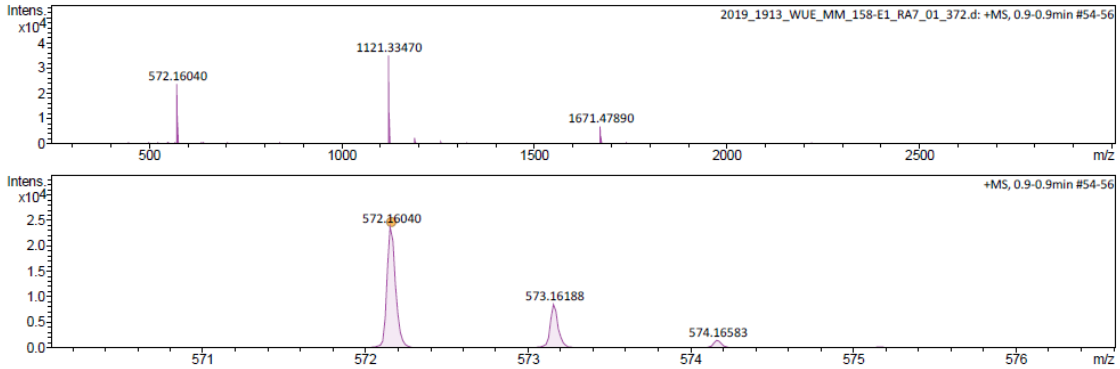

Meas. m/z	#	Ion Formula	m/z	err [ppm]	mSigma	# mSigma	Score	rdb	e⁻ Conf	N-Rule
572.16040	1	C40H23NNaO2	572.16210	2.96	42.4	1	100.00	29.5	even	ok

Figure S65. HRMS (ESI-TOF, pos. mode, acetonitrile/chloroform 1/1) spectra of **8a**.

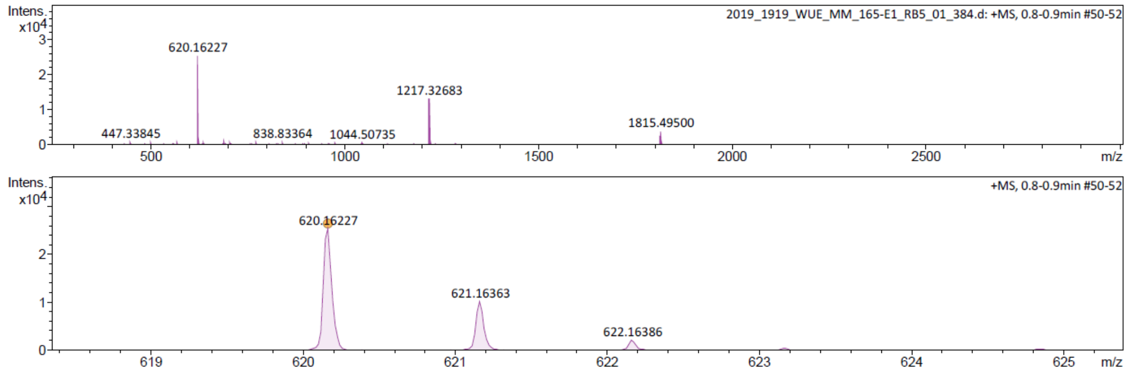

Meas. m/z	#	Ion Formula	m/z	err [ppm]	mSigma	# mSigma	Score	rdb	e⁻ Conf	N-Rule
620.16227	1	C44H23NNaO2	620.16210	-0.27	46.1	1	100.00	33.5	even	ok

Figure S66. HRMS (ESI-TOF, pos. mode, acetonitrile/chloroform 1/1) spectra of **8b**.

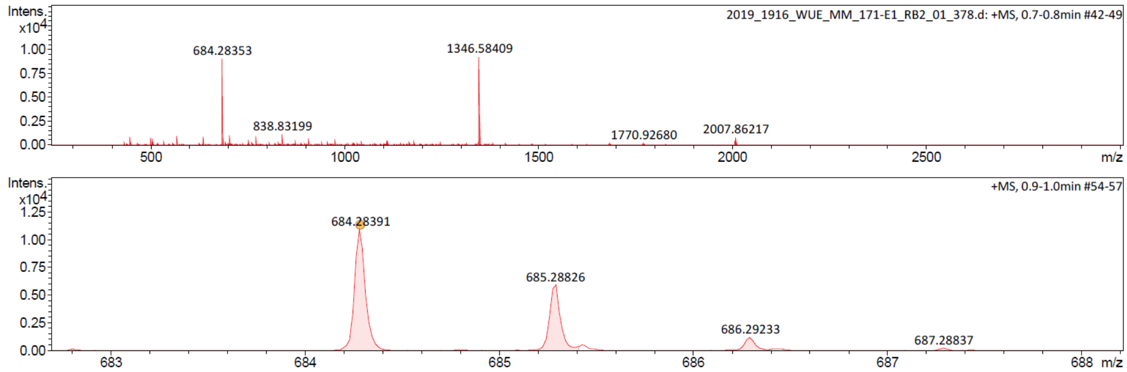

Meas. m/z	#	Ion Formula	m/z	err [ppm]	mSigma	# mSigma	Score	rdb	e⁻ Conf	N-Rule
684.28391	1	C48H39NNaO2	684.28730	4.95	18.1	1	100.00	29.5	even	ok

Figure S67. HRMS (ESI-TOF, pos. mode, acetonitrile/chloroform 1/1) spectra of **8c**.

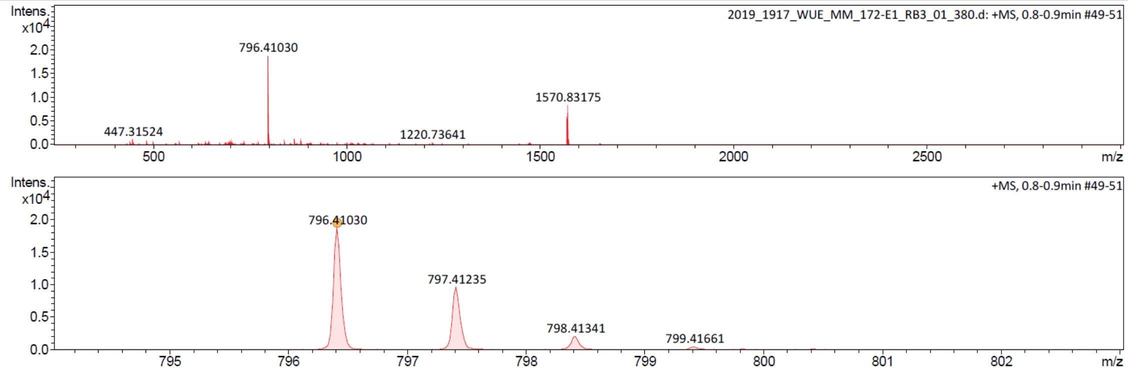

Meas. m/z	#	Ion Formula	m/z	err [ppm]	mSigma	# mSigma	Score	rdb	e⁻ Conf	N-Rule
796.41030	1	C56H55NNaO2	796.41250	2.76	66.0	1	100.00	29.5	even	ok

Figure S68. HRMS (ESI-TOF, pos. mode, acetonitrile/chloroform 1/1) spectra of **8d**.

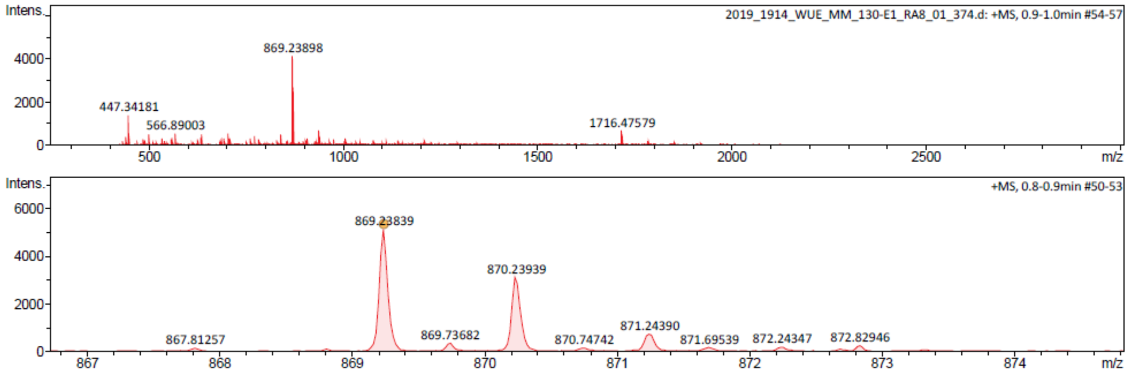

Meas. m/z	#	Ion Formula	m/z	err [ppm]	mSigma	# mSigma	Score	rdb	e⁻ Conf	N-Rule
869.23839	1	C60H34N2NaO4	869.24108	3.09	43.7	1	100.00	44.5	even	ok

Figure S69. HRMS (ESI-TOF, pos. mode, acetonitrile/chloroform 1/1) spectra of **9a**.

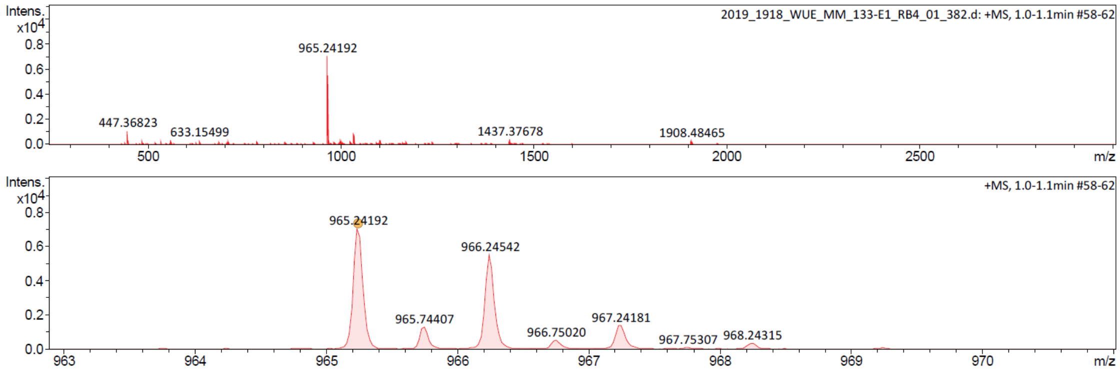

Meas. m/z	#	Ion Formula	m/z	err [ppm]	mSigma	# mSigma	Score	rdb	e⁻ Conf	N-Rule
965.24192	1	C68H34N2NaO4	965.24108	-0.87	43.6	1	100.00	52.5	even	ok

Figure S70. HRMS (ESI-TOF, pos. mode, acetonitrile/chloroform 1/1) spectra of **9b**.

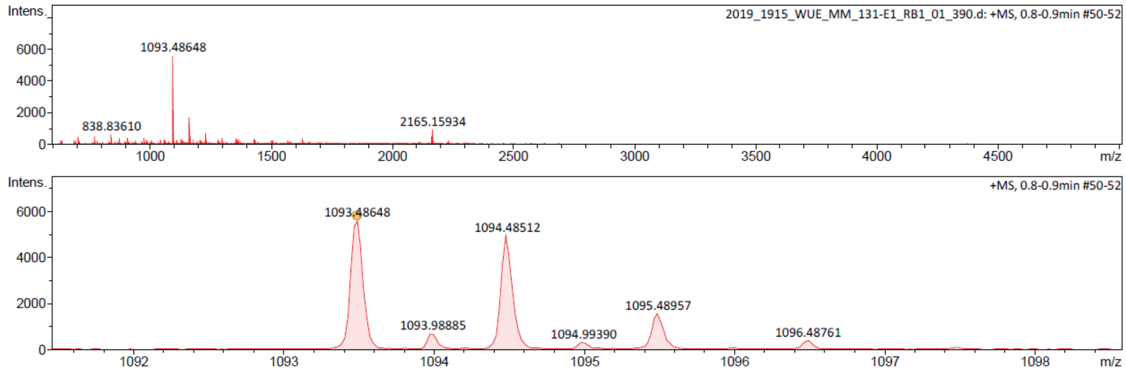

Meas. m/z	#	Ion Formula	m/z	err [ppm]	mSigma	# mSigma	Score	rdb	e⁻ Conf	N-Rule
1093.48648	1	C76H66N2NaO4	1093.49148	4.57	43.8	1	100.00	44.5	even	ok

Figure S71. HRMS (ESI-TOF, pos. mode, acetonitrile/chloroform 1/1) spectra of **9c**.

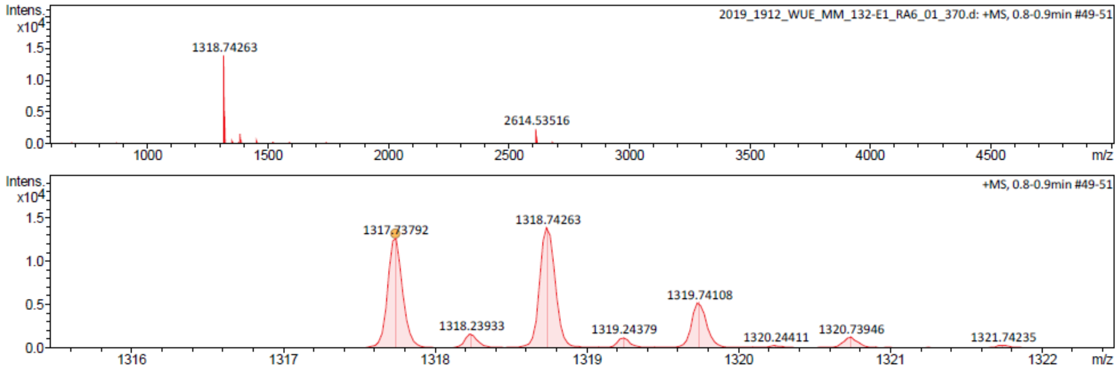

Meas. m/z	#	Ion Formula	m/z	err [ppm]	mSigma	# mSigma	Score	rdb	e⁻ Conf	N-Rule
1317.73792	1	C92H98N2NaO4	1317.74188	3.00	79.7	1	100.00	44.5	even	ok

Figure S72. HRMS (ESI-TOF, pos. mode, acetonitrile/chloroform 1/1) spectra of **9d**.

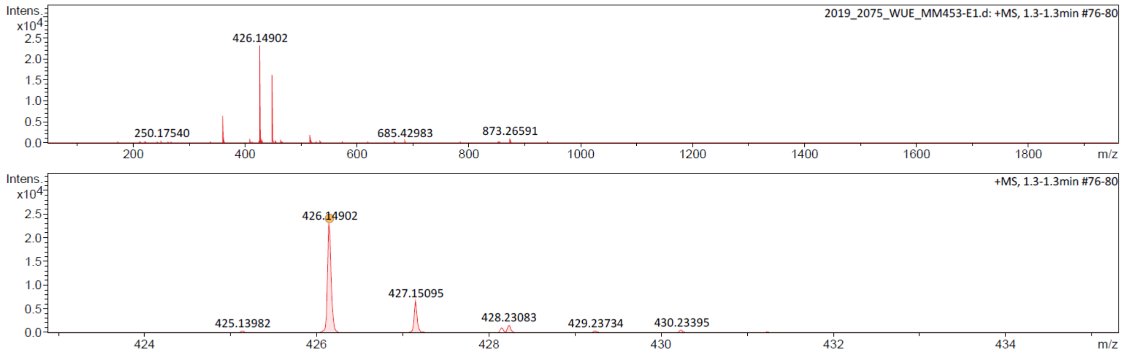

Meas. m/z	#	Ion Formula	m/z	err [ppm]	mSigma	# mSigma	Score	rdb	e⁻ Conf	N-Rule
426.14902	1	C30H20NO2	426.14886	-0.38	41.3	1	100.00	21.5	even	ok

Figure S73. HRMS (ESI-TOF, pos. mode, acetonitrile/chloroform 1/1) spectra of **10a**.

References

[S1] G. M. Sheldrick, *Acta Cryst. A* **2015**, *71*, 3-8.
[S2] Y. Miura, H. Oka, M. A. Momoki, *Synthesis* **1995**, *11*, 1419-1422.
[S3] D. Meinhard, M. Wegner, G. Kipiani, A. Hearley, P. Reuter, S. Fischer, O. Marti, B. Rieger, *J. Am. Chem. Soc.* **2007**, *129*, 9182-9191.
[S4] I. Gottker-Schnetmann, P. Wehrmann, C. Rohr, S. Mecking, *Organometallics* **2007**, *26*, 2348-2362.
[S5] Y. Li, C. Wang, C. Li, S. Di Motta, F. Negri, Z. H. Wang, *Org. Lett.* **2012**, *14*, 5278-5281.
[S6] H. Langhals, L. Feiler, US5650513A, **1995**.
[S7] R. H. Mitchell, M. Chaudhary, R. V. Williams, R. Fyles, J. Gibson, M. J. Ashwoodsmith, A. J. Fry, *Can. J. Chem.* **1992**, *70*, 1015-1021.
